# IL27 and IL1RN are causally associated with acute pancreatitis: a Mendelian randomization study

**DOI:** 10.18632/aging.205825

**Published:** 2024-05-13

**Authors:** Qingxu Jing, Xuxu Liu, Zhenyi Lv, Dongbo Xue

**Affiliations:** 1 Department of General Surgery, The First Affiliated Hospital of Harbin Medical, University, Harbin 150001, China; 2 Key Laboratory of Hepatosplenic Surgery, Ministry of Education, The First Affiliated Hospital of Harbin Medical University, Harbin 150001, China

**Keywords:** interleukins, acute pancreatitis, IL27, IL1RN, Mendelian randomization

## Abstract

Background: The interleukin (IL) plays a role in the development of acute pancreatitis (AP). However, the specific IL in AP has not been fully revealed. Therefore, the association between prospective IL and AP was studied via Mendelian randomization (MR).

Methods: The HUGO Gene nomenclature committee (HGNC) database provided 47 interleukin related genes (ILRGs). ILRGs and differentially expressed genes (DEGs) from GSE194331 were overlapped to create differently expressed ILRGs (DE-ILRGs). The integrative epidemiology unit (IEU) open genome-wide association study (GWAS) database provided exposure and outcome datasets. Univariate MR (UVMR) analysis using MR-Egger, IVW, simple mode, and weighted mode was done. UVMR results were verified using sensitivity analysis. Drug prediction, MVMR analysis, and PPI network development were also performed.

Results: Six DE-ILRGs were obtained. IL27 and IL1RN were substantially causally linked with AP by UVMR analysis (OR = 0.926, P < 0.001 and OR = 1.031, P = 0.023). Our sensitivity analysis showed the dependability of our results. Direct effect of IL27 was suggested by MVMR analysis. In the cytokine receptor binding pathway, IL27 and IL1RN interacted with IL36G and IL1R2. TAE-684, ARQ-680, and 12 other IL1RN and 14 IL27 medications were predicted.

Conclusions: IL1RN was identified as a risk factor for acute pancreatitis (AP), but IL27 was found to be a protective factor for AP.

## INTRODUCTION

Acute pancreatitis (AP) is a prevalent gastrointestinal condition that often requires hospitalization due to its sudden onset, severe nature, and quick progression. Its recurrent occurrences can ultimately result in pancreatic failure and cerebral palsy [1]. While the majority of patients experience a self-limiting condition, approximately 20% of them progress to moderate or severe acute pancreatitis, with a fatality rate ranging from 20 to 40% [2]. Recognized risk factors for acute pancreatitis include smoking, hypertriglyceridemia, and autoimmune disorders [3]. Recent Mendelian randomization (MR) studies have found causal associations between acute pancreatitis and gallstones, type 2 diabetes mellitus (T2DM), smoking, body fat percentage, body mass fat index, trunk fat percentage, percent limb fat, and plasma triglycerides [4–6]. Researchers have thoroughly examined the causes and development of AP in recent years and have identified the significant involvement of the Interleukin (IL) family in AP [7]. Thus, identifying adjustable risk factors of the IL family for AP is crucial for the prompt treatment of the condition.

IL is a cytokine that is crucial for immune control and maintaining homeostasis, generated by multiple cells [8]. Increased IL-33 expression is associated with the seriousness of AP, and excessive IL-33 production worsens the inflammatory reaction and damage to the pancreas [9]. Overexpression of IL-6 leads to a sustained and exacerbated inflammatory response, which promotes the occurrence of pancreatic injury [10]. IL-17 not only exacerbates the damage to pancreatic acinar cells but also works together with other proinflammatory mediators [11]. Serum IL-18 levels are positively associated with polymorphonuclear neutrophil elastase (PMN-E), serving as an early and sensitive indicator for predicting the severity of AP [12]. There is a lack of research investigating the causal association between IL gene family and AP utilizing MR.

MR is essentially an instrumental variable method originated from econometrics. MR can be utilized to investigate the causation hypothesis, which is a genetic instrumental variable analysis, used to evaluate the possible connection between exposure and result [13]. Since MR employs instrumental variable analysis to mimic the randomization process of causal inference in randomized controlled trials (RCT), the design is less vulnerable to reverse causal bias [14]. In this study, the MR analysis was used to investigate the causal link between IL and AP based on the Open Genome-Wide Association Study (GWAS) database, offering a novel sight on the correlation between the IL gene family and AP.

## RESULTS

### IL27 and IL1RN were identified as key genes

**Figure 1:**
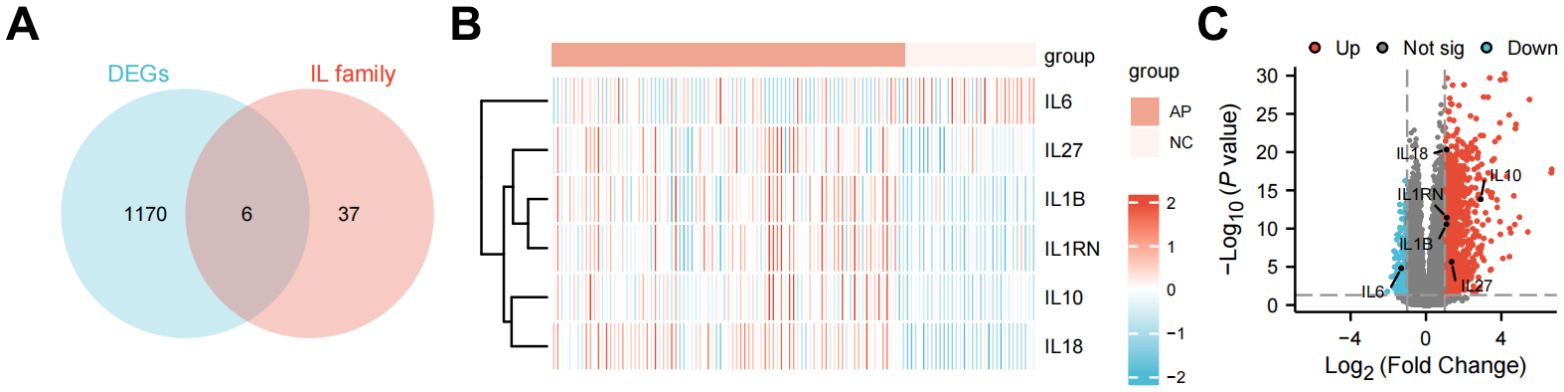
**Differently expressed interleukin-related genes analysis for acute pancreatitis.** (**A**) Venn diagram depicting genes associated with different expression genes only, IL family only, or both. (**B**) Gene expression heatmap of the differently expressed interleukin-related genes. Log2-transformed average expression in Illumina HiSeq 2500. (**C**) Volcano plot of different expression genes from GSE194331.

Based on the screening criteria of |log2-fold change (FC)| > 1 and P-value < 0.05, DEGs including 994 up-regulated and 183 down-regulated were identified in AP patients’ samples compared with health controls’ samples (Supplementary Table 1). A total of 6 DE-ILRGs were obtained, including IL1B, IL1RN, IL6, IL10, IL18 and IL27 (Figure 1A). Volcano plot and bidirectional hierarchical clustering heatmap were described to the DE-ILRGs (Figures 1B, 1C), which clearly indicated the samples tend to cluster in two distinct directions.

### IL27 and IL1RN were causally associated with AP

**Figure 2:**
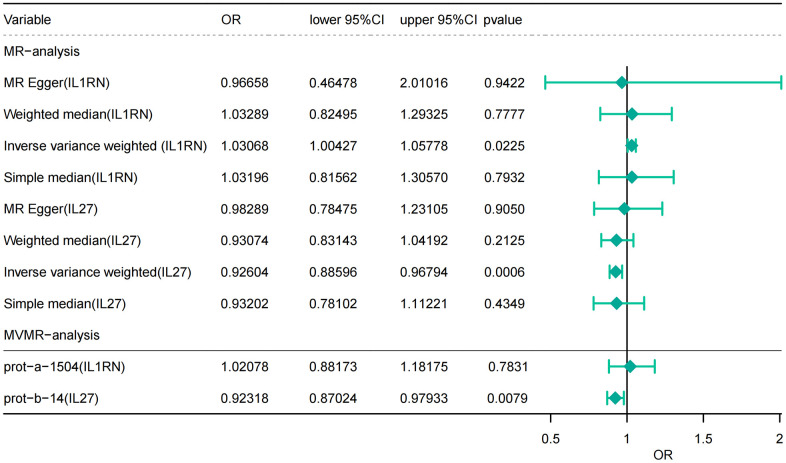
**Causal estimates of genetically predicted IL-27 and IL-1RN using univariate and multivariate Mendelian randomization analysis.** OR, odds ratios; CI, confidence interval.

Following screening, a total of 3 SNPs for IL27, 2 SNPs for IL1B, 3 SNPs for IL1RN, 1 SNP for IL10, 2 SNPs for IL18, and 4 SNPs for IL6 were identified as instrumental variables in the finn-b-K11_ACUTP dataset for UVMR analysis (Supplementary Table 2). The IVW approach results indicated that IL27 (P < 0.001, OR = 0.926) and IL1RN (P = 0.023, OR = 1.031) were significantly causally related to AP as shown in Figure 2A and Table 1.

**Table 1:** MR results.

**Exposure**	**Outcome**	**Method**	**nsnp**	**b**	**se**	**P-value**
prot-b-14	finn-b-K11_ACUTPANC	MR Egger	3	-0.01726	0.114862	0.9050455
prot-b-14	finn-b-K11_ACUTPANC	Weighted median	3	-0.07177	0.0575697	0.2125206
prot-b-14	finn-b-K11_ACUTPANC	Inverse variance weighted (multiplicative random effects)	3	-0.076834	0.0225784	0.0006665
prot-b-14	finn-b-K11_ACUTPANC	Simple median	3	-0.070402	0.0901814	0.4349939
prot-a-1504	finn-b-K11_ACUTPANC	MR Egger	3	-0.033993	0.3735759	0.9422313
prot-a-1504	finn-b-K11_ACUTPANC	Weighted median	3	0.0323649	0.1146915	0.7777968
prot-a-1504	finn-b-K11_ACUTPANC	Inverse variance weighted (multiplicative random effects)	3	0.0302148	0.0132439	0.0225236
prot-a-1504	finn-b-K11_ACUTPANC	Simple median	3	0.031463	0.1200375	0.7932366

### IL27 as a protective factor and IL1RN as a risk factor

**Figure 3:**
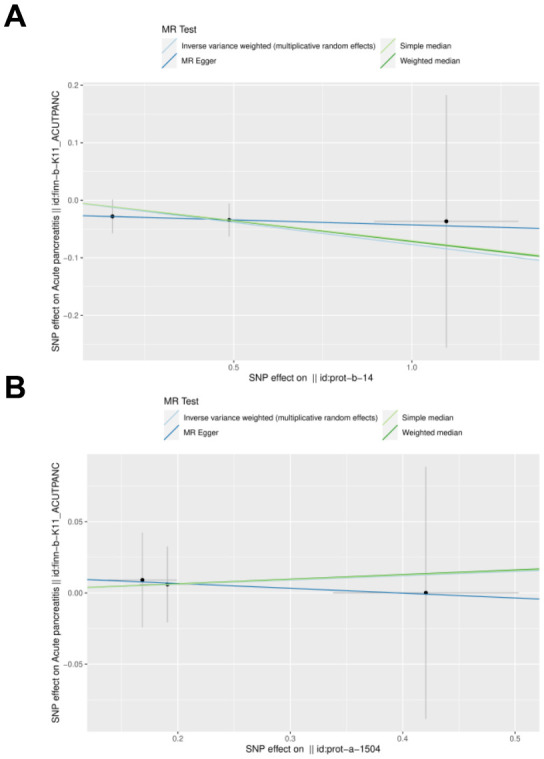
**Protein-protein interaction network and drug prediction for differently expressed interleukin-related genes.** (**A**) Gene related IL-27 and IL-1RN regulation network. (**B**) Drug regulatory network associated with IL-27 and IL-1RN.

**Figure 4:**
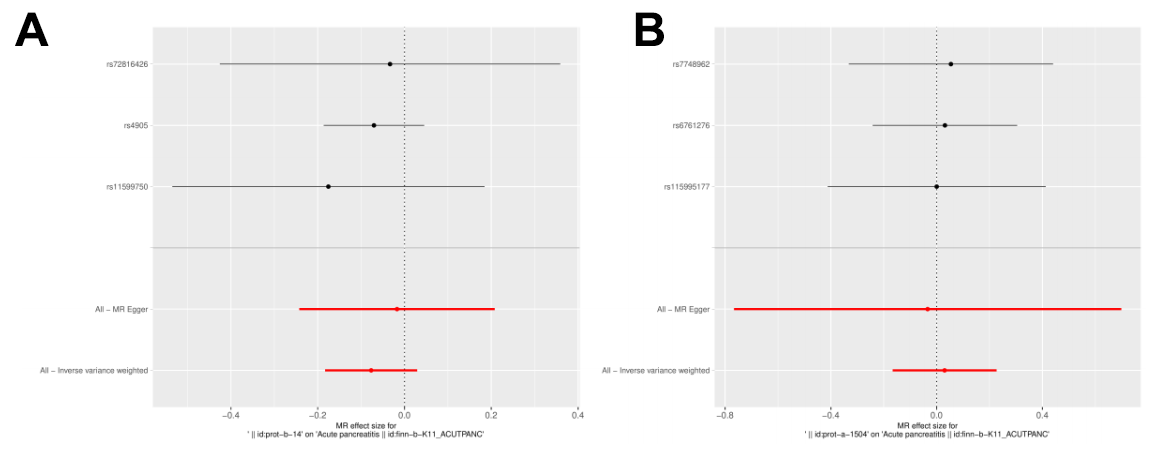
**Scatter plot and effect estimates for four main estimators.** (**A**) All 3 SNPs are plotted together with 95% confidence intervals representing their effect on both IL-27 (horizontal access) and on AP (vertical axis). (**B**) All 3 SNPs are plotted together with 95% confidence intervals representing their effect on both IL-1RN (horizontal access) and on AP (vertical axis).

**Figure 5:**
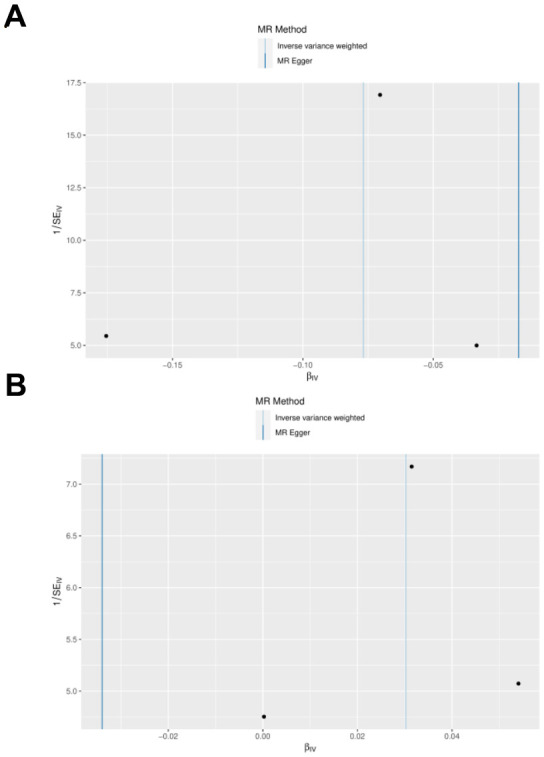
**Forest plot of the causal effects of IL-1RN and IL-27 on acute pancreatitis.** (**A**) MR estimate for elevated IL-27 on AP. (**B**) MR estimate for elevated IL-1RN on AP.

IL27 was identified as a protective factor and IL1RN was identified as a risk factor. The scatter plot showed a negative slope of the straight line for AP and IL27, suggesting that IL27 was a protective factor. Besides, a positive slope of the straight line for AP and IL1RN, suggesting that IL1RN was a risk factor (Figures 3A, 3B). The results of forest plots also confirmed the above results (Figures 4A, 4B). Meanwhile, the funnel plot showed that UVMR conformed to the second law of MR grouping (Figures 5A, 5B).

### The reliability and sensitivity analysis

**Figure 6:**
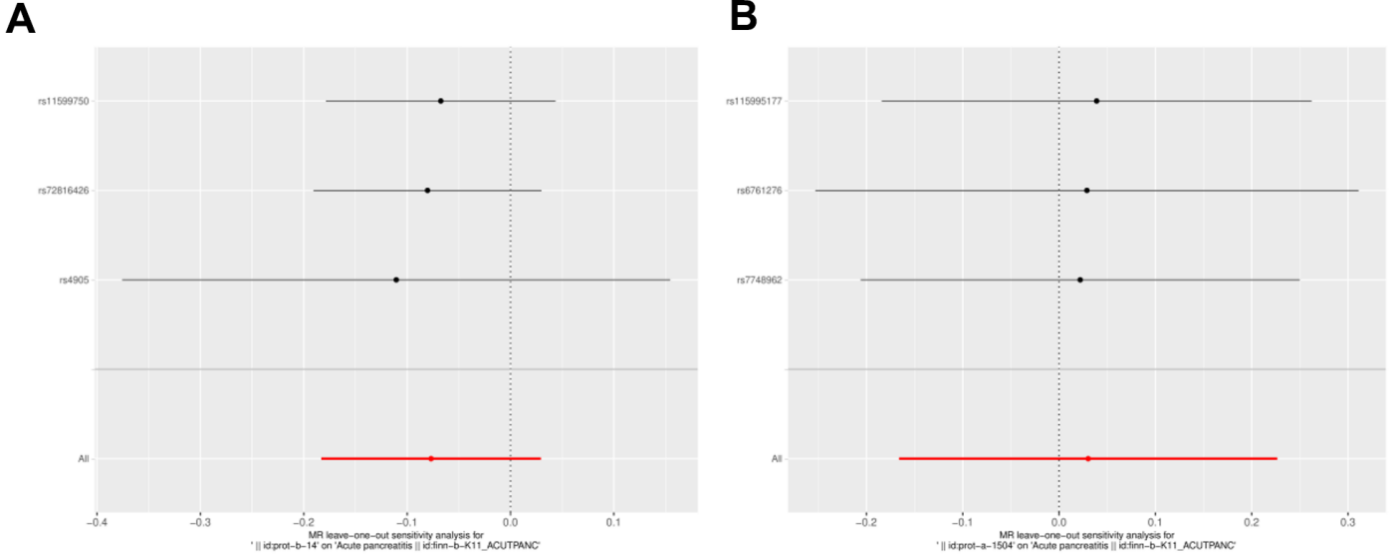
**Funnel diagram of the random judgment of SNPs on IL-1RN and IL-27.** (**A**) MR random judgment for elevated SNPs on IL-27. (**B**) MR random judgment for elevated SNPs on IL-1RN.

A sensitivity analysis was conducted to verify the reliability of the results. The Q-value for heterogeneity and the p-value for horizontal pleiotropy were both greater than 0.05, indicating that there was neither heterogeneity nor horizontal pleiotropy in the UVMR analysis (Table 2). Furthermore, there were no significant biases in the LOO analysis results, indicating that they were reliable (Figures 6A, 6B).

**Table 2:** MR sensitivity.

**Gene**	**P-value of heterogeneity**	**P-value of pleiotropy**
IL1RN	0.9826626	0.887608
IL27	0.8405166	0.6615243

### IL27 as a direct influence in MVMR

MVMR analysis results showed that IL1RN (P = 0.783, OR = 1.182) and IL27 (P = 0.008, OR = 0.980) attributes had not changed. However, IL1RN changed from a significant factor to a non-significant factor after correcting for the effect of IL27 (Figure 2B and Table 3).

**Table 3:** MVMR results.

	**OR**	**95%CI low**	**95%CI high**	**P-value**
prot-a-1504(IL1RN)	1.0207758	0.881725741	1.1817543	0.7831426
prot-b-14(IL27)	0.9231765	0.870239402	0.9793338	0.0079752

### Construction of PPI network and drug prediction

**Figure 7:**
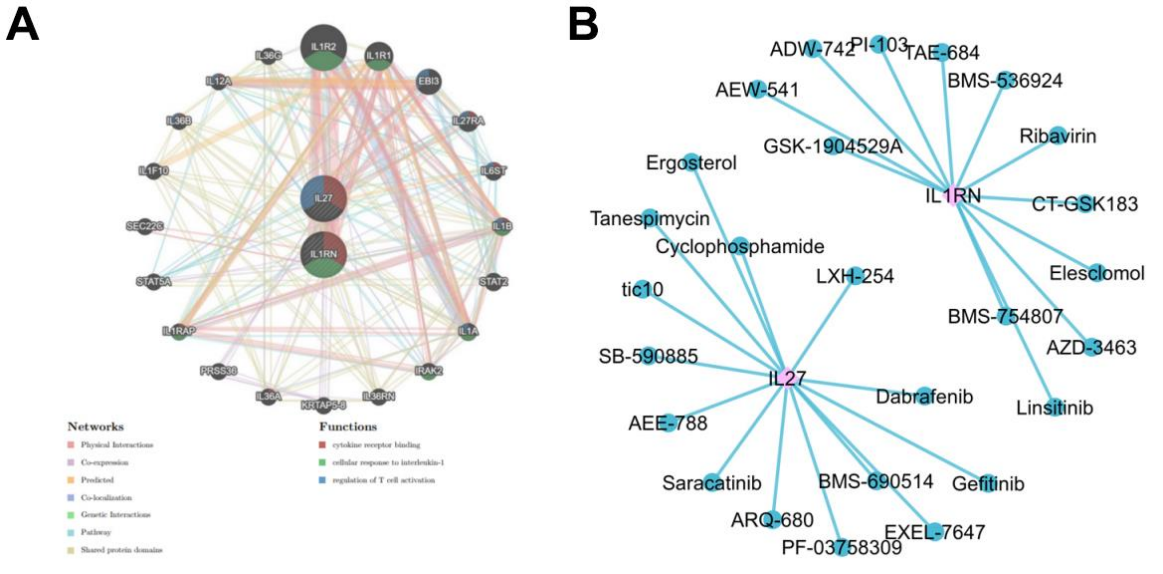
**Forest plot of the sensitivity analysis of IL-1RN and IL-27 on acute pancreatitis.** (**A**) MR sensitivity analysis for elevated IL-27 on AP. (**B**) MR sensitivity analysis for elevated IL-1RN on AP.

The results of the PPI regulatory network showed that IL27 and IL1RN were enriched in pathways including cytokine receptors binding, cellular response to interleukin-1 and regulation of T cell activation, and interacted with IL36G and IL1R2 (Figure 7A and Supplementary Tables 3, 4). The drug prediction results showed that the gene-drug network includes 28 nodes and 26 edges. Among them, IL1RN gene predicted 12 drugs (TAE-684 and CT-GSK183, etc.) and IL27 gene predicted 14 drugs (ARQ-680 and EMS-690514, etc.) (Figure 7B).

## DISCUSSION

This study found that IL-27 and IL-1RN were causally associated with AP. Moreover, IL-27 reduced the risk of disease, and IL-1RN was associated with increased risk of disease. AP is one of the most common acute diseases of the digestive system, which is characterized by local and systemic inflammation with a different clinical course [15]. Although the treatment of AP has evolved to be multidisciplinary, individualized and minimally invasive over the past decade, the mortality rate from severe acute pancreatitis (SAP) remains high [16]. The main reason for this is the release of a range of inflammatory response factors in the blood, leading to the occurrence of systemic inflammatory response syndrome and even multi-organ dysfunction syndrome [17]. Moreover, the reported risk factors for AP are almost based on the observational studies and case reports with small sample size [1]. This MR study provided convincing evidence, suggesting that a genetically predicted IL-1RN is causally associated with increased risk of AP whereas genetically predicted IL-27 level might exhibit protective effect on AP.

IL-27, a pleiotropic immunomodulatory cytokine, was first identified in 2002 [18]. IL-27 is expressed in multiple innate immune cells [19]. Dendritic cells located in the lamina propria of the intestine are also major producers of IL-27 after induction by Faecalis [20]. In addition to its multiple regulatory roles in innate immunity, IL-27 has been shown to have multiple functions in adaptive immunity, particularly its regulatory role in biology of T cells. With further study, the researchers found that IL-27 was mainly induced the differentiation of helper T cell subsets [21]. In previous experiments, IL-27 was highly expressed in AP, and the expression of IL-27 decreased significantly after the treatment of IFN-γ [22]. SNP of IL-27 was associated with genetic susceptibility to rheumatoid arthritis in the Chinese Han and Polish individuals, implying that IL-27 may be involved in the development of rheumatoid arthritis [23, 24]. A recent study in patients with Crohn’s disease reported higher levels of IL-27 in serum than in health controls [25]. Our study first reports that IL-27 plays a protective role in the development and progression of AP.

Earlier studies have found that IL-1 antagonism significantly attenuated pancreatic amylase release and tissue necrosis during pancreatitis [26]. IL-1 receptor antagonists encoded by IL-1RN, is member of the IL-1 gene cluster, which comes in two forms including a secreted form in macrophages and an intracellular form in epithelial cells [27]. Abnormal expression of IL-1RN has also been associated with sepsis, acute myeloid leukemia, and inflammatory bowel disease [28–30]. IL-1RN polymorphisms have been reported to be involved in the pathogenesis of type 2 diabetes mellitus [31]. Although IL-1RN expression is not necessary for the development of pancreatitis, it promotes the expansion of pancreatic damage and its associated inflammation [32]. IL-1RN appears to determine the severity of AP and the susceptibility to idiopathic AP [33]. We demonstrated for the first time from MR methods that IL-1RN can serve as a contributing factor for the development in AP.

To perform the MR analysis, three core assumptions need to be proved [34]. Evaluating the first presumption by DE-ILRGs linear regression of genetic variation and calculating the f-statistic, we found that all f-statistics for included variants were greater than 20, which suggests that the genetic variants used in our study were strongly in association with DE-ILRGs (P < 5×10^-7^, f-statistic > 20). Secondly, presumption requires that genetic instruments are independent of confounding factors in either DE-ILRGs or AP (Supplementary Table 2). To test this presumption, we utilized the PhenoScanner GWAS database to eliminate SNPs associated with available confounder (P < 1×10^-7^). Finally, we assume that genetic tools affect the AP only through DE-ILRG. The MR-Egger regression was performed to potentially test for pleiotropy and the result showed that there was no pleiotropy, suggesting that the third presumption may not be transgressed. The main advantage of this study is the MR design, which minimizes residual confounding and reduces reverse causation. Another advantage is MVMR, which can explain the relationship between IL-27 and IL-1RN. Our work may be constrained by gene pleiotropy, where a genetic variant has multiple direct correlations, thereby undermining conclusions drawn from a single route hypothesis. Our results were consistent across all MR approaches, and the Leave-One-Out (LOO) method was employed to identify potential pleiotropy.

Furthermore, we constructed a PPI network to select two genes in the AP group, which may play a key role in AP. Some studies have shown that through cellular response to IL-1, IL-1RN allelic polymorphism was strongly associated with an increased risk of T2DM, and the IL-1B allele polymorphism was associated with a decreased risk of T2DM [35]. IL-27 signaling in Tregs plays a crucial role for the proteinogenic properties of Tregs by upregulating CD39 [36]. Dabrafenib and Linsitinib have been tentatively applied to pancreatic ductal adenocarcinoma with poor prognosis after conventional cytotoxic chemotherapy [37, 38]. We found DE-ILRGs related drugs like Dabrafenib and Linsitinib from the CellMiner database, which can provide a new chance for treatment of acute pancreatitis.

Our Mendelian randomization analysis supports a possible causal link between IL and AP, addressing some information gaps. In a univariate analysis, there was a significant association between AP and IL-27 and IL1RN. IL-1RN was found to have a causal influence on AP, whereas IL-27 was found to have a preventive effect on AP. However, in multivariate analysis, after adjusting for the influence of IL 27, IL1RN transitioned from being a significant factor to an inconsequential factor. IL 27 directly contributes to AP when two exposure variables are present simultaneously. The validation of additional prospective experimental investigations and further clinical study involving a large number of samples is still required.

## MATERIALS AND METHODS

### Data screening

We scanned the Gene Expression Omnibus database for high throughput screening data using the following parameters in order to screen the differentially expressed genes (DEGs) that were present between the health controls and the AP patients: a) the human species; b) the sample size is greater than one hundred; c) samples of blood taken from people who are experiencing AP [39]. In conclusion, high throughput screening results of GSE194331, which included 87 AP and 32 controls together with recurrence information, were collected by the use of the platform GPL16791 Illumina HiSeq 2500 [40].

### Genetic instrument selection

The HUGO Gene nomenclature committee (HGNC) database was used in order to acquire interleukin related genes (ILRGs) [41]. Prot-b-14 of IL27, prot-a-1504 of IL1RN, prot-a-1495 of IL1B, prot-a-1539 of IL6, prot-a-1464 of IL10, prot-b-21 of IL18, and finn-b-K11_ACUTPANC of AP were among the datasets that were obtained from the GWAS database maintained by the Integrative Epidemiology Unit (IEU). [42]. Each of the 3,349 samples that were included in the prot-b-14 dataset contained a total of 5,270,646 single nucleotide polymorphisms (SNPs). The dataset known as prot-a-1504 contained 10,534,735 SNPs that were derived from 3,301 samples. A total of 16,380,428 SNPs were included in the finn-b-K11_ACUTPANC dataset. These SNPs were derived from 3,022 cases and 195,144 control samples.

### Identification of DE-ILRGs and selection of IVs

Using the limma R package, we identified DEGs by analyzing the intersection of 87 AP samples and 32 healthy control samples. The criterion for identifying DEGs was established at a P-value of less than 0.05 and a log2-fold change (FC) greater than 1 [43]. Using the ComplexHeatmap R package (Version: 2.13.1), a hierarchical cluster heatmap that is based on Euclidean distance was constructed. This heatmap reflects the expression intensity and direction of DEGs of IL [44]. Through the process of overlapping ILRGs and the DEGs from GSE194331, differently expressed (DE)-ILRGs were produced. This allowed for the identification of IL genes that were strongly linked with disease. With the help of the TwoSampleM program (version 0.5.6), the SNPs that were found to be substantially linked with DE-ILRGs, were selected as instrumental variables (IVs) in this investigation. The criterion for identifying these SNPs was set at P < 5*10^-7^ [45]. Linkage disequilibrium analysis (LDA) was then used to eliminate single nucleotide polymorphisms (SNPs) (r^2^ = 0.001 and kb = 10000). The DE-ILRGs that had a P-value of less than 0.05 during the inverse variance weighted (IVW) analysis were determined to be the important genes. Furthermore, in order to do MR analysis, it was necessary to have three fundamental premises: (1) there was a strong and significant connection between intravenous (IV) and exposure; (2) there was no relationship between IVs and confounding factors; and (3) IVs could only influence outcomes through exposure and not through any other channels.

### Multivariable (MV) MR analysis of exposure factors

MVMR analysis was carried out in order to make a discovery about the causal relationships that exist between important genes and AP at the multivariate level. The processing of data for the MVMR analysis was comparable to that of the UVMR study (p < 5*10^-7^, r^2^ = 0.001, and kb = 10000 are similar) [46]. All analyses were performed using the TwoSampleMR package.

### Functional analysis of exposure factors

Through the use of the GeneMANIA website, a protein-protein interaction (PPI) network was established for key genes in order to determine whether or not there were connections between critical genes [47]. In addition, the gene-drug networks were constructed with the help of the CellMiner database [48].

### Statistical analyses

In order to establish statistically significant causal associations between AP and DE-ILRGs (p < 0.05), the univariable Mendelian randomization (UVMR) study was conducted using MR-Egger, weighted median, IVW, and simple mode [49–51]. In the field of MR analysis, the IVW approach was the most significant. Based on the results of the odds ratio (OR) calculation, it was determined that the exposure factor was a risk factor if it was greater than one, while the exposure factor was a protection factor if it was greater than one. Scattered plots, forest plots, and funnel plots were utilized in the presentation of the acquired results. In addition, sensitivity analysis was carried out in order to assess the overall dependability of the UVMR data. There was no evidence of heterogeneity when the Q value was more than 0.05, as determined by the heterogeneity tests that were carried out. Horizontal pleiotropy tests were carried out by MR-Egger, and when the value of P was larger than 0.05, it indicated that there was no horizontal pleiotropy in the study. The leave-one-out (LOO) analysis was carried out by gradually removing each SNP. If there was no significant change in the influence of the remaining SNPs on the outcome variable, this might be interpreted as a hint that the results of the UVMR study were valid [52].

## Supplementary Material

Supplementary Table 1

Supplementary Table 2

Supplementary Table 3

Supplementary Table 4
