# Supplementary material for: IL27 and IL1RN are causally associated with acute pancreatitis: a Mendelian randomization study
Source: Aging (Albany NY). 2024 May 13;16(10):8572–84. doi: 10.18632/aging.205825 (PMC11164491; doi:10.18632/aging.205825)
Supplement: Supplementary Table 1 [file aging-16-205825-s001.doc]

Supplementary Table 1. DEGs identified in AP patient samples.

| GENE_ID | baseMean | log2FoldChange | lfcSE | stat | pvalue |
| --- | --- | --- | --- | --- | --- |
| TRIM9 | 1138.162497 | 4.198044036 | 0.367241705 | 11.4312835 | 2.91764E-30 |
| SLC36A1 | 1323.520902 | 1.421299034 | 0.126068959 | 11.27398092 | 1.76409E-29 |
| RN7SL526P | 175.2569807 | 3.062043601 | 0.279438499 | 10.95784444 | 6.09334E-28 |
| TRIQK | 30.33750438 | 1.271332208 | 0.116405386 | 10.92159261 | 9.08879E-28 |
| SLPI | 2104.334713 | 5.498536416 | 0.505024259 | 10.8876679 | 1.31977E-27 |
| PAPPA2 | 1692.962723 | 1.22412408 | 0.115974702 | 10.55509568 | 4.81149E-26 |
| TNFAIP6 | 388.5802303 | 1.669081931 | 0.166356278 | 10.03317667 | 1.08954E-23 |
| HGF | 247.5385024 | 1.114029535 | 0.112706903 | 9.88430616 | 4.86943E-23 |
| PDCD1LG2 | 200.5162613 | 1.099660977 | 0.111536262 | 9.8592239 | 6.25308E-23 |
| RAB30 | 167.0037082 | 1.211700509 | 0.12323462 | 9.832468376 | 8.15939E-23 |
| RNY1P16 | 556.8094959 | 3.870471324 | 0.395571684 | 9.784500469 | 1.31242E-22 |
| SRGN | 194.7444026 | 1.712947034 | 0.176765996 | 9.690478221 | 3.30975E-22 |
| PHC2 | 49.30085383 | 2.418566988 | 0.251286272 | 9.624747759 | 6.2861E-22 |
| MTND4P22 | 21.83800486 | 2.413841306 | 0.252374329 | 9.564527882 | 1.12715E-21 |
| IFITM10 | 3018.567864 | 1.609773496 | 0.168916146 | 9.530015562 | 1.5726E-21 |
| HNRNPA3P2 | 846.6833311 | 2.189980402 | 0.230793159 | 9.488931171 | 2.33415E-21 |
| MORC1 | 474.3294675 | 1.616246304 | 0.170790198 | 9.463343454 | 2.98251E-21 |
| SLC12A5-AS1 | 63.04915254 | 2.630787942 | 0.278127675 | 9.458921848 | 3.11135E-21 |
| MIR5690 | 219.264178 | 2.181703908 | 0.232751382 | 9.373537931 | 7.01411E-21 |
| MYL6 | 189.9988411 | 2.158562872 | 0.230811508 | 9.35205912 | 8.59577E-21 |
| RCVRN | 1833.398827 | 2.021863583 | 0.217148888 | 9.310955268 | 1.26688E-20 |
| LMNB1 | 4292.099318 | 1.127008537 | 0.121155567 | 9.302160566 | 1.3762E-20 |
| UPB1 | 361.0872554 | 1.267281715 | 0.136762036 | 9.266326795 | 1.92667E-20 |
| MIR4772 | 47.40158161 | 1.259241216 | 0.135921981 | 9.264441285 | 1.96101E-20 |
| IGKV1-6 | 34.26503173 | 1.172334142 | 0.126889886 | 9.238988026 | 2.48839E-20 |
| TCN1 | 63.3367661 | 1.26997297 | 0.137727825 | 9.220888896 | 2.94647E-20 |
| IL18R1 | 2897.559492 | 1.208374296 | 0.13141988 | 9.194760291 | 3.75832E-20 |
| TRAJ22 | 119.7541895 | 1.132119897 | 0.123582076 | 9.16087458 | 5.14784E-20 |
| CST7 | 289.526265 | 1.039309468 | 0.114240913 | 9.097524152 | 9.24144E-20 |
| H2AC13 | 66.52548259 | 1.204726313 | 0.132671929 | 9.080491416 | 1.08086E-19 |
| FGR | 599.7940578 | 1.268975251 | 0.140669107 | 9.020994597 | 1.86389E-19 |
| LNCATV | 74.8252998 | 1.345411073 | 0.149654886 | 8.990091171 | 2.47025E-19 |
| ANXA1 | 3218.165838 | 1.193857007 | 0.133192501 | 8.963395059 | 3.14832E-19 |
| ADGRG3 | 21.20524081 | 1.487210235 | 0.166299646 | 8.942954885 | 3.789E-19 |
| CHRNA9 | 23.34139569 | 2.956997354 | 0.33075198 | 8.940225718 | 3.88376E-19 |
| KCNJ2 | 3929.191338 | 1.214281991 | 0.136480226 | 8.897127658 | 5.73106E-19 |
| SNX3 | 155.5445412 | 1.106295839 | 0.124401587 | 8.892939903 | 5.9513E-19 |
| TMIGD3 | 4207.636774 | 1.818783858 | 0.204606911 | 8.889161413 | 6.15718E-19 |
| ADAM9 | 22.59018855 | 1.504768284 | 0.170717264 | 8.814388493 | 1.2034E-18 |
| SLC11A1 | 162.378498 | 1.159085307 | 0.131503203 | 8.814122265 | 1.20626E-18 |
| CKAP4 | 229.4528995 | 1.559284579 | 0.177615631 | 8.778982868 | 1.6496E-18 |
| MAPK10 | 945.2863736 | 1.957993577 | 0.223809443 | 8.748485115 | 2.16236E-18 |
| HCK | 1055.899152 | 1.649395725 | 0.189005156 | 8.726723432 | 2.62157E-18 |
| KANK1 | 860.1781293 | 1.94229551 | 0.222994893 | 8.710044816 | 3.03755E-18 |
| MCTP1 | 48.06002027 | 1.320774135 | 0.151971771 | 8.690917581 | 3.59524E-18 |
| SCARNA7 | 61.24210895 | 1.045617646 | 0.120429472 | 8.682406607 | 3.87481E-18 |
| DSP | 195.0443892 | 1.132074612 | 0.130555153 | 8.671236496 | 4.27453E-18 |
| H2AC8 | 83.70464749 | 1.190129114 | 0.137289239 | 8.668772014 | 4.36806E-18 |
| FCGBP | 41.18792675 | 1.718707532 | 0.199190495 | 8.628461571 | 6.21827E-18 |
| AQP10 | 31.05733474 | 1.347547526 | 0.156323854 | 8.620229664 | 6.682E-18 |
| NECAB1 | 152.1795891 | 2.004061767 | 0.233468764 | 8.583853944 | 9.17464E-18 |
| GNG10 | 66.30367705 | 1.171585058 | 0.136641487 | 8.574153318 | 9.98184E-18 |
| EDAR | 637.5116462 | 1.223110589 | 0.142815649 | 8.564261676 | 1.08771E-17 |
| LDHA | 91.13557468 | 1.749459319 | 0.204520178 | 8.553969272 | 1.18927E-17 |
| INHBA | 43.24904783 | 1.088753238 | 0.127290171 | 8.553317406 | 1.196E-17 |
| VAPA | 25.43769248 | 1.192416041 | 0.139573163 | 8.543304584 | 1.30437E-17 |
| KREMEN1 | 56.56910098 | 2.008394737 | 0.23555529 | 8.526213668 | 1.51216E-17 |
| DOCK8-AS1 | 70.12432945 | 1.2473502 | 0.146333992 | 8.5239949 | 1.54142E-17 |
| CAPG | 35.79807331 | 2.707046718 | 0.320440911 | 8.447881119 | 2.96637E-17 |
| NCKAP5 | 1727.093542 | 1.622615157 | 0.192461529 | 8.430854545 | 3.4315E-17 |
| QSOX1 | 793.3343324 | 1.846396437 | 0.21992796 | 8.395460221 | 4.64073E-17 |
| MTND6P3 | 28.05056367 | 1.126158519 | 0.134299257 | 8.385441184 | 5.05359E-17 |
| CHMP5 | 251.2622704 | 1.843099757 | 0.219977137 | 8.378596912 | 5.35624E-17 |
| MRO | 50.35611481 | 1.098633423 | 0.131271113 | 8.369194118 | 5.80136E-17 |
| SNORA74B | 136.7974069 | 1.652011319 | 0.197422822 | 8.367884211 | 5.8662E-17 |
| NAIPP3 | 100.7455337 | 1.837022514 | 0.219577709 | 8.366161226 | 5.95257E-17 |
| SHISA2 | 573.5852866 | 1.890009233 | 0.227635613 | 8.302783603 | 1.017E-16 |
| SLC5A9 | 1730.896108 | 1.178165619 | 0.142197625 | 8.285409981 | 1.17703E-16 |
| EPB41L4B | 142.9009871 | 1.4438788 | 0.175438339 | 8.230121227 | 1.87024E-16 |
| ZFY-AS1 | 21.36287514 | 1.486691581 | 0.180959742 | 8.215592966 | 2.11118E-16 |
| POTEF | 15.55425607 | 1.905128462 | 0.232209438 | 8.204354118 | 2.31834E-16 |
| MS4A4A | 35701.22825 | 1.098871708 | 0.134120079 | 8.193193102 | 2.54386E-16 |
| H3C8 | 48.04878805 | 1.424420786 | 0.173939068 | 8.189194079 | 2.62981E-16 |
| GABRR2 | 1352.882398 | 1.138062348 | 0.139382371 | 8.165037927 | 3.21334E-16 |
| NDST3 | 75.68396906 | 1.61558168 | 0.19818173 | 8.152021267 | 3.57891E-16 |
| OSM | 5843.630051 | 1.025924713 | 0.125874032 | 8.150407979 | 3.62699E-16 |
| IDI1 | 617.8340138 | 1.178639998 | 0.144635892 | 8.149014605 | 3.66902E-16 |
| AIM2 | 36.78745463 | 1.959295101 | 0.240474533 | 8.147619937 | 3.71157E-16 |
| KLHDC7A | 17.14860831 | 2.421647628 | 0.297719127 | 8.134000832 | 4.1535E-16 |
| CD207 | 133.3067147 | 1.80042344 | 0.221966648 | 8.11123407 | 5.01082E-16 |
| ST3GAL4 | 18.07166531 | 2.110415072 | 0.260644299 | 8.096916297 | 5.63698E-16 |
| ADAM22 | 693.7171146 | 1.594953457 | 0.197506742 | 8.075438032 | 6.72348E-16 |
| SLC8A1-AS1 | 59.55850835 | 1.937765009 | 0.240387597 | 8.061002446 | 7.56713E-16 |
| RNA5SP68 | 7481.038485 | 1.693226828 | 0.210100692 | 8.059120659 | 7.68452E-16 |
| TFPI | 10631.84989 | 1.172936892 | 0.145744188 | 8.047915357 | 8.42162E-16 |
| TRAJ11 | 935.4176278 | 1.209835997 | 0.150407707 | 8.043710153 | 8.71585E-16 |
| MYBPC3 | 192.2163637 | 1.063705813 | 0.132269995 | 8.041928289 | 8.84356E-16 |
| RN7SL688P | 9.379060941 | 1.48461241 | 0.185285838 | 8.012551978 | 1.12352E-15 |
| LRFN2 | 168.3312253 | 2.327921218 | 0.290546066 | 8.012227643 | 1.12649E-15 |
| RNU4-38P | 285.5635161 | 1.652332471 | 0.206325694 | 8.008369852 | 1.16239E-15 |
| BMX | 104.356384 | 2.049606317 | 0.255991276 | 8.006547536 | 1.17974E-15 |
| GPER1 | 31.65026933 | 1.386049172 | 0.174163792 | 7.958308413 | 1.74407E-15 |
| C4orf3 | 11.24579509 | 1.616643894 | 0.203293584 | 7.952262235 | 1.83136E-15 |
| SERPINC1 | 191.588703 | 1.715786119 | 0.215810149 | 7.950442222 | 1.85847E-15 |
| PIK3CD-AS1 | 13.81199982 | 2.232024848 | 0.281746722 | 7.922096957 | 2.33538E-15 |
| SLC9A7P1 | 57.65011399 | 1.310364288 | 0.16582077 | 7.902292887 | 2.73819E-15 |
| CCNJL | 909.8454731 | 1.10172387 | 0.139794055 | 7.881049494 | 3.24643E-15 |
| FRMD4B | 18810.5262 | 1.210340785 | 0.153634345 | 7.878061282 | 3.325E-15 |
| QPCT | 235.0125679 | 1.76059455 | 0.223671801 | 7.871329971 | 3.50891E-15 |
| CD58 | 1893.215537 | 1.123099165 | 0.142774517 | 7.866243831 | 3.65448E-15 |
| MIR26A2 | 2.703736084 | 3.442060648 | 0.438109703 | 7.856618165 | 3.94644E-15 |
| HRH2 | 2866.452846 | 1.44659609 | 0.184351534 | 7.846943621 | 4.26299E-15 |
| NEBL | 512.6493235 | 1.003546145 | 0.128427816 | 7.814087147 | 5.53628E-15 |
| COL17A1 | 826.0065863 | 1.277427226 | 0.163489742 | 7.813500776 | 5.56211E-15 |
| AP3B2 | 925.2695219 | 2.042185101 | 0.261572183 | 7.807348157 | 5.84038E-15 |
| FOLR3 | 25.24985294 | 1.184757628 | 0.151881077 | 7.800561149 | 6.16325E-15 |
| MIR3945 | 48.54807857 | 1.470067721 | 0.188781007 | 7.787159005 | 6.85327E-15 |
| FAM169A | 25.70450708 | 1.229350107 | 0.157886989 | 7.786266059 | 6.90185E-15 |
| CCDC70 | 9.809255528 | 2.760967783 | 0.354709343 | 7.783746985 | 7.04075E-15 |
| SMPDL3A | 21.37230001 | 1.224630679 | 0.157397082 | 7.780517029 | 7.22287E-15 |
| ERLIN1 | 9.541674189 | 3.487195625 | 0.449123654 | 7.76444437 | 8.20039E-15 |
| RAB31 | 23.56626781 | 1.657164392 | 0.213641191 | 7.756764419 | 8.71236E-15 |
| STK3 | 649.3306303 | 1.335234311 | 0.17251278 | 7.739915315 | 9.94831E-15 |
| BAMBI | 2543.608953 | 1.105098312 | 0.143112076 | 7.721908176 | 1.14601E-14 |
| CNTNAP3 | 796.1099342 | 1.284532544 | 0.166605393 | 7.710029772 | 1.25788E-14 |
| PRKG2 | 60.25672365 | 1.125128022 | 0.145964534 | 7.708228794 | 1.27576E-14 |
| TRAJ48 | 43.67050809 | 1.092805415 | 0.141847306 | 7.704097102 | 1.31772E-14 |
| PYGL | 2269.289282 | 1.452131546 | 0.188584299 | 7.700172048 | 1.35883E-14 |
| CEBPD | 49.75791228 | 1.236315242 | 0.160887613 | 7.684340756 | 1.53787E-14 |
| HOXB9 | 9.602312836 | 3.770037546 | 0.491784037 | 7.666042938 | 1.77384E-14 |
| UBTD1 | 58.63496354 | 1.01637228 | 0.132658191 | 7.661587052 | 1.83649E-14 |
| KIF1B | 2875.275099 | 1.464836367 | 0.191226358 | 7.660222062 | 1.85612E-14 |
| SLC26A8 | 5660.0858 | 1.571193153 | 0.205244371 | 7.655231388 | 1.92964E-14 |
| SH2D6 | 15.71763841 | 1.152705443 | 0.150672564 | 7.650400392 | 2.00354E-14 |
| TMEM119 | 17.54686372 | 2.414198473 | 0.315977746 | 7.640406651 | 2.16537E-14 |
| ALOX5 | 180.4496369 | 1.782336433 | 0.234171482 | 7.611244611 | 2.71469E-14 |
| SLC1A2 | 369.7780283 | 1.112140625 | 0.14621752 | 7.606069529 | 2.82557E-14 |
| NFATC2 | 492.5121719 | 1.376455527 | 0.180999878 | 7.60473178 | 2.85495E-14 |
| RPS2P14 | 25.10505023 | 1.088067845 | 0.143229784 | 7.596659128 | 3.03874E-14 |
| TRIM55 | 10.62994586 | 1.813866516 | 0.23884361 | 7.59436903 | 3.09296E-14 |
| HAUS4 | 65.69932672 | 1.409298642 | 0.186051946 | 7.574758925 | 3.59795E-14 |
| FIGN | 176.6399878 | 2.058038877 | 0.272013871 | 7.565933577 | 3.85089E-14 |
| RN7SL552P | 29.7301362 | 1.342648999 | 0.177487708 | 7.564743557 | 3.88631E-14 |
| HTATIP2 | 16.27565881 | -1.036548777 | 0.137332497 | -7.547731232 | 4.42906E-14 |
| CAPZA2 | 15.48862675 | 1.263586495 | 0.167447816 | 7.546150967 | 4.4831E-14 |
| GAS7 | 6.449115401 | 2.523831564 | 0.334770606 | 7.538987954 | 4.73633E-14 |
| CTSL | 133.4701538 | 1.299353706 | 0.172603176 | 7.527982604 | 5.15303E-14 |
| IFNGR1 | 2855.123396 | 1.194175581 | 0.158683826 | 7.525502836 | 5.25178E-14 |
| SIPA1L2 | 75.54146567 | -1.057668659 | 0.140764063 | -7.51376902 | 5.74489E-14 |
| IL27 | 55.90259247 | 2.194420294 | 0.292062395 | 7.513532481 | 5.75529E-14 |
| FCAR | 6.756029524 | 1.916038327 | 0.255464959 | 7.500200159 | 6.37205E-14 |
| ENTPD7 | 14.28554779 | -1.383237895 | 0.184776632 | -7.486000162 | 7.10043E-14 |
| LSMEM2 | 8.788299011 | 1.228926832 | 0.164661766 | 7.463340515 | 8.4356E-14 |
| IFITM3 | 15.39305999 | 1.929194126 | 0.258569829 | 7.461017919 | 8.58566E-14 |
| OVCH1 | 127.5872344 | 1.082362707 | 0.145153866 | 7.456657776 | 8.87448E-14 |
| CD177 | 18.27959676 | 1.172116402 | 0.157917051 | 7.422354915 | 1.15056E-13 |
| TUB | 530.9987466 | 1.180813441 | 0.159098234 | 7.421914203 | 1.1544E-13 |
| MIR6131 | 59.15940675 | 1.160729524 | 0.156867666 | 7.399418584 | 1.36782E-13 |
| NCF4 | 3076.684339 | 1.098454246 | 0.148516179 | 7.396192497 | 1.40145E-13 |
| PHTF1 | 1671.797638 | 1.503172285 | 0.203273561 | 7.394824384 | 1.41595E-13 |
| CICP27 | 319.1004753 | 1.307806977 | 0.177321175 | 7.375357054 | 1.63905E-13 |
| BEND7 | 14.34640732 | 1.730583658 | 0.234787872 | 7.37083922 | 1.69557E-13 |
| SCN1B | 111.7872534 | -1.053744165 | 0.143163137 | -7.360443368 | 1.833E-13 |
| KLF14 | 247.2300704 | 1.14323376 | 0.155436222 | 7.355002236 | 1.90924E-13 |
| TACSTD2 | 71.61157498 | 1.517255224 | 0.206357468 | 7.352557873 | 1.94449E-13 |
| LRRC75B | 15.82816286 | 1.251233077 | 0.170391094 | 7.343300909 | 2.08389E-13 |
| SPATC1 | 15.37377291 | 1.301937715 | 0.177710157 | 7.326186284 | 2.36795E-13 |
| TLR4 | 74.1599178 | 1.358062831 | 0.185442961 | 7.323345262 | 2.41864E-13 |
| SLC9A4 | 19.51600847 | 1.707495789 | 0.233178811 | 7.322688453 | 2.43052E-13 |
| BCL6 | 292.0242885 | 1.045565782 | 0.142873243 | 7.318135717 | 2.5144E-13 |
| PLA2G4A | 4012.685914 | 1.031122527 | 0.141268831 | 7.299009396 | 2.89894E-13 |
| RARA-AS1 | 327.4284237 | 1.583512076 | 0.217264009 | 7.28842334 | 3.13603E-13 |
| ARHGEF28 | 5.415399682 | 2.173720954 | 0.299073923 | 7.268172811 | 3.64382E-13 |
| AGTRAP | 15.45382469 | 1.269694823 | 0.174838039 | 7.262120036 | 3.8107E-13 |
| PADI2 | 2855.639589 | 1.21488963 | 0.167613636 | 7.248155112 | 4.22487E-13 |
| NSMCE1-DT | 92.29741389 | 1.009977823 | 0.139433844 | 7.243419489 | 4.37511E-13 |
| ATP2C2 | 32.97987658 | 2.120671747 | 0.293800641 | 7.218063709 | 5.2733E-13 |
| GRAMD1A | 87.5039304 | 1.625063238 | 0.22530126 | 7.212845755 | 5.47945E-13 |
| ACSL1 | 7.226121216 | 1.504852105 | 0.208867678 | 7.204810819 | 5.81245E-13 |
| COX6B2 | 38.1159649 | 1.246750923 | 0.173065248 | 7.203935721 | 5.8499E-13 |
| TSPAN16 | 10.91116272 | -1.363561829 | 0.189340509 | -7.201638124 | 5.94934E-13 |
| DDIAS | 232.7551586 | 1.907268925 | 0.26490454 | 7.199834795 | 6.02856E-13 |
| ATP6V1D | 731.541298 | 1.031211934 | 0.143245274 | 7.19892464 | 6.06893E-13 |
| RAD54B | 6.572529191 | 2.046026401 | 0.284611028 | 7.188851454 | 6.53386E-13 |
| TXN | 19.16055868 | 1.802761855 | 0.251296637 | 7.173839956 | 7.29229E-13 |
| GPR141 | 9.784704121 | 2.2341472 | 0.311433904 | 7.173744323 | 7.29738E-13 |
| STOM | 106.0115914 | 1.446586821 | 0.201723303 | 7.171143841 | 7.43737E-13 |
| MTCO3P5 | 41.01054395 | 2.112503817 | 0.295503647 | 7.148824857 | 8.75239E-13 |
| GPR174 | 201.6642676 | 1.70637256 | 0.238760686 | 7.146790316 | 8.88304E-13 |
| LILRB3 | 37.94022711 | 2.11949661 | 0.296609255 | 7.145753449 | 8.95036E-13 |
| IFTAP | 26.10103561 | 1.27876295 | 0.179053873 | 7.14177764 | 9.21316E-13 |
| TGFBR3 | 96.84576934 | 1.828424539 | 0.256507146 | 7.128162184 | 1.01718E-12 |
| MSL3 | 1332.040354 | 1.44907971 | 0.203291851 | 7.128075746 | 1.01782E-12 |
| SCN9A | 64.88828064 | 1.257375998 | 0.176934672 | 7.106442078 | 1.19073E-12 |
| ENTPD1 | 909.577854 | 1.221133806 | 0.171995307 | 7.099808857 | 1.2493E-12 |
| NOL3 | 50.92754924 | 1.167235118 | 0.164493833 | 7.095920239 | 1.28494E-12 |
| non-functional | 12.73460054 | 1.217590639 | 0.171651009 | 7.093408 | 1.30849E-12 |
| DBI | 8.021071307 | 1.933187459 | 0.272553338 | 7.092877563 | 1.31352E-12 |
| RETN | 15.65569278 | 1.343699353 | 0.189731052 | 7.082126729 | 1.41959E-12 |
| H2BC5 | 9.356572152 | 1.940149765 | 0.274547662 | 7.06671386 | 1.58646E-12 |
| TMEM200B | 7.125853457 | 1.177730729 | 0.166893628 | 7.056774669 | 1.70412E-12 |
| IL4R | 12.97150771 | 2.002612555 | 0.283866489 | 7.054769162 | 1.72887E-12 |
| Hsp70 | 23.08817551 | 1.009117594 | 0.14311274 | 7.051207255 | 1.77372E-12 |
| APMAP | 833.6098184 | 1.452474432 | 0.206106668 | 7.047197679 | 1.82557E-12 |
| GASK1A | 10.32589309 | 1.485812438 | 0.211072266 | 7.039354169 | 1.93133E-12 |
| NOP10 | 7.941052284 | 2.161344463 | 0.308063333 | 7.015909483 | 2.28458E-12 |
| RNA5SP248 | 187.076053 | 1.741495975 | 0.248280531 | 7.014226876 | 2.31224E-12 |
| OOSP1 | 22.96919678 | 1.549645906 | 0.22106109 | 7.010034668 | 2.38259E-12 |
| H4C8 | 12.79443317 | 1.601958715 | 0.228938595 | 6.997329213 | 2.60888E-12 |
| ERCC6L | 35.10966983 | 1.127081206 | 0.16146982 | 6.980135393 | 2.94896E-12 |
| HMGB1P19 | 81.21219704 | 1.009712902 | 0.144834591 | 6.971489995 | 3.13602E-12 |
| IL1B | 10.7067963 | 2.236637513 | 0.321080474 | 6.965971755 | 3.26145E-12 |
| RAB5IF | 9.738693283 | 4.967361324 | 0.713165065 | 6.965233672 | 3.27859E-12 |
| TMEM54 | 104.3947101 | 1.425923341 | 0.20472996 | 6.964898251 | 3.28641E-12 |
| TEX46 | 8.65910769 | 1.035968188 | 0.148932622 | 6.955952145 | 3.50189E-12 |
| KRT80 | 233.4375562 | 1.133973854 | 0.163365768 | 6.941318668 | 3.88457E-12 |
| DYNLT5 | 51.67899239 | 1.002616697 | 0.145281662 | 6.901192352 | 5.15679E-12 |
| TMT1B | 14.59201154 | 1.14731143 | 0.166740458 | 6.880822115 | 5.95081E-12 |
| DACH1 | 3.078565736 | 2.013389915 | 0.293001325 | 6.871606868 | 6.34827E-12 |
| ORM1 | 96.76094936 | 1.106080367 | 0.161015757 | 6.869392079 | 6.44761E-12 |
| MTHFS | 11.60537725 | 1.493636274 | 0.217553528 | 6.865603546 | 6.62107E-12 |
| PNPLA1 | 10.9406617 | 3.30767638 | 0.483018188 | 6.847933393 | 7.49244E-12 |
| AOAH-IT1 | 77.97009294 | 1.104049812 | 0.16163532 | 6.830498492 | 8.46201E-12 |
| MTCYBP11 | 213.1794902 | 1.080492729 | 0.158555933 | 6.814584019 | 9.45369E-12 |
| LRRN3 | 46.79648488 | 1.174421049 | 0.172507281 | 6.807950627 | 9.89988E-12 |
| FBLN2 | 70.1678618 | 1.096608965 | 0.161117927 | 6.806250466 | 1.00175E-11 |
| CEACAM4 | 10911.58758 | 1.120665412 | 0.164892256 | 6.79634954 | 1.07303E-11 |
| KLLN | 31.23788134 | 1.183501956 | 0.17496747 | 6.764125676 | 1.34117E-11 |
| MS4A6A | 1714.795851 | 1.523349015 | 0.225487037 | 6.755816368 | 1.42033E-11 |
| MGST1 | 5910.017425 | 1.022530643 | 0.151358684 | 6.755678726 | 1.42168E-11 |
| C10orf55 | 7.466133625 | 1.349277984 | 0.200095111 | 6.743183172 | 1.54954E-11 |
| APCDD1 | 10.71513241 | 2.348750948 | 0.348407302 | 6.741394152 | 1.56874E-11 |
| RN7SL141P | 26.16048496 | 1.091006595 | 0.162071298 | 6.731645956 | 1.67754E-11 |
| DHRS9 | 8488.205798 | 1.347927718 | 0.200526699 | 6.721936417 | 1.79325E-11 |
| MND1 | 8.311298437 | 1.048462799 | 0.156017135 | 6.720177246 | 1.81504E-11 |
| RPL7P24 | 23.48639782 | 1.13238989 | 0.169041763 | 6.698876462 | 2.10028E-11 |
| FZD5 | 7.832264122 | 1.478666201 | 0.221454695 | 6.677059618 | 2.43784E-11 |
| TRIM71 | 5.213993038 | 2.044257532 | 0.306170446 | 6.676861078 | 2.44114E-11 |
| TP53I11 | 9.274006322 | 1.129536372 | 0.169237305 | 6.674275342 | 2.48457E-11 |
| TMEM88 | 64.41750658 | 1.717055375 | 0.257501148 | 6.668146473 | 2.59054E-11 |
| HHATL | 2199.381772 | 1.214630714 | 0.182349499 | 6.661003843 | 2.71964E-11 |
| SRGAP1 | 4.306321418 | 4.717941621 | 0.70965061 | 6.648259798 | 2.96578E-11 |
| IL18RAP | 113.2104674 | 2.182488373 | 0.328792178 | 6.63789627 | 3.18192E-11 |
| FAM20A | 257.4941929 | 1.044559603 | 0.157458165 | 6.633886558 | 3.26961E-11 |
| RPL23AP21 | 7.880552971 | 1.371219738 | 0.206766804 | 6.631720924 | 3.31795E-11 |
| PPP1R3B | 23.54478276 | 1.900372957 | 0.287899215 | 6.600827167 | 4.0887E-11 |
| MYT1L | 12.01929143 | 1.034541452 | 0.157419304 | 6.571884312 | 4.96825E-11 |
| IFI27 | 5.636279756 | 1.943130488 | 0.296143204 | 6.561455603 | 5.3285E-11 |
| TRAV24 | 7.182825081 | 1.284543076 | 0.196025353 | 6.552943572 | 5.64138E-11 |
| LOH12CR2 | 3.036959462 | 1.908713058 | 0.291397092 | 6.550213138 | 5.7455E-11 |
| NMNAT2 | 16.44808488 | 1.133515101 | 0.173220588 | 6.543766632 | 5.99884E-11 |
| SNORA14A | 6.546258452 | 1.473608795 | 0.226529977 | 6.505138158 | 7.7622E-11 |
| CA12 | 120.03827 | -1.113761504 | 0.171613965 | -6.489923503 | 8.588E-11 |
| MIR8066 | 10.96068282 | 1.333989269 | 0.205651036 | 6.486664486 | 8.77575E-11 |
| PTEN | 30.69907097 | 1.045166776 | 0.161292464 | 6.479947959 | 9.17543E-11 |
| HMGB1P1 | 36.22691037 | 1.332796936 | 0.205900993 | 6.472999069 | 9.60765E-11 |
| PI3 | 8.15033958 | 4.447981797 | 0.687373333 | 6.470983942 | 9.73668E-11 |
| PTCH1 | 6.790005865 | 1.43005043 | 0.221018666 | 6.470269939 | 9.7828E-11 |
| MMP2 | 22.97298738 | 1.150891827 | 0.177915202 | 6.468766108 | 9.88064E-11 |
| DOK3 | 18.43269702 | 1.102436336 | 0.170515333 | 6.465320825 | 1.01084E-10 |
| RNU6-1013P | 6.388887543 | 1.772856094 | 0.274383376 | 6.461237259 | 1.0385E-10 |
| MTARC1 | 8.587131113 | 1.12830191 | 0.174801165 | 6.454773409 | 1.08381E-10 |
| S100A8 | 748.6036332 | 1.02650724 | 0.159373648 | 6.440884372 | 1.18779E-10 |
| MTCO1P30 | 5.996310458 | 1.925523446 | 0.299011024 | 6.439640316 | 1.19757E-10 |
| PLBD1-AS1 | 12.98310286 | 1.376444915 | 0.213949374 | 6.433507567 | 1.24692E-10 |
| GCKR | 602.6401084 | 1.130168542 | 0.175730922 | 6.43124458 | 1.26563E-10 |
| ATP5F1E | 302.8852575 | 1.105312547 | 0.172245429 | 6.417079129 | 1.38914E-10 |
| PTGES | 8.077997285 | 1.80866307 | 0.283081779 | 6.389189295 | 1.66768E-10 |
| H2AZ1 | 15.78079714 | 1.33994564 | 0.210018573 | 6.380129234 | 1.76939E-10 |
| NR2E1 | 90.8579285 | 1.389894492 | 0.218371241 | 6.364823891 | 1.95514E-10 |
| KIR2DS4 | 8.650763085 | 1.078901438 | 0.169529929 | 6.364076514 | 1.96468E-10 |
| TCAF1P1 | 6.77560733 | 2.18597555 | 0.343914802 | 6.35615431 | 2.06867E-10 |
| SLC4A4 | 36.83518075 | 1.736508892 | 0.273446885 | 6.350443117 | 2.14696E-10 |
| B3GNT5 | 12.25333542 | 1.152298519 | 0.181541562 | 6.347298707 | 2.19128E-10 |
| RSPH9 | 12.13036709 | 1.098209249 | 0.173026568 | 6.347055596 | 2.19475E-10 |
| RNU6-469P | 96.43643838 | 1.608007514 | 0.253453213 | 6.344395859 | 2.233E-10 |
| ZNF415P1 | 13.4862207 | 1.054058464 | 0.166165508 | 6.343425176 | 2.24712E-10 |
| GPRC5C | 3.359927593 | 1.600354588 | 0.252491779 | 6.338244325 | 2.32398E-10 |
| PGM2 | 94.80622232 | 1.150405164 | 0.181683956 | 6.331902872 | 2.42156E-10 |
| RASGRF1 | 1584.68609 | 1.245631753 | 0.196921716 | 6.32551746 | 2.52385E-10 |
| NUP210L | 3.38215313 | 1.759693732 | 0.278645405 | 6.31517227 | 2.69861E-10 |
| AMPH | 910.9218584 | 1.162640617 | 0.184714493 | 6.294257671 | 3.08874E-10 |
| P4HA2 | 398.753026 | 1.293236915 | 0.205735554 | 6.285918444 | 3.25921E-10 |
| LTF | 9.694472613 | 1.483178391 | 0.236336636 | 6.275702398 | 3.48059E-10 |
| RNU4-62P | 3.27538403 | 4.5058459 | 0.718014248 | 6.275426864 | 3.48676E-10 |
| RPL36AP45 | 14.69275558 | 1.272316177 | 0.20284525 | 6.272348893 | 3.55642E-10 |
| RNU6-757P | 4.672754827 | 1.622033492 | 0.258973925 | 6.263308135 | 3.76895E-10 |
| SRPK1 | 19.72191157 | 1.36560867 | 0.218271753 | 6.256460832 | 3.93812E-10 |
| DPY19L3 | 3.178192671 | 2.621372058 | 0.419524539 | 6.248435581 | 4.14584E-10 |
| NMI | 1558.491601 | 1.053090791 | 0.168590042 | 6.24645902 | 4.19862E-10 |
| SEMA6B | 9.839262512 | 1.164689695 | 0.186586718 | 6.242082541 | 4.31783E-10 |
| MTND5P24 | 18.85657856 | 1.36912108 | 0.21936445 | 6.241307928 | 4.33927E-10 |
| RPL10AP6 | 6.554621763 | -1.058887875 | 0.169924216 | -6.231530157 | 4.61901E-10 |
| D-aspartate | 16.05510936 | 1.384927995 | 0.222830828 | 6.215154364 | 5.12742E-10 |
| RNU7-77P | 10.0006703 | 1.509007567 | 0.243132608 | 6.206520714 | 5.41705E-10 |
| UGCG | 2.979310835 | 3.953228531 | 0.637635831 | 6.199821809 | 5.65271E-10 |
| ARPC3 | 70.41450591 | 2.06582161 | 0.334571546 | 6.174528694 | 6.63611E-10 |
| RPSAP22 | 53.60507616 | 1.103834174 | 0.178937693 | 6.168818625 | 6.88021E-10 |
| C4BPA | 384.7201158 | 1.268943256 | 0.205739744 | 6.16771086 | 6.92857E-10 |
| TIMP4 | 8.531111158 | 1.545966086 | 0.251110244 | 6.156523372 | 7.43592E-10 |
| DNAAF4 | 10.09699529 | 1.251814691 | 0.20334071 | 6.156242345 | 7.44912E-10 |
| PTGFR | 31.22971056 | 1.013970957 | 0.165206011 | 6.137615414 | 8.37694E-10 |
| RNU6-1005P | 7.575028358 | 1.363438563 | 0.222299722 | 6.13333453 | 8.60559E-10 |
| GPAT3 | 32.78088613 | 1.241582252 | 0.202809131 | 6.121924825 | 9.24516E-10 |
| ACVR2B-AS1 | 13.27305701 | 1.039406175 | 0.169840814 | 6.119884552 | 9.36432E-10 |
| AIRN | 19.28818724 | 1.008350267 | 0.164879691 | 6.115672964 | 9.61503E-10 |
| SLC2A3 | 448.3507908 | 1.28671467 | 0.210536739 | 6.111592087 | 9.86421E-10 |
| WSB1 | 136.6367743 | 1.131624288 | 0.185347261 | 6.105427632 | 1.02526E-09 |
| G1m marker | 14.76954984 | 1.349222275 | 0.221040413 | 6.10396196 | 1.03471E-09 |
| KCNMA1 | 3.900133577 | 1.815741724 | 0.297666248 | 6.099924777 | 1.06118E-09 |
| HMGB2 | 8.865632796 | 1.098626188 | 0.180689826 | 6.080177353 | 1.2005E-09 |
| FABP5 | 2.45214207 | -1.320610303 | 0.217233217 | -6.079228224 | 1.20762E-09 |
| CATIP | 6.297204475 | 1.105590398 | 0.182175488 | 6.068820858 | 1.28853E-09 |
| SAP30 | 4.23604709 | 2.576421784 | 0.425184364 | 6.059540293 | 1.36511E-09 |
| ANO2 | 32.77592985 | 1.63794336 | 0.270674251 | 6.051345304 | 1.43641E-09 |
| CFAP46 | 3.23006068 | 1.680757716 | 0.278313161 | 6.039088158 | 1.54988E-09 |
| SAMD15 | 12.88920021 | 1.322509503 | 0.219692714 | 6.019815033 | 1.74616E-09 |
| PLIN3 | 65.93447066 | 1.128883357 | 0.187835413 | 6.009960196 | 1.85569E-09 |
| OSBPL1A | 10.28160317 | 1.01591658 | 0.169094353 | 6.00798642 | 1.87842E-09 |
| MOGAT1 | 4.197700089 | 3.070355373 | 0.511277408 | 6.005263142 | 1.91022E-09 |
| IGFBP2 | 27.60762556 | 1.331966768 | 0.222117947 | 5.996664311 | 2.01412E-09 |
| MYLK3 | 3.697907003 | 1.719797077 | 0.287075225 | 5.990754088 | 2.0887E-09 |
| ARHGEF40 | 4.647310349 | 1.636460742 | 0.273184833 | 5.990305991 | 2.09447E-09 |
| SOCS3 | 32.61325003 | 1.141020149 | 0.190688861 | 5.983674888 | 2.18159E-09 |
| H4C4 | 76.52566065 | 1.071319238 | 0.179257881 | 5.976413591 | 2.28103E-09 |
| AQP9 | 12.87131386 | -1.009411456 | 0.169028601 | -5.971838191 | 2.34595E-09 |
| TRAJ30 | 6.091737385 | -1.07300719 | 0.180217553 | -5.953954943 | 2.61739E-09 |
| RN7SL681P | 4.55645903 | 1.393046352 | 0.234026758 | 5.952508872 | 2.64063E-09 |
| SPTBN2 | 5.71585956 | 1.160462759 | 0.195489908 | 5.936177328 | 2.91744E-09 |
| ZDHHC19 | 2.768479239 | 2.258872993 | 0.38097096 | 5.929252431 | 3.04317E-09 |
| CEACAM1 | 1083.879905 | 1.050151627 | 0.177298465 | 5.923072287 | 3.15982E-09 |
| MIR624 | 6.090170914 | 1.545990603 | 0.262847275 | 5.881706791 | 4.06057E-09 |
| PP2D1 | 10.2585799 | 1.005305978 | 0.171081367 | 5.876186255 | 4.19826E-09 |
| MYL12-AS1 | 4.812238646 | 3.460098445 | 0.589174821 | 5.872787367 | 4.28528E-09 |
| CYP1B1-AS1 | 17.71418257 | 1.033869173 | 0.176132386 | 5.869841408 | 4.36212E-09 |
| SNORA31 | 5.665231472 | 1.842519001 | 0.313999864 | 5.867897443 | 4.41356E-09 |
| FAM66B | 15.84243235 | 1.447149612 | 0.246623965 | 5.86783857 | 4.41513E-09 |
| MBOAT2 | 34.06443813 | 1.016891953 | 0.173517002 | 5.860474436 | 4.61547E-09 |
| ROM1 | 6.630512904 | 1.658727151 | 0.283152797 | 5.858063811 | 4.68295E-09 |
| CSGALNACT2 | 5.107999058 | 1.897839026 | 0.324130507 | 5.855169397 | 4.76524E-09 |
| KCNJ15 | 4.918732936 | 1.625508566 | 0.278047358 | 5.846157203 | 5.03059E-09 |
| RN7SL344P | 4.044730647 | 1.995556598 | 0.341848656 | 5.837544072 | 5.29759E-09 |
| SNORA77 | 29.83972521 | 1.738004893 | 0.298184258 | 5.828627263 | 5.58852E-09 |
| CNKSR2 | 2.339071196 | 1.876407976 | 0.323131358 | 5.806951039 | 6.36207E-09 |
| RNASE3 | 25.20595487 | 1.207008967 | 0.207874896 | 5.80642007 | 6.38227E-09 |
| CMKLR2 | 5.658950227 | 1.282156946 | 0.220874154 | 5.804920696 | 6.43965E-09 |
| WDFY3-AS2 | 14.0824851 | -1.165795239 | 0.200873649 | -5.803624542 | 6.48965E-09 |
| MGAM | 86.54879103 | 1.105267494 | 0.190626398 | 5.798082035 | 6.70776E-09 |
| TAS2R18P | 5.821469962 | 1.060689684 | 0.18324408 | 5.788398106 | 7.10608E-09 |
| FOSB | 6.965883671 | 1.769976183 | 0.306510268 | 5.774606495 | 7.71331E-09 |
| RN7SKP134 | 10.77288336 | 1.121636464 | 0.194471452 | 5.767614996 | 8.04012E-09 |
| TRDV3 | 31.89825862 | 1.171063096 | 0.203426556 | 5.75668742 | 8.57805E-09 |
| NECTIN2 | 8.840407356 | -1.153126435 | 0.201109689 | -5.733818405 | 9.81945E-09 |
| XRCC4 | 6.984561245 | 1.025969511 | 0.179052889 | 5.729980227 | 1.00442E-08 |
| IRAG1-AS1 | 14.60999483 | -1.005012116 | 0.175433411 | -5.728738399 | 1.0118E-08 |
| FAM90A1 | 6.928252961 | 1.409918057 | 0.246727337 | 5.714478483 | 1.10041E-08 |
| NTRK3 | 6.202514174 | 1.11111284 | 0.194697098 | 5.706879295 | 1.15066E-08 |
| NLRC4 | 4.885816938 | -1.044989534 | 0.183148391 | -5.705698686 | 1.15867E-08 |
| TRAJ40 | 1.776601588 | -1.374280275 | 0.240931553 | -5.70402779 | 1.17009E-08 |
| WDFY3 | 22.69613999 | 1.052666814 | 0.18471623 | 5.698832262 | 1.20631E-08 |
| SLC1A1 | 10.47491411 | 1.884115397 | 0.330928746 | 5.69341715 | 1.24522E-08 |
| LY6G6C | 6.545977555 | 1.52748137 | 0.268385573 | 5.691369146 | 1.26025E-08 |
| GLB1L2 | 9.236026074 | 1.098768448 | 0.193317333 | 5.683755472 | 1.31769E-08 |
| ERBB3 | 694.6728931 | 1.159072086 | 0.204275179 | 5.674072051 | 1.39442E-08 |
| CKAP2LP1 | 9.667613328 | 1.492930388 | 0.263562017 | 5.664436802 | 1.47508E-08 |
| DDAH2 | 11.84731096 | -1.182760058 | 0.209493353 | -5.645811872 | 1.64404E-08 |
| BASP1-AS1 | 6.937688674 | 1.337401847 | 0.236888922 | 5.645691802 | 1.64518E-08 |
| SCARNA20 | 9.786725637 | 1.077188232 | 0.191341071 | 5.629675985 | 1.80548E-08 |
| RFX2 | 4.915665022 | 1.20238531 | 0.213741867 | 5.625408468 | 1.8507E-08 |
| NDUFB3 | 5.965566606 | 1.194315136 | 0.212347713 | 5.624337163 | 1.86222E-08 |
| TRAJ45 | 4.856144369 | -1.146353935 | 0.20401298 | -5.619024518 | 1.92039E-08 |
| PGLYRP1 | 21.51913161 | -1.007292848 | 0.180257554 | -5.588075634 | 2.296E-08 |
| MYOSLID | 3.48769865 | 1.554837134 | 0.278849129 | 5.575908167 | 2.46242E-08 |
| CLU | 5.520694069 | 1.32391649 | 0.237510547 | 5.574137677 | 2.48759E-08 |
| RNU4-85P | 105.0044281 | 1.070046993 | 0.191966543 | 5.574132736 | 2.48766E-08 |
| GRINA | 16.67140585 | 1.029863278 | 0.185066181 | 5.564837805 | 2.62396E-08 |
| ZNF727 | 10.96784822 | 1.08570463 | 0.195337651 | 5.558091976 | 2.7274E-08 |
| AREG | 11.6324084 | 1.59270352 | 0.286674665 | 5.555787491 | 2.76363E-08 |
| FOXC1 | 2.951269747 | 2.795376577 | 0.50575104 | 5.527179099 | 3.25421E-08 |
| FAM124A | 25.53338961 | 1.042963653 | 0.188974707 | 5.519064806 | 3.40809E-08 |
| non-functional | 11.94781428 | -1.135683394 | 0.205801297 | -5.51834906 | 3.42199E-08 |
| AFAP1L1 | 5.730199641 | 2.219477326 | 0.40267774 | 5.511795429 | 3.55192E-08 |
| SDCBP | 8.72065888 | 1.123570443 | 0.204211075 | 5.502005432 | 3.75495E-08 |
| RNU6-375P | 7.761080752 | 1.101099796 | 0.20027721 | 5.497878653 | 3.84387E-08 |
| MTND3P9 | 1.556701087 | 2.328357879 | 0.423928102 | 5.492341427 | 3.9664E-08 |
| KRTAP5-AS1 | 1.676833505 | 2.775277703 | 0.506459513 | 5.479762209 | 4.25898E-08 |
| RN7SL600P | 9.165971143 | 1.355494621 | 0.247571442 | 5.475165522 | 4.37103E-08 |
| LSMEM1 | 4.350775875 | 1.495641721 | 0.273568322 | 5.467159755 | 4.57304E-08 |
| MTND6P11 | 1.594501489 | 2.921586423 | 0.534902781 | 5.461901732 | 4.71061E-08 |
| FAM151B | 5.449630721 | 1.306026781 | 0.239347833 | 5.45660583 | 4.85322E-08 |
| ALDH1A2 | 4.699940286 | 1.213653988 | 0.222806239 | 5.447127471 | 5.11898E-08 |
| CCPG1 | 13.17661197 | 1.118507207 | 0.206278579 | 5.422313897 | 5.88325E-08 |
| NAIPP1 | 3.314103254 | 1.335685637 | 0.246553689 | 5.417423052 | 6.04642E-08 |
| NAMPT | 901.7013948 | 1.031039074 | 0.190726947 | 5.405838489 | 6.45059E-08 |
| COL1A1 | 34.81289827 | -1.019356437 | 0.188637006 | -5.403798849 | 6.52441E-08 |
| NQO2 | 4.294706459 | 1.26492159 | 0.235299093 | 5.375803097 | 7.62421E-08 |
| TMEM92-AS1 | 3.294297518 | 1.384446894 | 0.258765707 | 5.350194629 | 8.78597E-08 |
| MUC16 | 2.102096476 | 2.041813823 | 0.38180018 | 5.347859768 | 8.90004E-08 |
| MERTK | 4.416553472 | 1.262397459 | 0.236062017 | 5.347736476 | 8.9061E-08 |
| CLEC4A | 11.92016749 | 2.178130862 | 0.407668953 | 5.342891206 | 9.14757E-08 |
| ALOX5AP | 1.924785712 | -1.296484549 | 0.24287823 | -5.338002292 | 9.39763E-08 |
| RNU7-115P | 10.11750602 | -1.259701146 | 0.235999708 | -5.337723333 | 9.41209E-08 |
| SLC8A1 | 21.34415187 | 1.177576315 | 0.220947107 | 5.329675205 | 9.83886E-08 |
| MMP9 | 7.041178197 | -1.132667847 | 0.2126619 | -5.32614374 | 1.0032E-07 |
| PADI4 | 9.558054158 | 1.049411256 | 0.197710442 | 5.307819074 | 1.10945E-07 |
| CCDC17 | 4.511187815 | 1.337940086 | 0.252415137 | 5.300554088 | 1.15452E-07 |
| PPP1R8P1 | 4.565366561 | 1.449556154 | 0.27349213 | 5.300175011 | 1.15692E-07 |
| CFAP97D1 | 1.331784291 | -1.624168339 | 0.307549224 | -5.28100289 | 1.28479E-07 |
| BTNL8 | 8.614382765 | 1.411274208 | 0.267451558 | 5.276746998 | 1.31497E-07 |
| RNU4ATAC11P | 5.718436829 | 2.124460552 | 0.403935962 | 5.25939939 | 1.44527E-07 |
| NXNL2 | 22.08727301 | -1.16838485 | 0.222343788 | -5.254857177 | 1.48139E-07 |
| EDNRB | 2.64369278 | 1.669581444 | 0.318439751 | 5.24300575 | 1.57982E-07 |
| TIMP1 | 44.72468137 | 1.268146222 | 0.241905769 | 5.24231492 | 1.58574E-07 |
| SIAH3 | 2.164761925 | 2.094596478 | 0.400412095 | 5.231101911 | 1.68503E-07 |
| FGD4 | 4.17019424 | 1.384423781 | 0.264702424 | 5.230113727 | 1.69406E-07 |
| EIF1AY | 6.859589786 | 1.319407462 | 0.252900986 | 5.217091013 | 1.81755E-07 |
| MIR1250 | 4.464426787 | 1.401231985 | 0.268671121 | 5.215417188 | 1.83404E-07 |
| SYTL2 | 10.5008385 | 1.0963283 | 0.210573873 | 5.206383314 | 1.92557E-07 |
| RNU6-226P | 2.900831698 | 1.941068469 | 0.373804088 | 5.192742747 | 2.07218E-07 |
| ZNF892 | 5.129821058 | 1.144956057 | 0.220735436 | 5.187006116 | 2.13702E-07 |
| MIR6740 | 4.074589563 | -1.096368514 | 0.21148154 | -5.184227961 | 2.16911E-07 |
| TP53I3 | 2.986003829 | -1.092700828 | 0.210914813 | -5.180768553 | 2.20974E-07 |
| HP | 13.15889097 | 1.216331848 | 0.235044601 | 5.174898065 | 2.28035E-07 |
| GSG1L | 1.419403899 | 2.668330874 | 0.515791537 | 5.173273858 | 2.30027E-07 |
| PEX11G | 3.83598878 | 1.492647082 | 0.288653367 | 5.171071096 | 2.32756E-07 |
| FKBP5 | 3.521286436 | 1.0135065 | 0.196165863 | 5.166579362 | 2.38417E-07 |
| H1-4 | 6.34975717 | -1.171078249 | 0.226674597 | -5.166340921 | 2.38721E-07 |
| NAMPTP1 | 13.95764886 | -1.019530096 | 0.197707872 | -5.15675015 | 2.51273E-07 |
| CD63 | 15.91180193 | 1.211818027 | 0.236022676 | 5.134328813 | 2.83153E-07 |
| KCNE5 | 1.882900401 | -1.543134009 | 0.300778553 | -5.130465569 | 2.89026E-07 |
| CACNA1E | 65.74352938 | 1.367845309 | 0.266816871 | 5.126532298 | 2.95127E-07 |
| RNU6ATAC39P | 17.30354927 | 1.300265664 | 0.253885147 | 5.12147198 | 3.0316E-07 |
| CCN2 | 2.199572807 | 1.924964511 | 0.375987495 | 5.119756734 | 3.0593E-07 |
| SELL | 4.842701828 | 1.047497451 | 0.205631466 | 5.094052339 | 3.5049E-07 |
| AICDA | 100.1549195 | 1.032354603 | 0.203079071 | 5.083510579 | 3.70522E-07 |
| NFIL3 | 3.783533202 | -1.104007423 | 0.217374811 | -5.07881947 | 3.79787E-07 |
| CMBL | 9.748154636 | 1.252198825 | 0.246693103 | 5.075937716 | 3.8559E-07 |
| LIMK2 | 4.651405359 | 2.037641358 | 0.401829691 | 5.070907911 | 3.95922E-07 |
| POR | 4.494918242 | 1.470200783 | 0.291284531 | 5.047301265 | 4.48094E-07 |
| KIF3C | 18.8603035 | 1.167939058 | 0.231557976 | 5.043829961 | 4.56305E-07 |
| MROCKI | 2.613224412 | 1.609903692 | 0.319199155 | 5.043571276 | 4.56922E-07 |
| PRMT5-AS1 | 3.285032079 | 4.436239397 | 0.879933685 | 5.041561056 | 4.61749E-07 |
| ENHO | 5.2934246 | 1.157975864 | 0.229826495 | 5.038478538 | 4.69247E-07 |
| AOC1 | 4.195407247 | 1.800517235 | 0.357665375 | 5.034083145 | 4.80141E-07 |
| RNF149 | 2.578280976 | 1.361187718 | 0.270755993 | 5.027359512 | 4.9728E-07 |
| SLED1 | 36.0204711 | -1.158947207 | 0.230708745 | -5.023421217 | 5.07591E-07 |
| PGA5 | 3.668990672 | 1.454029541 | 0.289480526 | 5.02289242 | 5.08991E-07 |
| G0S2 | 8.433651788 | 1.178277345 | 0.234614769 | 5.022178905 | 5.10886E-07 |
| C1QA | 5.778314553 | 1.459560133 | 0.290860327 | 5.01807911 | 5.21907E-07 |
| H2AC6 | 6.050026622 | 1.298740075 | 0.258990957 | 5.014615519 | 5.31396E-07 |
| NELL2 | 3.887040735 | 1.153122649 | 0.230004554 | 5.013477465 | 5.3455E-07 |
| MCEMP1 | 16.22048329 | 1.000242121 | 0.199651375 | 5.009943562 | 5.4446E-07 |
| FKBP9 | 2.854788307 | -1.219491786 | 0.24346186 | -5.008964376 | 5.47237E-07 |
| MTND5P14 | 3.252642178 | 1.262294173 | 0.252021186 | 5.008682774 | 5.48038E-07 |
| DHRS7 | 4.044112445 | 1.522095851 | 0.304714846 | 4.995148316 | 5.87906E-07 |
| MTCYBP3 | 34.62115426 | 1.271471718 | 0.254862027 | 4.988862923 | 6.07357E-07 |
| DUSP8 | 1.529006234 | 2.996539015 | 0.601385293 | 4.982727461 | 6.26942E-07 |
| ARHGAP29 | 4.300296356 | 1.121367083 | 0.225069658 | 4.982311215 | 6.28293E-07 |
| KLF12 | 3.083816661 | 1.189568691 | 0.238982023 | 4.977649271 | 6.43611E-07 |
| GNG5B | 1.871900957 | -1.202776988 | 0.241881128 | -4.972595409 | 6.60624E-07 |
| KLHL2 | 1.948377548 | 2.484827375 | 0.49981967 | 4.971447752 | 6.64548E-07 |
| SFRP1 | 399.4858877 | -1.032496423 | 0.207936468 | -4.96544177 | 6.85449E-07 |
| APOBEC3B | 13.51646411 | 1.055675402 | 0.212774088 | 4.961484796 | 6.99563E-07 |
| RAB17 | 15.97399265 | 1.162046725 | 0.234271508 | 4.960256309 | 7.04002E-07 |
| RNA5SP498 | 6.462570615 | 1.020078344 | 0.206205013 | 4.946913409 | 7.53995E-07 |
| ATP6V1E1 | 1.123635047 | -1.645233261 | 0.332628154 | -4.946163581 | 7.56904E-07 |
| SHOC1 | 4.571827351 | 1.167044737 | 0.236278758 | 4.93927067 | 7.84153E-07 |
| ALB | 19.95288443 | 1.004164613 | 0.203511559 | 4.934189566 | 8.04842E-07 |
| ANKRD34B | 2.268902655 | 4.081729548 | 0.827362349 | 4.933424337 | 8.08004E-07 |
| DSC1 | 18.96104118 | 1.031992952 | 0.20930276 | 4.930622766 | 8.19679E-07 |
| POMP | 1.405665816 | -1.430455638 | 0.290444885 | -4.925050206 | 8.43387E-07 |
| RNU6-574P | 1.792062099 | 2.015769642 | 0.40962244 | 4.921043001 | 8.60842E-07 |
| CFAP97D2 | 5.487650521 | 1.275877906 | 0.259291397 | 4.920633385 | 8.62646E-07 |
| RPL21P123 | 215.6872533 | 1.135803871 | 0.230932728 | 4.918332197 | 8.72847E-07 |
| LHFPL6 | 13.14444614 | 1.084228344 | 0.220693849 | 4.912816326 | 8.97774E-07 |
| ASPH | 2.110283499 | 1.636340773 | 0.333078617 | 4.912776413 | 8.97957E-07 |
| HPN | 3.914836412 | 1.695759812 | 0.345364915 | 4.910052354 | 9.10521E-07 |
| GPR37L1 | 40.35773785 | -1.108829636 | 0.226149543 | -4.903081489 | 9.43448E-07 |
| SLA | 15.51174705 | 1.073076144 | 0.219360786 | 4.891832139 | 9.99016E-07 |
| RN7SL105P | 2.765781744 | 1.417835508 | 0.289968642 | 4.889616679 | 1.01033E-06 |
| ANKRD35 | 2.653260377 | 1.76838267 | 0.361725946 | 4.888736038 | 1.01485E-06 |
| EZR-AS1 | 6.083486407 | 1.107715108 | 0.226774081 | 4.884663644 | 1.03606E-06 |
| SCN2B | 5.935753514 | 1.556577257 | 0.319159321 | 4.877116715 | 1.07648E-06 |
| RN7SL502P | 3.614187061 | 1.289869166 | 0.265624431 | 4.855988441 | 1.19788E-06 |
| IGKV1-27 | 3.953479825 | 1.119445246 | 0.230734434 | 4.851660967 | 1.22432E-06 |
| MIR3155A | 3.697723078 | 1.091408131 | 0.225198999 | 4.846416434 | 1.25712E-06 |
| C3orf86P | 1.62707891 | 1.828373546 | 0.377365528 | 4.845099541 | 1.26548E-06 |
| LHX1 | 5.813182925 | 1.43845 | 0.297136 | 4.841049223 | 1.29155E-06 |
| CASP4 | 2.94296952 | 1.358225128 | 0.280750357 | 4.837839361 | 1.31258E-06 |
| Knops blood group | 2.50378617 | 1.229766417 | 0.25424709 | 4.836894751 | 1.31883E-06 |
| SNORA70B | 3.103296568 | 1.571856154 | 0.325100797 | 4.834980932 | 1.33158E-06 |
| MAP2K6 | 5.247649974 | -1.096983543 | 0.227621809 | -4.819325311 | 1.44045E-06 |
| PLEKHS1 | 17.69477707 | -1.018121636 | 0.211263542 | -4.819201763 | 1.44134E-06 |
| RSPH14 | 2.072376491 | 3.079209583 | 0.639228347 | 4.817072954 | 1.45679E-06 |
| GCA | 12.26704443 | 1.305429068 | 0.271030767 | 4.816534607 | 1.46073E-06 |
| PLIN4 | 3.013330439 | 1.172866264 | 0.243563533 | 4.8154428 | 1.46874E-06 |
| CDO1 | 0.92575851 | 1.942678894 | 0.404438169 | 4.803401464 | 1.55993E-06 |
| CBS | 2.988297962 | 2.005651452 | 0.417843941 | 4.800001279 | 1.58665E-06 |
| CD300LD | 0.984559432 | 2.099269858 | 0.438206476 | 4.790595235 | 1.66287E-06 |
| MXRA8 | 2.529812132 | 1.699316579 | 0.355835092 | 4.775573334 | 1.79196E-06 |
| HK3 | 115.9995599 | 1.682968883 | 0.352542958 | 4.773798043 | 1.80784E-06 |
| RNASE2 | 3.037506041 | 1.215036954 | 0.254892978 | 4.766851424 | 1.87127E-06 |
| AGFG1 | 3.849978633 | 1.851144483 | 0.39041467 | 4.741482904 | 2.1216E-06 |
| ADAMTS2 | 9.758128458 | 1.33203426 | 0.280987801 | 4.740541245 | 2.13148E-06 |
| RN7SL634P | 3.779994158 | 1.677857572 | 0.354003654 | 4.739661729 | 2.14075E-06 |
| ELANE | 14.93537619 | 1.628139418 | 0.34366123 | 4.737629025 | 2.16233E-06 |
| SF3B6 | 3.738861642 | 1.361713061 | 0.287723703 | 4.732710748 | 2.21541E-06 |
| NOS1AP | 14.95363866 | 1.03254902 | 0.218696746 | 4.721373499 | 2.34257E-06 |
| ASGR2 | 1.544376022 | 2.256877787 | 0.479477375 | 4.706953664 | 2.51446E-06 |
| ARHGAP39 | 2.442973273 | 2.244064109 | 0.477286676 | 4.701711195 | 2.5799E-06 |
| EDIL3 | 2.419181015 | 1.373497469 | 0.292568711 | 4.694615041 | 2.67109E-06 |
| CCDC39 | 3.428684968 | 1.228566329 | 0.261803258 | 4.692708321 | 2.69612E-06 |
| APOBEC3A | 3.346881365 | 1.058134297 | 0.225794541 | 4.686270507 | 2.78228E-06 |
| DOCK4 | 10.62581921 | 1.032858655 | 0.220405279 | 4.686179286 | 2.78352E-06 |
| WFDC1 | 55.87262197 | 1.156287475 | 0.246748824 | 4.686091123 | 2.78472E-06 |
| RNA5SP207 | 7.173114401 | 2.39378573 | 0.510934706 | 4.685110841 | 2.79808E-06 |
| OR2C1 | 2.872484414 | 1.066678107 | 0.227831087 | 4.681881295 | 2.84254E-06 |
| HSD11B1-AS1 | 14.69468486 | 1.447509523 | 0.309452679 | 4.677644183 | 2.9019E-06 |
| TRAJ14 | 3.206098651 | 1.428751573 | 0.305847976 | 4.671443618 | 2.9909E-06 |
| SLC22A4 | 1.843691223 | 1.736299025 | 0.372054459 | 4.666787302 | 3.05946E-06 |
| CFAP58-DT | 3.609309337 | 1.203228478 | 0.257857099 | 4.666260813 | 3.0673E-06 |
| BEX1 | 2.052934661 | -1.117476158 | 0.2399533 | -4.657056853 | 3.20762E-06 |
| RN7SL760P | 2.931705094 | 1.504311852 | 0.323223467 | 4.654092312 | 3.25411E-06 |
| NRCAM | 1.304288309 | 2.586788824 | 0.558328422 | 4.633095369 | 3.60238E-06 |
| PROK2 | 3.445034063 | 1.194133652 | 0.257777225 | 4.632424963 | 3.61407E-06 |
| C2 | 3.788228344 | 1.309167705 | 0.282726099 | 4.630515921 | 3.64756E-06 |
| HSD3B7 | 3.466061607 | 1.608331763 | 0.348223677 | 4.618674347 | 3.86199E-06 |
| PLPP3 | 3.762773361 | 1.036648183 | 0.224503314 | 4.61751839 | 3.88356E-06 |
| LCN2 | 2.572243642 | 1.831220225 | 0.397071236 | 4.61181788 | 3.99163E-06 |
| LYVE1 | 2.41100473 | 1.628285011 | 0.353456249 | 4.606751228 | 4.09009E-06 |
| TRAJ39 | 4.431427204 | 1.378091865 | 0.299431097 | 4.602367213 | 4.17716E-06 |
| PPL | 1.523210106 | -1.090528563 | 0.237025945 | -4.600882681 | 4.20704E-06 |
| NFASC | 2.083789264 | 1.303971154 | 0.283611365 | 4.597739425 | 4.271E-06 |
| PPP1R3D | 1.048346185 | -1.44628178 | 0.315176477 | -4.588799876 | 4.45802E-06 |
| TRAJ10 | 25.27636298 | 1.171061896 | 0.255224084 | 4.588367507 | 4.46726E-06 |
| PGD | 3.854805086 | 1.150184313 | 0.250875459 | 4.584682435 | 4.54677E-06 |
| SH3GLB1 | 1.265213096 | -1.472264033 | 0.322234508 | -4.568921073 | 4.90241E-06 |
| GPC1 | 4.572501807 | -1.226450307 | 0.268824393 | -4.562273134 | 5.06028E-06 |
| INPP4B | 4.445521778 | 1.11509824 | 0.244771311 | 4.555673767 | 5.22179E-06 |
| ARMC12 | 492.0706832 | 1.029179722 | 0.226789575 | 4.538038058 | 5.678E-06 |
| TMEM45A | 2.678680512 | 1.181472148 | 0.261481619 | 4.518375533 | 6.23159E-06 |
| HCG14 | 9.415713786 | 1.601845304 | 0.354849139 | 4.514158634 | 6.35686E-06 |
| WIPI1 | 2.69129966 | 1.263512329 | 0.27995242 | 4.513310981 | 6.38233E-06 |
| NME8 | 1.647247946 | 1.500541886 | 0.332796152 | 4.508891943 | 6.51671E-06 |
| RN7SL587P | 2.411066203 | 1.380880215 | 0.306672924 | 4.502778386 | 6.70708E-06 |
| RALB | 5.604201101 | 1.188764523 | 0.264495827 | 4.494454729 | 6.97484E-06 |
| SHROOM2 | 863.2097058 | -1.055020188 | 0.234851783 | -4.492280935 | 7.04644E-06 |
| PSTPIP2 | 3.797674854 | 1.196370352 | 0.266416971 | 4.490593633 | 7.10249E-06 |
| ST6GALNAC3 | 2.622254121 | 1.383703549 | 0.308151709 | 4.490332219 | 7.11122E-06 |
| MIR3150BHG | 1.275568528 | 2.023030516 | 0.450857415 | 4.48707385 | 7.22081E-06 |
| PDSS1 | 1.758749287 | 1.44414217 | 0.322206113 | 4.482044596 | 7.39313E-06 |
| KCNE1 | 3.538867117 | 1.121076884 | 0.250542147 | 4.474603969 | 7.65531E-06 |
| ANOS1 | 4.893675948 | 1.043022629 | 0.23336389 | 4.46951167 | 7.83984E-06 |
| P2RY13 | 2.907078527 | 1.089469237 | 0.244053746 | 4.464054562 | 8.04231E-06 |
| ANXA9 | 2.57166335 | 1.306662045 | 0.29279222 | 4.462762173 | 8.09098E-06 |
| MIR223HG | 4.281490815 | 1.011049214 | 0.226669284 | 4.460459735 | 8.1784E-06 |
| MAOB | 1.758195135 | 1.638826563 | 0.367429355 | 4.460249412 | 8.18643E-06 |
| KLRC4 | 2.08941177 | -1.160909408 | 0.260553224 | -4.455555721 | 8.36762E-06 |
| ACOX2 | 3.257296451 | 1.065274313 | 0.239181882 | 4.453825285 | 8.43538E-06 |
| MTND5P28 | 1.017582912 | 2.571284947 | 0.577471777 | 4.452659074 | 8.48134E-06 |
| RPL21P99 | 8.852137357 | 1.117636437 | 0.251240664 | 4.448469518 | 8.64843E-06 |
| CLEC1B | 6.346050546 | 1.298035789 | 0.292208845 | 4.442150918 | 8.90641E-06 |
| ZNF516-DT | 66.50401669 | 1.124780987 | 0.253290119 | 4.440682456 | 8.9674E-06 |
| NKX6-2 | 5.716970942 | -1.026981212 | 0.231271776 | -4.44058169 | 8.9716E-06 |
| XIRP2 | 2.54460622 | 1.479304101 | 0.333293478 | 4.438442995 | 9.0612E-06 |
| RNA5SP180 | 2.86999281 | 1.247542831 | 0.281624777 | 4.429804958 | 9.43184E-06 |
| PDGFC | 3.056334451 | 1.446409796 | 0.326760224 | 4.426517331 | 9.57667E-06 |
| FARP1 | 3.811053858 | 1.256456277 | 0.283926882 | 4.425281147 | 9.63168E-06 |
| FLVCR2-AS1 | 2.20403108 | 1.287953727 | 0.291145927 | 4.423739457 | 9.7007E-06 |
| UMODL1-AS1 | 0.853470223 | -1.508447507 | 0.341096487 | -4.422348403 | 9.76339E-06 |
| PPARG | 0.873752301 | -1.435449813 | 0.324794893 | -4.419557831 | 9.89031E-06 |
| AIF1 | 2.910103538 | 1.67875095 | 0.380019929 | 4.417533985 | 9.98334E-06 |
| CRISP3 | 1.866445996 | -1.661590312 | 0.377122772 | -4.40596653 | 1.05313E-05 |
| MAOA | 5.806052796 | 1.425967775 | 0.323681613 | 4.405464256 | 1.05558E-05 |
| TRBJ2-1 | 1.617583494 | 2.193974284 | 0.498297229 | 4.402942976 | 1.06792E-05 |
| OLAH | 2.31104249 | 1.175706166 | 0.267347935 | 4.397663166 | 1.09423E-05 |
| TRAJ34 | 2.941960324 | 1.582760323 | 0.360139186 | 4.39485728 | 1.10846E-05 |
| BPI | 5.051164456 | 1.402658108 | 0.31990729 | 4.384576886 | 1.16211E-05 |
| CARD16 | 2.236735893 | 1.388659202 | 0.316950933 | 4.381306561 | 1.1797E-05 |
| TENM1 | 3.028171332 | 2.077616767 | 0.474815426 | 4.375630301 | 1.21082E-05 |
| CD248 | 3.035727709 | 1.331527321 | 0.304325775 | 4.375335353 | 1.21246E-05 |
| F12 | 1.131600482 | -1.410581049 | 0.323257087 | -4.363650806 | 1.2791E-05 |
| NRN1 | 5.271819529 | -1.116178325 | 0.255944368 | -4.361019286 | 1.29458E-05 |
| C12orf40 | 4.838470003 | 1.100415483 | 0.25249703 | 4.358132388 | 1.31177E-05 |
| IRS2 | 5.05632841 | 1.21811634 | 0.279782398 | 4.353799048 | 1.33798E-05 |
| MMADHC | 1.910508484 | 1.20263219 | 0.276291131 | 4.352771607 | 1.34427E-05 |
| HPGD | 2.913387454 | 2.293097995 | 0.527852654 | 4.344200935 | 1.39784E-05 |
| NDUFB4P11 | 9.649117084 | 1.002884903 | 0.231030267 | 4.340924318 | 1.41885E-05 |
| KLHL29 | 2.305582719 | 1.306078628 | 0.301806982 | 4.32752954 | 1.50791E-05 |
| MKNK1 | 7.309102604 | 1.009259267 | 0.233577726 | 4.320871183 | 1.55414E-05 |
| ACER3 | 4.493646166 | 1.11925902 | 0.259561121 | 4.312121228 | 1.61696E-05 |
| TAB2-AS1 | 1.693349479 | -1.072768899 | 0.248921211 | -4.309672502 | 1.63496E-05 |
| LIPN | 1.681931035 | 1.519105327 | 0.35311818 | 4.301974281 | 1.69283E-05 |
| GYG1 | 6.474571182 | 1.091215113 | 0.253824365 | 4.299095216 | 1.71497E-05 |
| MIR765 | 1.220186457 | 2.556171189 | 0.594825275 | 4.297347971 | 1.72854E-05 |
| MIR1262 | 4.312977802 | 1.365273672 | 0.318489 | 4.286721587 | 1.81329E-05 |
| RNU6-176P | 5.794161433 | 1.057840218 | 0.246856926 | 4.285236123 | 1.82545E-05 |
| PRL | 0.945809092 | 2.105101117 | 0.491577824 | 4.2823354 | 1.84942E-05 |
| TMTC1 | 277.3290057 | -1.268050285 | 0.297217853 | -4.266400124 | 1.98652E-05 |
| CDH6 | 0.812680201 | 1.90225498 | 0.446200902 | 4.263225314 | 2.01497E-05 |
| STAG2-AS1 | 1.31907016 | -1.240352138 | 0.29121474 | -4.259235421 | 2.05127E-05 |
| NCF2 | 5.873102918 | 1.023938829 | 0.240854746 | 4.25127114 | 2.12561E-05 |
| LILRA5 | 1.260205672 | 3.462651069 | 0.814603638 | 4.250718888 | 2.13085E-05 |
| PRSS35 | 1.113285301 | -1.284837188 | 0.302387058 | -4.248982073 | 2.14744E-05 |
| PROKR2 | 31.80928997 | -1.197054596 | 0.281935899 | -4.245839566 | 2.17776E-05 |
| RPL23AP81 | 1.536017279 | 1.438887306 | 0.338979725 | 4.244759197 | 2.18828E-05 |
| KL | 0.920793333 | -1.406197414 | 0.331619825 | -4.240390067 | 2.23132E-05 |
| SLC2A14 | 4.597065762 | -1.133021491 | 0.267563787 | -4.234584593 | 2.28975E-05 |
| MAMDC2 | 5.254616701 | 1.034426958 | 0.244331405 | 4.233704458 | 2.29873E-05 |
| S100A12 | 2.716584227 | 1.309314315 | 0.309335898 | 4.232662051 | 2.30941E-05 |
| FDPSP3 | 1.377980706 | 1.543442266 | 0.365217334 | 4.226092587 | 2.37784E-05 |
| SEMA4A | 2.751835064 | 1.258720149 | 0.298238525 | 4.220514935 | 2.43745E-05 |
| SMOC1 | 1.054877559 | 1.649669336 | 0.391660677 | 4.211986121 | 2.53135E-05 |
| RNU6-1003P | 1.252649967 | 1.701352448 | 0.403987303 | 4.21140079 | 2.53792E-05 |
| SLC44A3 | 2.098269772 | 1.372509759 | 0.32620083 | 4.207560599 | 2.58142E-05 |
| OLFM4 | 14.15875191 | 1.521853492 | 0.361821209 | 4.206092549 | 2.59824E-05 |
| ACTA2-AS1 | 3.767533765 | 1.199709364 | 0.285347301 | 4.204383084 | 2.61795E-05 |
| CPE | 1.255022147 | -1.36135651 | 0.324239153 | -4.198618509 | 2.68548E-05 |
| OAT | 11.0534818 | -1.070908715 | 0.255116611 | -4.197722418 | 2.69613E-05 |
| S100A2 | 1.917911162 | 3.415761105 | 0.814221678 | 4.195124248 | 2.72722E-05 |
| FLOT2 | 1.634396084 | -1.189918623 | 0.283853635 | -4.192014752 | 2.76488E-05 |
| C19orf38 | 1.30221548 | 1.650297573 | 0.393776474 | 4.190950154 | 2.77789E-05 |
| XIST | 1.22901296 | 1.671417798 | 0.398956591 | 4.189472827 | 2.79603E-05 |
| MROH6 | 2.293120835 | 1.448360129 | 0.345790478 | 4.188548326 | 2.80745E-05 |
| GPR17 | 1.655480474 | 1.423737248 | 0.340268255 | 4.184161254 | 2.86221E-05 |
| ITGAM | 2.636788214 | 1.139703398 | 0.272806144 | 4.177704293 | 2.94466E-05 |
| CYB5R2 | 5.700635192 | 1.397756362 | 0.336021519 | 4.15972276 | 3.18634E-05 |
| TMLHE-AS1 | 3.561154397 | 1.198512165 | 0.288222035 | 4.158294716 | 3.20632E-05 |
| HNRNPMP1 | 4.341855313 | 1.144015678 | 0.275252541 | 4.1562402 | 3.23528E-05 |
| RPS20P29 | 2.354272152 | -1.244718949 | 0.300664457 | -4.139893892 | 3.47467E-05 |
| AKR1C1 | 19.15994073 | 1.201477519 | 0.291631515 | 4.119848014 | 3.79122E-05 |
| CCNA1 | 1.873198499 | -1.036061792 | 0.251617012 | -4.117614234 | 3.82815E-05 |
| CLEC5A | 3.178781499 | 2.493462512 | 0.605876012 | 4.115466636 | 3.86397E-05 |
| CLIC1 | 0.92953669 | -1.517889897 | 0.370398769 | -4.097988501 | 4.16756E-05 |
| KLRC4-KLRK1 | 3.123398551 | 1.155987189 | 0.282261709 | 4.095444584 | 4.21359E-05 |
| RGL4 | 13.6311643 | 1.059154615 | 0.258822311 | 4.092207551 | 4.27286E-05 |
| SLC16A10 | 2.519980026 | 1.393279759 | 0.341714911 | 4.077316253 | 4.55585E-05 |
| HPD | 3.268110687 | 1.007030785 | 0.247250525 | 4.072916666 | 4.6428E-05 |
| INSC | 5.3159865 | 1.003424774 | 0.24714528 | 4.060060445 | 4.906E-05 |
| PFKFB3 | 1.778717041 | 1.728440662 | 0.425750408 | 4.059751038 | 4.91251E-05 |
| NDUFAF2P1 | 0.809992429 | 2.003942314 | 0.495102111 | 4.04753337 | 5.17602E-05 |
| FSBP | 2.461066093 | 2.219615101 | 0.548448353 | 4.047081353 | 5.18602E-05 |
| OSBPL6 | 5.028636106 | 1.029726558 | 0.254821455 | 4.040972757 | 5.32299E-05 |
| RTN3 | 2.488838121 | -1.02178704 | 0.253141716 | -4.036422973 | 5.42723E-05 |
| INHBB | 2.231902214 | 1.234462785 | 0.306475426 | 4.027933982 | 5.62691E-05 |
| RNU7-45P | 1.417319109 | 1.305921876 | 0.324655162 | 4.022489183 | 5.75863E-05 |
| ZNF608 | 2.100158696 | 1.333604819 | 0.332402264 | 4.012020865 | 6.02012E-05 |
| PRDM5 | 2.832731227 | 1.270792862 | 0.317114216 | 4.007366427 | 6.13996E-05 |
| RPL7P51 | 2.34462268 | 1.014529948 | 0.253202018 | 4.006800395 | 6.15468E-05 |
| MIR7161 | 2.836910664 | 1.00710944 | 0.25170081 | 4.001216528 | 6.30177E-05 |
| CHIT1 | 1.419986587 | -1.311213751 | 0.328964166 | -3.985886258 | 6.72287E-05 |
| CARD6 | 0.924551118 | -1.299236851 | 0.326477942 | -3.979554768 | 6.90445E-05 |
| CIR1P2 | 1.464732325 | -1.170697543 | 0.294205272 | -3.979186146 | 6.91516E-05 |
| RALBP1P1 | 0.49200833 | 2.148581206 | 0.540141182 | 3.977814091 | 6.95517E-05 |
| ABHD12B | 1.676343808 | -1.255585633 | 0.31592608 | -3.974301936 | 7.0586E-05 |
| SAT1 | 1.990717437 | -1.104231441 | 0.277888902 | -3.973643544 | 7.07815E-05 |
| CSTA | 2.450851258 | 1.125678944 | 0.283446855 | 3.971393305 | 7.14535E-05 |
| RPSAP36 | 1.857310624 | 1.256183316 | 0.31717193 | 3.960575317 | 7.47694E-05 |
| FCGR2A | 1.863971063 | 1.766153804 | 0.446028699 | 3.959731305 | 7.50341E-05 |
| TEAD2 | 1.492208407 | 1.978142476 | 0.499829293 | 3.957636141 | 7.56951E-05 |
| PIERCE2 | 0.923919185 | 1.836687165 | 0.464402714 | 3.954944942 | 7.65523E-05 |
| SLC12A9 | 1.196747233 | -1.38305479 | 0.350420498 | -3.946843292 | 7.91883E-05 |
| ARG1 | 3.061391378 | 1.425370416 | 0.361169372 | 3.946542886 | 7.92877E-05 |
| RAB19 | 22.92305187 | 1.340928278 | 0.339869501 | 3.945421038 | 7.96599E-05 |
| TRAJ7 | 1.212340675 | 1.526084599 | 0.386832908 | 3.945074392 | 7.97752E-05 |
| HCG9 | 1.962053923 | 1.196681013 | 0.303807957 | 3.938939013 | 8.18427E-05 |
| RNU6-936P | 1.183969212 | 1.74472497 | 0.443794755 | 3.931378075 | 8.44603E-05 |
| RPL14P4 | 1.680783045 | 1.333691557 | 0.339460983 | 3.928850812 | 8.53528E-05 |
| RSAD2 | 1.419870282 | 1.301006324 | 0.331361426 | 3.926245552 | 8.62821E-05 |
| OTX1 | 15.17593101 | 1.094936512 | 0.279028374 | 3.924104552 | 8.70529E-05 |
| KRT5 | 1.034834986 | 2.122634284 | 0.541892581 | 3.917075741 | 8.96296E-05 |
| ARL11 | 0.742173481 | 2.06301555 | 0.52703171 | 3.91440498 | 9.06274E-05 |
| LILRB5 | 0.675242925 | -1.538128841 | 0.393037197 | -3.913443438 | 9.09892E-05 |
| MAP1B | 1.742888893 | -1.044052495 | 0.266793028 | -3.913342503 | 9.10273E-05 |
| LY96 | 1.246918049 | 1.381916796 | 0.353186859 | 3.912707284 | 9.12671E-05 |
| TMEM150C | 2.830311355 | 1.018476251 | 0.261795206 | 3.890354851 | 0.000100098 |
| MSRB1 | 0.815292325 | 1.746691586 | 0.449983074 | 3.881682861 | 0.000103736 |
| GSTO1 | 2.376669848 | 1.389319913 | 0.358100593 | 3.879691738 | 0.000104589 |
| OR2W6P | 1.153016713 | 2.349540916 | 0.605663738 | 3.87928279 | 0.000104765 |
| BCL2A1 | 0.728680457 | -1.512088022 | 0.389791454 | -3.879223123 | 0.000104791 |
| RNU6-790P | 2.317005199 | 1.174620808 | 0.303393594 | 3.871607146 | 0.00010812 |
| SERPINA1 | 1.127307891 | 1.907942383 | 0.493265711 | 3.867980969 | 0.00010974 |
| MMP19 | 3.547306275 | -1.285716981 | 0.332461304 | -3.867268054 | 0.000110061 |
| CYSTM1 | 1.451266143 | 1.244741539 | 0.32188502 | 3.867037796 | 0.000110165 |
| VNN1 | 1.030865442 | 1.396489616 | 0.361276907 | 3.86542729 | 0.000110895 |
| MTND2P28 | 2.526734912 | 1.239716564 | 0.320728998 | 3.865308628 | 0.000110949 |
| RN7SL388P | 2.202871839 | 1.138605606 | 0.29474009 | 3.863083589 | 0.000111965 |
| SPAG6 | 1.549490637 | 1.360070525 | 0.352166351 | 3.862011577 | 0.000112457 |
| KCTD19 | 1.310934556 | -1.139301634 | 0.295177701 | -3.859714432 | 0.00011352 |
| SERPINB10 | 1.406621727 | 1.669924675 | 0.432961317 | 3.856983542 | 0.000114795 |
| OR52B4 | 0.692257918 | -1.631731823 | 0.423504727 | -3.852924697 | 0.000116715 |
| CAMKK2 | 1.709466436 | 2.085242215 | 0.541714264 | 3.849339688 | 0.000118437 |
| RPS10P1 | 1.486951885 | 1.76955803 | 0.459883104 | 3.847843101 | 0.000119162 |
| COL5A1 | 0.921348857 | -1.279057315 | 0.332532501 | -3.84641294 | 0.00011986 |
| PLIN5 | 1.604917063 | 1.381973021 | 0.359305609 | 3.846232805 | 0.000119948 |
| MAPK14 | 3.383973994 | 1.458095351 | 0.379119443 | 3.846005205 | 0.000120059 |
| UVRAG-DT | 1.536593218 | 1.21101196 | 0.315197776 | 3.842070126 | 0.000122001 |
| NCR3LG1 | 2.299287637 | 1.054689218 | 0.275030204 | 3.834812335 | 0.00012566 |
| B4GALT5 | 2.119778147 | 1.118323631 | 0.291867945 | 3.831608271 | 0.000127308 |
| CYP1B1 | 1.443374382 | -1.13118233 | 0.295268737 | -3.83102641 | 0.00012761 |
| TCN2 | 0.960784227 | 1.729157025 | 0.451952307 | 3.825972336 | 0.000130257 |
| PKD1L3 | 2.459964617 | 1.095393143 | 0.287841322 | 3.805545133 | 0.000141492 |
| TRGJ2 | 2.077653992 | 1.219484653 | 0.321074166 | 3.798140066 | 0.000145786 |
| TNFAIP8L3 | 1.347911584 | 1.737723678 | 0.457609477 | 3.797394426 | 0.000146225 |
| SDHAF3 | 2.213395541 | 1.043357743 | 0.275226195 | 3.790910025 | 0.000150096 |
| SCGB1C1 | 2.271188282 | 1.101100232 | 0.2909896 | 3.783984826 | 0.000154337 |
| KNOP1P2 | 6.13648287 | -1.157172287 | 0.305965583 | -3.782034166 | 0.000155552 |
| GRB10 | 3.055302233 | -1.090046929 | 0.288221191 | -3.781980513 | 0.000155586 |
| RPL23P8 | 1.960722471 | -1.548328346 | 0.410201432 | -3.77455617 | 0.000160293 |
| RPS3AP43 | 0.79613662 | 2.419392238 | 0.640993107 | 3.774443455 | 0.000160365 |
| FSTL4 | 8.629158136 | 1.194057256 | 0.316416707 | 3.77368587 | 0.000160853 |
| TTTY10 | 1.900442182 | 1.577532275 | 0.418151274 | 3.77263534 | 0.000161532 |
| USP6 | 1.852103971 | 1.111215225 | 0.294614907 | 3.771754924 | 0.000162103 |
| EHF | 1.724008476 | 1.046681974 | 0.279302448 | 3.747485862 | 0.000178616 |
| SAG | 0.855567698 | -1.210040492 | 0.323046115 | -3.745720615 | 0.000179877 |
| CXCR3 | 2.358584443 | 1.335129144 | 0.356586817 | 3.744190974 | 0.000180976 |
| DUXAP1 | 1.296649133 | 1.473105173 | 0.393517837 | 3.743426689 | 0.000181528 |
| HM13-AS1 | 0.923219746 | 1.528522975 | 0.408921773 | 3.737934923 | 0.000185538 |
| VCAN-AS1 | 0.792793903 | 1.866915113 | 0.499460886 | 3.737860491 | 0.000185593 |
| ASAP1-IT2 | 1.385267938 | -1.171660371 | 0.313845468 | -3.733239738 | 0.000189033 |
| FPR1 | 6.094565289 | -1.153434415 | 0.309207181 | -3.730296336 | 0.000191255 |
| MMP25 | 2.822878878 | -1.184607722 | 0.317849907 | -3.726940595 | 0.000193818 |
| ADM | 1.005890371 | -1.362385687 | 0.365699028 | -3.725428786 | 0.000194983 |
| GLT1D1 | 2.398809617 | 1.566655899 | 0.420648705 | 3.724380652 | 0.000195795 |
| RPS15AP18 | 0.708372638 | 1.735237264 | 0.466161455 | 3.722395414 | 0.000197342 |
| STXBP2 | 0.943500704 | -1.248376704 | 0.335876374 | -3.716774388 | 0.000201782 |
| BIK | 1.833951352 | -1.293583017 | 0.348202249 | -3.715033495 | 0.000203177 |
| IFI44L | 2.233062013 | 1.309982033 | 0.35285514 | 3.712520764 | 0.000205205 |
| TRIM72 | 5.776547018 | 1.055762848 | 0.284893861 | 3.705811162 | 0.000210715 |
| C3AR1 | 1.663486088 | 1.064342383 | 0.287348317 | 3.704014673 | 0.000212214 |
| THBS3-AS1 | 1.839457704 | 1.07690107 | 0.291031717 | 3.700287662 | 0.000215355 |
| CASC8 | 1.67790041 | 1.115394259 | 0.302128332 | 3.691789677 | 0.000222682 |
| NAIP | 1.464162272 | 1.332413272 | 0.360989351 | 3.691004369 | 0.00022337 |
| PDCD10 | 1.037428731 | 2.095302794 | 0.567906143 | 3.68952303 | 0.000224675 |
| PCP4L1 | 1.109428984 | 1.770802461 | 0.480958111 | 3.681822637 | 0.000231572 |
| CKLF | 0.882027212 | 1.532617388 | 0.416400992 | 3.680628574 | 0.00023266 |
| MIR3605 | 0.847693868 | -1.301546706 | 0.353663061 | -3.680188435 | 0.000233062 |
| MYO16-AS1 | 2.180036153 | 1.118922085 | 0.304727084 | 3.671882628 | 0.00024077 |
| PGS1 | 22.22389057 | 1.262297363 | 0.343941866 | 3.670089305 | 0.000242466 |
| SLC28A3 | 1.236942839 | 1.439765516 | 0.392464929 | 3.668520189 | 0.000243958 |
| NT5DC4 | 0.677042878 | 1.870080505 | 0.510151198 | 3.665737754 | 0.000246627 |
| CCR12P | 1.963777919 | 1.126038066 | 0.307588091 | 3.660863659 | 0.000251367 |
| SEPT5-GP1BB | 1.530975596 | 1.710749722 | 0.467409657 | 3.660064989 | 0.000252151 |
| ZFAS1 | 1.546689903 | 1.287896803 | 0.352044107 | 3.658339328 | 0.000253855 |
| FAM83A | 0.852096336 | 2.07304655 | 0.567136404 | 3.6552874 | 0.000256894 |
| MAK | 0.777315179 | -1.352238366 | 0.370866106 | -3.646163254 | 0.000266185 |
| UPP1 | 1.435197182 | 1.571968943 | 0.431308016 | 3.644655059 | 0.000267751 |
| PXN-AS1 | 0.64032735 | 1.563606052 | 0.429150375 | 3.643492222 | 0.000268964 |
| ARHGAP20 | 31.63559675 | 1.661097439 | 0.456645839 | 3.63760555 | 0.000275184 |
| RPS4Y2 | 2.13667236 | 1.276094716 | 0.351211799 | 3.633405024 | 0.000279705 |
| RNU6-672P | 1.423831079 | 1.222082624 | 0.33684778 | 3.627996672 | 0.000285629 |
| RN7SL491P | 3.959716656 | 1.130335841 | 0.313548418 | 3.604980205 | 0.000312177 |
| RNU7-181P | 2.14635868 | -1.045866549 | 0.290990831 | -3.594156374 | 0.000325444 |
| K7EIL1_HUMAN | 0.707371312 | 1.861287518 | 0.51996393 | 3.579647378 | 0.000344058 |
| CAPN13 | 1.195029527 | 1.419687487 | 0.396874461 | 3.577170179 | 0.000347334 |
| IL6 | 4.16509763 | -1.016928687 | 0.284505587 | -3.574371589 | 0.00035107 |
| CHMP4C | 1.467448266 | 1.364399225 | 0.38172865 | 3.574264663 | 0.000351214 |
| MNDA | 0.605531113 | 2.041625458 | 0.571382591 | 3.573132064 | 0.000352737 |
| GPR141BP | 0.904610991 | 2.374725211 | 0.665981466 | 3.565752699 | 0.000362813 |
| NXPH4 | 0.931046992 | 1.517923866 | 0.426609914 | 3.558107337 | 0.000373537 |
| NDUFA1 | 1.076486444 | 1.5127888 | 0.427210525 | 3.541085045 | 0.000398485 |
| EMILIN2 | 1.009624863 | -1.219391542 | 0.344997626 | -3.534492558 | 0.000408559 |
| RNU5A-1 | 1.539873034 | -1.041614076 | 0.294922709 | -3.531820523 | 0.000412709 |
| TNFSF10 | 1.084533224 | 1.437045498 | 0.407637318 | 3.525304077 | 0.000422997 |
| IKZF2 | 2.513410912 | 1.168137367 | 0.331419728 | 3.524646449 | 0.000424048 |
| BASP1 | 6.859812337 | -1.081258482 | 0.308067406 | -3.509811363 | 0.000448425 |
| CFAP43 | 1.026211591 | 1.740676155 | 0.496057433 | 3.509021416 | 0.000449759 |
| RNU5E-10P | 1.542978616 | 1.758429172 | 0.501267522 | 3.507965496 | 0.000451548 |
| ZNF683 | 1.752951982 | 1.070700319 | 0.305273725 | 3.507345147 | 0.000452602 |
| MARCO | 140.5808094 | -1.058820233 | 0.302398824 | -3.501403278 | 0.000462815 |
| TRHDE | 37.66887004 | -1.016582091 | 0.291056023 | -3.492736832 | 0.000478097 |
| FOSL1 | 1.194116498 | -1.028172119 | 0.294524704 | -3.490953741 | 0.0004813 |
| CLEC6A | 1.850585954 | -1.260364759 | 0.361153576 | -3.489830485 | 0.000483327 |
| GPR84 | 0.803057183 | 1.573983211 | 0.451068681 | 3.489453549 | 0.000484009 |
| RORA | 1.979538719 | 1.052279413 | 0.302005009 | 3.484311124 | 0.000493406 |
| PCSK9 | 1.095367617 | 2.139024501 | 0.61486164 | 3.478871281 | 0.00050353 |
| RNU6-1111P | 2.956928412 | 1.1081176 | 0.319184584 | 3.471714037 | 0.000517147 |
| MAFA-AS1 | 2.519734621 | 1.054694632 | 0.304127905 | 3.467931139 | 0.000524482 |
| KRT23 | 1.318307895 | -1.25383953 | 0.362174563 | -3.461975681 | 0.000536226 |
| DAAM2 | 0.903207257 | -1.222076153 | 0.353196172 | -3.460049255 | 0.000540077 |
| SPI1 | 1.573565302 | 1.063805382 | 0.308936011 | 3.443448943 | 0.000574345 |
| MIR5009 | 1.733531864 | 1.071934391 | 0.311535669 | 3.440807905 | 0.00057998 |
| BCAT1 | 0.974597023 | -1.162463363 | 0.338340302 | -3.43578154 | 0.000590847 |
| MIR646 | 2.117076311 | 1.126720663 | 0.328599592 | 3.428855944 | 0.000606131 |
| MIR1203 | 0.823567611 | 1.397564574 | 0.407925407 | 3.426029735 | 0.000612473 |
| FLJ46284 | 1.360535432 | 1.150802629 | 0.335982868 | 3.425182471 | 0.000614387 |
| GADD45A | 163.0597355 | 2.212999469 | 0.646139026 | 3.424958686 | 0.000614893 |
| PFKFB4 | 1.359743697 | -1.234478865 | 0.360801205 | -3.421493184 | 0.000622783 |
| SORT1 | 0.773753844 | 1.909794352 | 0.559514575 | 3.413305813 | 0.000641799 |
| FGF13 | 0.501253581 | -1.465350627 | 0.429956839 | -3.408134246 | 0.000654087 |
| WNT6 | 0.958268951 | -1.40437928 | 0.412256361 | -3.406567888 | 0.000657852 |
| TCTE1 | 0.791164947 | 1.780066422 | 0.52265571 | 3.40581072 | 0.000659679 |
| TTTY14 | 1.216124867 | 1.258153281 | 0.371847453 | 3.383519967 | 0.00071563 |
| BAZ1A | 1.139413887 | -1.103652254 | 0.326809931 | -3.377046254 | 0.000732687 |
| MCTP2 | 1.004571735 | 1.450751047 | 0.429714402 | 3.376081978 | 0.00073526 |
| RNU6-536P | 0.618305251 | 2.145531923 | 0.635973566 | 3.373618082 | 0.000741872 |
| C1QB | 0.840133608 | 1.469340695 | 0.435977345 | 3.37022259 | 0.000751075 |
| LIN7A | 2.257035551 | 1.325654461 | 0.394138506 | 3.363422855 | 0.000769823 |
| CBLL1-AS1 | 0.81622067 | 1.369122265 | 0.407185535 | 3.362403985 | 0.00077267 |
| MIR499B | 0.676688365 | 2.122258432 | 0.631538319 | 3.360458689 | 0.000778132 |
| ZYG11A | 1.844089166 | 1.079998782 | 0.322258866 | 3.351339233 | 0.000804217 |
| TRIM25 | 1.64475742 | 1.172752775 | 0.350030034 | 3.350434708 | 0.000806848 |
| SNORD71 | 2.139957109 | 1.330863321 | 0.397995154 | 3.343918406 | 0.00082604 |
| VNN2 | 3.886058449 | 1.55520017 | 0.465136091 | 3.343537942 | 0.000827174 |
| hCG_2039566 | 7.066008415 | 1.368119721 | 0.409999172 | 3.336884108 | 0.000847233 |
| IFNL1 | 0.934228343 | 1.264479984 | 0.379161259 | 3.334939828 | 0.000853179 |
| TRAJ5 | 2.041696212 | 1.056893579 | 0.316989549 | 3.334159064 | 0.000855577 |
| TRAJ20 | 0.596927955 | 1.689712886 | 0.507761382 | 3.327769591 | 0.000875442 |
| TEAD3 | 5.261764892 | 1.245092984 | 0.374448506 | 3.325138072 | 0.000883748 |
| CC2D2B | 0.873224557 | 1.856487887 | 0.558561526 | 3.323694525 | 0.000888334 |
| MIR7848 | 1.844979384 | 1.206985499 | 0.363915056 | 3.316668217 | 0.000910977 |
| DSCAML1 | 1.484482618 | 1.291717075 | 0.389854583 | 3.313330484 | 0.00092192 |
| PGDP1 | 0.527276968 | 1.712405665 | 0.516968573 | 3.312397997 | 0.000924999 |
| AGTPBP1 | 1.424285547 | 1.124956142 | 0.339982355 | 3.308866259 | 0.000936746 |
| PLSCR1 | 0.868032098 | -1.195259543 | 0.362080843 | -3.301084728 | 0.000963118 |
| RPL11P3 | 0.935392322 | 1.525646551 | 0.462216195 | 3.300720677 | 0.000964368 |
| NHERF4 | 1.393291779 | 1.116329731 | 0.338634919 | 3.296558237 | 0.000978773 |
| PELATON | 0.993490379 | 1.275127564 | 0.386977811 | 3.295092194 | 0.000983894 |
| RGS2 | 0.880664077 | -1.119912959 | 0.340275875 | -3.291191179 | 0.000997641 |
| FFAR3 | 0.577785359 | 1.538731507 | 0.467780729 | 3.289429023 | 0.001003909 |
| IMPDH1P10 | 0.702906573 | 1.448739548 | 0.440722929 | 3.287188964 | 0.001011929 |
| EXOC6 | 28.78196731 | -1.066225728 | 0.325008593 | -3.28060781 | 0.001035837 |
| NTSR1 | 1.31023084 | 1.865966882 | 0.568999472 | 3.27938245 | 0.001040345 |
| LRG1 | 0.902442982 | 1.335794147 | 0.407742128 | 3.276075866 | 0.001052603 |
| CDA | 0.570918247 | 1.731319296 | 0.529703389 | 3.268469358 | 0.001081309 |
| PFKFB2 | 3.004698498 | 1.017391426 | 0.311524676 | 3.265845384 | 0.001091378 |
| MIR4435-2HG | 0.978257324 | 1.42983565 | 0.437861633 | 3.265496545 | 0.001092723 |
| ANXA3 | 0.825327826 | 1.398024056 | 0.428641602 | 3.261522098 | 0.001108158 |
| COL9A3 | 2.05384929 | 1.071859677 | 0.329404348 | 3.253932995 | 0.001138191 |
| FAM81B | 2.225316177 | 1.097147548 | 0.337187719 | 3.253818228 | 0.001138651 |
| RPL31P57 | 2.123914588 | 1.094103101 | 0.336255831 | 3.253781791 | 0.001138797 |
| IGLC6 | 1.980740815 | 1.140117125 | 0.351091081 | 3.247354281 | 0.001164833 |
| GRIP1 | 1.339113748 | 1.271156917 | 0.392323278 | 3.240075187 | 0.001194982 |
| MIR4271 | 1.210675695 | -1.096129298 | 0.339225134 | -3.231273826 | 0.001232398 |
| SYNPO | 13.05718446 | -1.138961554 | 0.352533438 | -3.230790136 | 0.001234485 |
| NFE2 | 13.88955123 | 1.12690036 | 0.349415573 | 3.225100561 | 0.001259284 |
| CEBPB | 1.554367382 | 1.039439163 | 0.323616336 | 3.211948986 | 0.001318378 |
| TGM5 | 1.401959152 | 1.573142235 | 0.489996223 | 3.210519101 | 0.001324955 |
| BEAN1 | 3.408739033 | 1.214058018 | 0.379125299 | 3.20226063 | 0.001363536 |
| SFN | 0.672179987 | 1.387981077 | 0.433578603 | 3.201221343 | 0.001368464 |
| ERVH48-1 | 0.798382713 | -1.079409493 | 0.33759583 | -3.197342496 | 0.001387001 |
| ADAM32 | 0.649602376 | 1.471359816 | 0.460440686 | 3.195546919 | 0.001395661 |
| SULT1B1 | 1.191330248 | 1.106511374 | 0.346843608 | 3.190231409 | 0.001421589 |
| SLC1A3 | 1.123791753 | -1.000125471 | 0.313862871 | -3.186504565 | 0.001440032 |
| NSUN7 | 4.758685846 | 1.442107575 | 0.453240401 | 3.181771908 | 0.00146377 |
| SNORD89 | 0.684060091 | -1.237477567 | 0.389167398 | -3.179807906 | 0.001473727 |
| APOD | 2.236784099 | 1.218169431 | 0.383550352 | 3.17603523 | 0.001493028 |
| MTND6P5 | 0.734006214 | -1.232517228 | 0.388145779 | -3.175397737 | 0.001496313 |
| TUBB4A | 1.431877827 | 1.228702353 | 0.387039543 | 3.174617102 | 0.001500343 |
| SOCS3-DT | 0.896467044 | 1.49913705 | 0.473594985 | 3.165441142 | 0.00154848 |
| PIWIL4-AS1 | 0.559757562 | -1.254647866 | 0.396595771 | -3.163543232 | 0.001558612 |
| SQOR | 1.366055241 | 1.002844582 | 0.318308933 | 3.150538608 | 0.001629697 |
| RNU6-549P | 1.284208162 | 1.20323513 | 0.38203228 | 3.149564038 | 0.001635143 |
| SAMSN1 | 0.94656651 | 1.161446056 | 0.369619952 | 3.142270997 | 0.001676428 |
| TLR2 | 0.605076464 | -1.247571619 | 0.397072887 | -3.141920939 | 0.001678434 |
| FLOT1 | 1.022791707 | 1.503840775 | 0.479986724 | 3.133088273 | 0.001729774 |
| SEPTIN5 | 0.518923899 | -1.251730671 | 0.399837185 | -3.130600952 | 0.00174449 |
| MILR1 | 0.942030689 | 1.653690784 | 0.528426426 | 3.129462689 | 0.001751263 |
| FGF13-AS1 | 2.038616295 | 1.176252075 | 0.375980016 | 3.128496266 | 0.001757032 |
| SH2D4B | 0.807900494 | -1.069648408 | 0.341908843 | -3.128460791 | 0.001757245 |
| CD82 | 1.609851983 | 1.060349343 | 0.33984167 | 3.120127505 | 0.001807728 |
| SERPINB1 | 36.99749204 | 1.19949557 | 0.384565 | 3.119097084 | 0.001814062 |
| RNF224 | 0.883213229 | 1.305093791 | 0.41864 | 3.117460808 | 0.001824162 |
| FNDC5 | 0.952481385 | 1.58815816 | 0.509772828 | 3.115423329 | 0.001836811 |
| ZNF705A | 1.148677733 | 1.194272777 | 0.384387982 | 3.106946191 | 0.001890308 |
| TAS2R3 | 0.830221871 | 1.927027565 | 0.620669773 | 3.104754975 | 0.001904367 |
| PLB1 | 1.166443839 | 1.640144753 | 0.52872657 | 3.102066067 | 0.001921751 |
| DAPK1-IT1 | 1.182160933 | 1.310021463 | 0.422373599 | 3.101570427 | 0.001924971 |
| CSF2RA | 0.64338309 | 1.442819135 | 0.465230047 | 3.101302559 | 0.001926713 |
| NUDT16-DT | 1.839076108 | 1.291334192 | 0.417043023 | 3.096405217 | 0.001958825 |
| MIR6753 | 0.479251093 | 1.803001404 | 0.582565306 | 3.094934397 | 0.001968564 |
| FN1 | 7.361959152 | 1.130623335 | 0.365988528 | 3.089231623 | 0.002006749 |
| SYN3 | 1.258375622 | 1.100427616 | 0.356753071 | 3.08456382 | 0.002038509 |
| MTX1 | 1.27064264 | 1.09413708 | 0.35515649 | 3.080718248 | 0.00206502 |
| MMP8 | 2.978159138 | 1.33715846 | 0.434628429 | 3.076555445 | 0.002094074 |
| ANO10 | 1.60064233 | 1.226415353 | 0.398873568 | 3.074696973 | 0.002107165 |
| FCER1G | 0.940869366 | -1.034815085 | 0.336972504 | -3.07091847 | 0.002134014 |
| RNU6-37P | 0.93420819 | -1.079211581 | 0.352041131 | -3.065583777 | 0.002172455 |
| UBE2J1 | 1.350755636 | -1.268663417 | 0.414597574 | -3.059987556 | 0.002213462 |
| MYO7A | 1.532054127 | -1.032722341 | 0.338403659 | -3.05174697 | 0.002275138 |
| MTND5P12 | 1.143368317 | -1.425626208 | 0.467548703 | -3.049150168 | 0.002294897 |
| ALPK1 | 0.675673457 | -1.414957071 | 0.464702697 | -3.044865202 | 0.002327846 |
| RAD51 | 1.330789979 | 1.249068548 | 0.410929428 | 3.039618154 | 0.002368783 |
| SHROOM4 | 1.171424151 | 1.101841118 | 0.362977793 | 3.035560686 | 0.002400889 |
| RN7SL57P | 1.41552653 | 1.188831064 | 0.392120339 | 3.031801583 | 0.002430989 |
| RNASE1 | 0.435929219 | 1.606293337 | 0.529869532 | 3.031488392 | 0.002433513 |
| TVP23CP1 | 0.418274269 | 1.442919543 | 0.476355858 | 3.029079035 | 0.002453005 |
| SMIM10 | 1.084949702 | 1.162568826 | 0.384476868 | 3.023767939 | 0.002496479 |
| MIR124-1HG | 0.921262062 | 1.565551077 | 0.518476854 | 3.019519703 | 0.002531758 |
| P4HA3 | 0.75779499 | -1.118153937 | 0.370553115 | -3.017526753 | 0.002548465 |
| IFITM2 | 0.577744481 | 1.592539064 | 0.528119463 | 3.015490196 | 0.002565642 |
| CD8B2 | 1.633823577 | 1.27662396 | 0.423693141 | 3.01308621 | 0.002586054 |
| MIR4668 | 0.580440196 | 1.654642139 | 0.549167341 | 3.013001711 | 0.002586775 |
| PTPRK | 1.315231268 | -1.129513432 | 0.375092487 | -3.011293141 | 0.002601376 |
| RNU6-313P | 5.795392814 | -1.135790764 | 0.378084375 | -3.004066918 | 0.002663967 |
| MYO10 | 0.984621793 | 1.201930602 | 0.40215611 | 2.988716501 | 0.002801519 |
| MANSC1 | 0.560889067 | -1.18297203 | 0.396501803 | -2.983522448 | 0.002849511 |
| DHRS13 | 4.120190923 | 1.046938017 | 0.351115498 | 2.981748239 | 0.002866076 |
| NPAS2 | 0.882161709 | 1.164847328 | 0.391891703 | 2.972370475 | 0.002955099 |
| GALNT14 | 0.876037313 | 1.262834624 | 0.42566158 | 2.966757357 | 0.003009584 |
| SLC22A14 | 0.844179634 | 1.229579483 | 0.414674485 | 2.965167923 | 0.003025178 |
| IHO1 | 0.762500723 | -1.056218012 | 0.356410921 | -2.963483863 | 0.00304178 |
| LILRA6 | 0.90299601 | 1.239685857 | 0.419290261 | 2.956629267 | 0.003110218 |
| F5 | 0.73953728 | 1.412587853 | 0.478860171 | 2.949896312 | 0.003178806 |
| SNX7 | 0.726183027 | 1.302922387 | 0.441920454 | 2.948318809 | 0.003195074 |
| ACVR1C | 1.760373418 | 1.016810445 | 0.345176798 | 2.945767067 | 0.003221549 |
| CPNE4 | 1.501584933 | 1.068127846 | 0.363177129 | 2.941065837 | 0.00327085 |
| EPCAM | 1.554939619 | 1.089289257 | 0.370775101 | 2.937870566 | 0.00330475 |
| NUDT19P5 | 1.222943491 | 1.055245333 | 0.359844166 | 2.932506433 | 0.00336238 |
| PKD2L1 | 1.292268298 | -1.020603815 | 0.348346412 | -2.929853101 | 0.003391223 |
| RNU6-1300P | 1.130300652 | 1.136356633 | 0.388535505 | 2.924717609 | 0.003447689 |
| CD163 | 0.582017557 | -1.131703436 | 0.387170841 | -2.923007921 | 0.003466677 |
| ORM2 | 0.974748114 | 1.201229108 | 0.411446083 | 2.919529814 | 0.003505599 |
| FLT1P1 | 0.723259618 | -1.129051225 | 0.38673762 | -2.919424351 | 0.003506785 |
| IGHV4-4 | 1.718900502 | 1.099030209 | 0.376640147 | 2.917984757 | 0.003523016 |
| RPL7P18 | 0.551684271 | 1.754075723 | 0.601693794 | 2.915229872 | 0.003554266 |
| CACNA2D1 | 0.444073168 | 1.323627038 | 0.454405274 | 2.912877808 | 0.003581147 |
| KIAA1217 | 0.442721836 | 1.489438099 | 0.511714989 | 2.910679051 | 0.003606443 |
| SLC51A | 1.128703894 | -1.175249997 | 0.404002634 | -2.909015679 | 0.003625687 |
| HLA-DQA2 | 0.599952777 | 1.389726012 | 0.477816016 | 2.908496082 | 0.003631717 |
| NAMPTP3 | 0.749866596 | 1.310637528 | 0.450968585 | 2.906272349 | 0.003657629 |
| C1QC | 0.864972059 | 1.534126406 | 0.528034364 | 2.905353343 | 0.003668387 |
| CA4 | 0.880970292 | 1.313965413 | 0.452400139 | 2.904431941 | 0.003679202 |
| Cromer blood group | 0.42230372 | 1.702903387 | 0.587325111 | 2.899422066 | 0.003738513 |
| RN7SL473P | 0.447326508 | 1.492545067 | 0.515381773 | 2.895998937 | 0.003779537 |
| SLC4A9 | 0.554187331 | 1.335813541 | 0.462123249 | 2.890600169 | 0.00384507 |
| MIR635 | 1.07646609 | 1.153100202 | 0.398925971 | 2.890511736 | 0.003846152 |
| HNRNPA1P70 | 1.21773755 | 1.032196201 | 0.357321696 | 2.888702852 | 0.003868344 |
| RN7SKP26 | 0.762307381 | 1.538222991 | 0.533576285 | 2.88285487 | 0.00394089 |
| HUNK | 0.579377337 | 1.616410442 | 0.561658563 | 2.877923613 | 0.004003021 |
| DRAM1 | 0.975480426 | -1.069153811 | 0.371858456 | -2.875163368 | 0.004038185 |
| SLC49A4 | 0.543224137 | 1.484405747 | 0.516310519 | 2.875025189 | 0.004039953 |
| SCRG1 | 0.56756585 | 1.627916326 | 0.567633314 | 2.86790131 | 0.004132044 |
| IGHJ5 | 0.460122933 | 1.587874357 | 0.55448972 | 2.863667799 | 0.004187669 |
| RNU6-196P | 0.420992697 | 1.43761119 | 0.502893985 | 2.858676447 | 0.004254124 |
| LMNB1-DT | 44.68722134 | 1.064010913 | 0.372381285 | 2.857315754 | 0.004272406 |
| TM4SF1 | 0.863823902 | 1.49737202 | 0.524184564 | 2.856574045 | 0.004282401 |
| SLC4A10 | 0.681415786 | 1.401649926 | 0.49329055 | 2.841428701 | 0.004491189 |
| MKNK1-AS1 | 1.617018401 | 1.001569882 | 0.353664121 | 2.831980463 | 0.004626068 |
| EFCAB2 | 0.642748529 | 1.314207991 | 0.464397004 | 2.829923493 | 0.004655914 |
| TNFSF13B | 0.911041854 | 1.074469701 | 0.380274236 | 2.825512749 | 0.004720501 |
| SDR42E1P5 | 0.842886597 | 1.091049103 | 0.38819885 | 2.810541821 | 0.004945816 |
| USB1 | 3.411779289 | 1.583618093 | 0.563720914 | 2.809223595 | 0.004966114 |
| GJB6 | 0.446788464 | 1.568595576 | 0.55946878 | 2.803723159 | 0.005051626 |
| IL10 | 1.129641059 | 1.053048954 | 0.375627053 | 2.803442789 | 0.00505602 |
| KCNH7 | 0.619814014 | 1.892795828 | 0.675516976 | 2.801995947 | 0.005078751 |
| CFAP126 | 0.474682085 | 1.441733515 | 0.514809932 | 2.800516123 | 0.005102096 |
| CYP7A1 | 0.546152871 | 1.470445463 | 0.52565941 | 2.797334996 | 0.005152608 |
| NTN4 | 1.115206533 | -1.122219515 | 0.401569179 | -2.794585776 | 0.005196625 |
| DUSP13 | 2.72930528 | 1.255943847 | 0.449848271 | 2.791927694 | 0.005239506 |
| IL1R1 | 0.742978199 | 1.261093965 | 0.451822811 | 2.791125044 | 0.005252518 |
| S100A6 | 0.537039691 | 1.300982709 | 0.467234287 | 2.784433305 | 0.005362135 |
| SSU | 0.494971833 | 1.312928531 | 0.472081221 | 2.781149669 | 0.005416676 |
| CACNA2D2 | 1.382112251 | 1.433080104 | 0.516474801 | 2.774733833 | 0.00552469 |
| NT5C3AP1 | 0.602772011 | 1.287982073 | 0.464740432 | 2.771400947 | 0.005581565 |
| TRPM2 | 1.272124694 | -1.107668995 | 0.399874891 | -2.770038877 | 0.00560496 |
| EPPK1 | 1.442758757 | 1.290925929 | 0.466500494 | 2.767255223 | 0.005653048 |
| FFAR2 | 0.688941752 | 1.180005589 | 0.42669338 | 2.765464955 | 0.005684171 |
| ATP6V1C1 | 0.467140318 | 1.34288389 | 0.485737298 | 2.764629965 | 0.00569874 |
| PTGR1 | 1.238911638 | 1.371771438 | 0.497816972 | 2.755573866 | 0.005858927 |
| DGAT2 | 0.694932027 | -1.201515545 | 0.436050643 | -2.755449542 | 0.005861154 |
| LHFPL5 | 1.150124344 | 1.054839747 | 0.383081088 | 2.753567796 | 0.005894956 |
| PROS1 | 0.35263073 | 1.662376659 | 0.605021311 | 2.74763323 | 0.006002712 |
| LAMTOR5 | 1.204628922 | 1.038799374 | 0.378248819 | 2.746338711 | 0.006026451 |
| DGCR5 | 2.759661462 | 1.298344525 | 0.473823195 | 2.740145561 | 0.006141198 |
| NR3C2 | 0.743607411 | -1.448315722 | 0.529215093 | -2.736724145 | 0.00620543 |
| NDNF | 0.770324583 | -1.205402796 | 0.440640356 | -2.735570581 | 0.006227223 |
| FAM169BP | 0.505834315 | -1.349315796 | 0.494087113 | -2.730926915 | 0.006315648 |
| C1RL | 0.465863694 | 1.346636082 | 0.493246106 | 2.730150456 | 0.006330543 |
| GATA3 | 0.775680412 | -1.680235861 | 0.615829532 | -2.72841066 | 0.006364033 |
| RPL35AP26 | 0.66890843 | 1.36443345 | 0.500370926 | 2.726843982 | 0.006394327 |
| TRDJ4 | 1.169292635 | 1.293504961 | 0.474446609 | 2.726344623 | 0.00640401 |
| S100A7 | 0.916134741 | 1.54569502 | 0.567273361 | 2.724779845 | 0.006434438 |
| SYNC | 0.867979888 | 1.186511666 | 0.43567102 | 2.723411961 | 0.006461143 |
| EPHX4 | 0.544512539 | 1.207049896 | 0.443865274 | 2.719406016 | 0.006539928 |
| SH3PXD2B | 0.532711903 | 1.332527595 | 0.490332785 | 2.717598407 | 0.00657576 |
| DRC1 | 0.49848938 | 1.336230891 | 0.491718476 | 2.71747139 | 0.006578284 |
| S100A9 | 0.613954999 | 1.310481212 | 0.482637414 | 2.715249945 | 0.006622577 |
| FOXQ1 | 1.387305089 | 1.012618755 | 0.373075511 | 2.714246114 | 0.00664268 |
| IRAK3 | 0.512390023 | -1.078391661 | 0.399177798 | -2.701532165 | 0.00690208 |
| GEM | 0.657724055 | 1.085446543 | 0.402536663 | 2.696515978 | 0.007006904 |
| CIR1 | 1.816059421 | -1.030738939 | 0.38275325 | -2.69295934 | 0.007082091 |
| TRDJ3 | 0.777256178 | 1.123363057 | 0.417477935 | 2.69083217 | 0.007127404 |
| TLR5 | 0.666246851 | 2.057963482 | 0.765077069 | 2.689877353 | 0.007147828 |
| FABP2 | 0.510517897 | 1.306750656 | 0.485811391 | 2.689831241 | 0.007148816 |
| TRGV9 | 0.57113993 | 1.422987388 | 0.529335238 | 2.688253653 | 0.007182681 |
| C9JAW5_HUMAN | 0.641549328 | 1.163449899 | 0.435397288 | 2.672156971 | 0.007536538 |
| DAB2IP | 0.554827552 | 1.258729128 | 0.471091699 | 2.671940794 | 0.007541395 |
| IL1R2 | 1.188174938 | 1.123389697 | 0.420632856 | 2.670713143 | 0.00756903 |
| PCOLCE2 | 0.675943857 | 1.113642446 | 0.417689944 | 2.666194058 | 0.00767154 |
| HMGN1P32 | 0.663924853 | -1.039172265 | 0.39003434 | -2.664309675 | 0.007714651 |
| GDF15 | 0.729742715 | -1.018318086 | 0.382954088 | -2.65911272 | 0.007834675 |
| IMPA2 | 2.196983291 | 1.095695669 | 0.412140035 | 2.658551888 | 0.007847727 |
| KSR2 | 0.512701054 | 1.342642009 | 0.505150514 | 2.657904864 | 0.007862809 |
| ALPL | 0.418503379 | 1.399985189 | 0.527029466 | 2.656369858 | 0.007898693 |
| CRYGN | 0.467225973 | 1.345716944 | 0.507467408 | 2.651829304 | 0.0080057 |
| BMP2 | 0.922601185 | 1.134582734 | 0.42788689 | 2.651594988 | 0.008011258 |
| FCGR1A | 0.437749887 | 1.377712953 | 0.519700855 | 2.650973033 | 0.008026025 |
| NOXRED1 | 30.48175222 | 1.273243361 | 0.481101509 | 2.646517081 | 0.00813254 |
| LRRN1 | 1.474309241 | 1.092015896 | 0.412763972 | 2.645618245 | 0.008154178 |
| ROPN1L | 0.544874566 | 1.161760343 | 0.439171184 | 2.645347387 | 0.008160709 |
| TRAJ18 | 1.301466286 | 1.12606769 | 0.425755155 | 2.644871532 | 0.008172194 |
| DEFA4 | 0.914688089 | -1.026092934 | 0.38804354 | -2.644272688 | 0.008186667 |
| LILRB4 | 0.558932251 | 1.624711645 | 0.616728107 | 2.63440506 | 0.008428487 |
| rat | 1.431102369 | 1.371325384 | 0.520550057 | 2.634377551 | 0.00842917 |
| RNU6-1062P | 0.724675411 | 1.930461409 | 0.73357119 | 2.631593819 | 0.00849854 |
| RPL21P20 | 0.41271621 | 1.347319173 | 0.512791527 | 2.627420897 | 0.008603484 |
| TWIST2 | 0.87488781 | 1.057364061 | 0.402945595 | 2.624086412 | 0.008688174 |
| CRISP2 | 0.509060817 | 1.18739952 | 0.452995757 | 2.621215546 | 0.008761685 |
| N-acetyl | 0.663221565 | 1.389552271 | 0.53162585 | 2.6137786 | 0.008954704 |
| GPR42 | 1.085507416 | 1.241422442 | 0.475194759 | 2.612449777 | 0.00898959 |
| CLEC4D | 0.544369431 | 1.37586089 | 0.526688453 | 2.612286035 | 0.008993897 |
| NOG | 6.833219764 | 1.006274692 | 0.385448322 | 2.610660452 | 0.009036757 |
| LIPC-AS1 | 0.885094018 | 1.217585019 | 0.467024147 | 2.607113628 | 0.009130905 |
| EXPH5 | 0.952243967 | 1.208802982 | 0.464387407 | 2.603005518 | 0.009241047 |
| RNU6-377P | 1.678089385 | 2.02813701 | 0.780103527 | 2.599830587 | 0.009326979 |
| RNU1-134P | 0.640103267 | 1.16168146 | 0.44735881 | 2.596755519 | 0.009410888 |
| RGSL1 | 0.606865526 | 1.162115227 | 0.448082166 | 2.593531532 | 0.009499583 |
| OMG | 1.062766226 | 1.02597696 | 0.396666998 | 2.586494379 | 0.009695775 |
| BEST3 | 0.43888392 | 1.732818261 | 0.670315528 | 2.585078501 | 0.009735683 |
| TMSB4Y | 0.809215309 | -1.019564734 | 0.394758261 | -2.582757182 | 0.009801428 |
| CAVIN3 | 0.589213109 | -1.044131714 | 0.404628565 | -2.580469603 | 0.009866604 |
| BST1 | 0.990113715 | 1.271940868 | 0.49313639 | 2.579288189 | 0.009900415 |
| H2BC21 | 0.804602466 | 1.085695113 | 0.421751491 | 2.574253168 | 0.010045674 |
| CATIP-AS1 | 0.622504267 | -1.231435685 | 0.478448087 | -2.573812536 | 0.010058476 |
| CD46P1 | 0.525633096 | 1.360169858 | 0.528648698 | 2.572918204 | 0.010084504 |
| TPST1 | 0.789315836 | 1.020229332 | 0.396636774 | 2.572200559 | 0.010105433 |
| ANKRD22 | 0.565634113 | -1.04053329 | 0.405213125 | -2.567866698 | 0.01023265 |
| C9orf152 | 0.446498276 | -1.122358295 | 0.437811416 | -2.563565621 | 0.010360311 |
| STOX2 | 1.514260099 | 1.461827371 | 0.570861893 | 2.560737352 | 0.010445029 |
| NDUFB8P2 | 0.623852145 | -1.029104088 | 0.401919651 | -2.560472191 | 0.010453003 |
| MIR6124 | 0.748873133 | -1.11201618 | 0.434853741 | -2.55721884 | 0.010551282 |
| OPLAH | 0.658709637 | 1.144432929 | 0.447772913 | 2.555833316 | 0.010593385 |
| RBM20 | 0.63718827 | 1.160608102 | 0.454197025 | 2.555296577 | 0.010609736 |
| ITGA7 | 0.986183005 | 1.064592991 | 0.416840737 | 2.553956214 | 0.010650665 |
| OR2L13 | 1.118037306 | -1.012993475 | 0.396686008 | -2.553640547 | 0.010660325 |
| MAFG | 0.478741572 | 1.357774469 | 0.53343844 | 2.545325509 | 0.010917591 |
| LHX4 | 0.635385333 | 1.108305838 | 0.436677715 | 2.538040757 | 0.0111475 |
| TRPV4 | 0.658212312 | 2.044573503 | 0.805972114 | 2.536779458 | 0.011187741 |
| CYP1A1 | 0.362012126 | 1.386070374 | 0.547733975 | 2.530553948 | 0.011388257 |
| OR2A7 | 0.607166383 | 1.069957641 | 0.423695147 | 2.525300678 | 0.011559934 |
| POU5F1 | 0.702292833 | 1.091252049 | 0.432872561 | 2.520954544 | 0.011703697 |
| TBC1D8 | 0.75600437 | 1.062932691 | 0.42164725 | 2.520905069 | 0.011705343 |
| TRAJ56 | 0.886206528 | 1.210885664 | 0.481632238 | 2.514129184 | 0.011932672 |
| SHOX2 | 89.21041912 | -1.018107161 | 0.406011203 | -2.507583913 | 0.012155971 |
| RAB32 | 0.91598108 | 1.062852168 | 0.424599009 | 2.503190413 | 0.012307931 |
| CPXM2 | 0.420581381 | 1.511636783 | 0.604958066 | 2.498746388 | 0.012463347 |
| IL1RN | 1.203895359 | 1.296542423 | 0.519215281 | 2.497119152 | 0.012520688 |
| GLDN | 0.838580077 | 1.05107225 | 0.421063492 | 2.496232207 | 0.01255204 |
| DSC2 | 0.464107684 | 1.180414645 | 0.473192031 | 2.494578453 | 0.012610685 |
| RNU6-268P | 0.4658697 | -1.025203552 | 0.411001412 | -2.494403966 | 0.012616886 |
| SDR16C5 | 0.53293563 | 1.162107378 | 0.466737433 | 2.489852528 | 0.012779611 |
| KAZN | 0.550366929 | 1.14345457 | 0.459594575 | 2.487963593 | 0.012847688 |
| INSYN2A | 0.541476237 | 1.347397518 | 0.542285033 | 2.484666618 | 0.012967281 |
| OSCAR | 0.781716332 | 1.013667491 | 0.410558855 | 2.468994347 | 0.013549336 |
| DRC7 | 0.494540155 | 1.154377221 | 0.468848332 | 2.462154909 | 0.0138105 |
| FBXL5 | 0.582265204 | 1.104361315 | 0.448821395 | 2.460580816 | 0.013871233 |
| ADAMTS3 | 0.709153907 | 1.759028695 | 0.715380821 | 2.458870356 | 0.013937494 |
| PRB3 | 0.649950539 | 1.07339039 | 0.436737052 | 2.45774977 | 0.013981055 |
| ERFE | 0.910247198 | 1.025490271 | 0.417264888 | 2.457648127 | 0.013985012 |
| SPON1 | 0.632401878 | 1.031654917 | 0.421955949 | 2.444935115 | 0.01448782 |
| PRDX6-AS1 | 0.946957311 | 1.080080868 | 0.441766784 | 2.444911901 | 0.014488753 |
| PLAU | 0.477998897 | -1.066652996 | 0.436354503 | -2.444464279 | 0.014506745 |
| RNU7-19P | 0.696140309 | 1.327945431 | 0.543420616 | 2.443678786 | 0.014538364 |
| SYN2 | 0.330507872 | 1.371879098 | 0.562187657 | 2.440251187 | 0.014677053 |
| NKD1 | 0.810173954 | 1.032091027 | 0.423374074 | 2.437775694 | 0.014777941 |
| PLBD1 | 1.103281242 | 1.186986577 | 0.487882788 | 2.432933906 | 0.014977034 |
| MIR3945HG | 0.627611259 | 1.182164223 | 0.486010602 | 2.432383611 | 0.014999811 |
| RPL26P5 | 0.643904462 | 1.145508809 | 0.471754759 | 2.428187075 | 0.015174514 |
| CLEC18A | 0.686168056 | -1.041427794 | 0.429040019 | -2.427344182 | 0.015209819 |
| GPC3 | 0.854247103 | 1.056933329 | 0.436428841 | 2.421776999 | 0.015444825 |
| S100A11 | 0.797556802 | 1.176530081 | 0.485878015 | 2.42145157 | 0.015458661 |
| HAT1 | 0.432702663 | 1.234858849 | 0.51055663 | 2.418652065 | 0.015578133 |
| RPS2P40 | 0.623501498 | 2.45861298 | 1.017742392 | 2.415751766 | 0.015702762 |
| KLRC3 | 0.341710822 | 1.36005257 | 0.5633661 | 2.41415408 | 0.015771791 |
| LOXHD1 | 0.842227616 | 1.033470432 | 0.428188662 | 2.413586635 | 0.015796372 |
| GAL3ST1 | 0.610534195 | 1.035576796 | 0.430496875 | 2.405538476 | 0.016148651 |
| HEY1 | 0.530530078 | 1.327559887 | 0.552329785 | 2.40356382 | 0.016236133 |
| RNU7-103P | 0.574552385 | 1.3018623 | 0.54165912 | 2.403471579 | 0.016240229 |
| PPBP | 0.680760299 | -1.001775426 | 0.417026113 | -2.402188721 | 0.016297298 |
| SGCD | 0.423137548 | 1.266097592 | 0.527231374 | 2.401407907 | 0.016332119 |
| CYYR1 | 0.465703893 | 1.127732475 | 0.470253415 | 2.398137767 | 0.016478666 |
| CAMP | 0.51756584 | 1.111165429 | 0.465060351 | 2.389292973 | 0.016880835 |
| EXOSC4 | 0.329449085 | -2.046479861 | 0.857043886 | -2.387835552 | 0.016947923 |
| FRMPD3 | 0.727476863 | 1.016518253 | 0.428695089 | 2.37119174 | 0.01773083 |
| HSDL2 | 0.877960744 | 1.163543964 | 0.490995538 | 2.369764844 | 0.017799402 |
| FUT7 | 0.577696941 | 1.211717546 | 0.512723049 | 2.36329837 | 0.01811308 |
| IRAG1 | 5.339558719 | 1.480271581 | 0.627053839 | 2.360677009 | 0.01824161 |
| VSIG4 | 0.711491682 | 1.04237473 | 0.443210843 | 2.351871002 | 0.018679252 |
| RNU1-91P | 0.509419279 | 1.218288643 | 0.518276952 | 2.350651787 | 0.018740562 |
| RNU6-917P | 0.593049757 | 1.231090063 | 0.525750162 | 2.341587605 | 0.019201919 |
| CFAP73 | 0.416383027 | 1.189463014 | 0.509458909 | 2.334757512 | 0.019556092 |
| TSPO | 0.589221178 | -1.050374911 | 0.450569128 | -2.331218111 | 0.019741862 |
| CMTM2 | 0.357734559 | 1.236797618 | 0.531423276 | 2.327330537 | 0.01994768 |
| MMP27 | 0.547452954 | 1.002816653 | 0.432414501 | 2.319109674 | 0.02038909 |
| MXD3 | 0.540318448 | 1.595972255 | 0.688453693 | 2.31819841 | 0.02043854 |
| CDKN2D | 0.812282531 | 1.01710414 | 0.438867849 | 2.317563572 | 0.020473052 |
| SIGLEC11 | 0.543616743 | 1.026842565 | 0.443657825 | 2.314492174 | 0.02064074 |
| GPR27 | 0.463141362 | 1.010777246 | 0.437142683 | 2.31223645 | 0.020764657 |
| CASP5 | 0.462464845 | 1.147643569 | 0.496565384 | 2.311163055 | 0.02082385 |
| CD59 blood group | 0.917671279 | -1.073535269 | 0.464512381 | -2.311101518 | 0.020827248 |
| CES1 | 0.53107191 | 1.264901347 | 0.54824296 | 2.307191226 | 0.021044162 |
| NLRP12 | 0.970120084 | 1.105197938 | 0.480974446 | 2.297830886 | 0.021571416 |
| LILRP2 | 0.751668141 | 1.000545626 | 0.435822016 | 2.295766593 | 0.021689231 |
| CLEC4E | 0.515760126 | 1.110067741 | 0.483791659 | 2.294516078 | 0.021760873 |
| TDRD9 | 1.636309959 | 1.236452888 | 0.540331193 | 2.288324097 | 0.022118654 |
| ALOX15B | 0.372175513 | -1.194408558 | 0.522416354 | -2.286315404 | 0.022235813 |
| GGH | 0.464017476 | 1.104224068 | 0.483361887 | 2.284466562 | 0.022344126 |
| TGFA | 0.677223737 | 1.03088356 | 0.451826684 | 2.281590699 | 0.022513516 |
| SERPINB2 | 0.430663843 | 1.209849913 | 0.530488668 | 2.280632909 | 0.022570178 |
| LRRC70 | 0.53207318 | 1.045171628 | 0.458424022 | 2.27992334 | 0.022612235 |
| FAM178B | 0.550980345 | 1.149267579 | 0.504424427 | 2.278374159 | 0.022704295 |
| TMED11P | 0.496644137 | 1.128614751 | 0.495584035 | 2.277342837 | 0.022765761 |
| MIR548AY | 0.542340415 | 1.312946758 | 0.577461247 | 2.273653453 | 0.022986831 |
| AQP11 | 3.146841877 | 1.129446526 | 0.49724459 | 2.271410388 | 0.023122146 |
| RNU7-12P | 0.670213767 | 1.248437992 | 0.551203017 | 2.264933162 | 0.023516781 |
| DDX10P1 | 0.416749016 | -1.164815391 | 0.514714673 | -2.263031251 | 0.023633763 |
| RPL7P60 | 0.486345568 | 1.115224119 | 0.492931129 | 2.262433945 | 0.023670606 |
| RPL27P10 | 0.438413695 | 1.184288071 | 0.523812068 | 2.260902609 | 0.023765289 |
| TMCO3 | 4.300793635 | -1.126194034 | 0.499658647 | -2.253926838 | 0.024200771 |
| PRUNE2 | 0.546504411 | -1.24726265 | 0.553636489 | -2.25285485 | 0.024268302 |
| SFRP5 | 0.607688766 | 1.128780982 | 0.50288652 | 2.244603777 | 0.02479358 |
| S100P | 0.505361112 | -1.552064901 | 0.691900224 | -2.24319179 | 0.02488445 |
| SLCO2B1 | 0.361344175 | 1.190992594 | 0.533796584 | 2.231173127 | 0.025669663 |
| TRAV38-2DV8 | 0.692365293 | 1.006635215 | 0.452630706 | 2.223965812 | 0.026150745 |
| RAB13 | 0.355096482 | 1.52760573 | 0.688223326 | 2.219636666 | 0.026443441 |
| LYPD8 | 0.407067612 | -1.202134848 | 0.542082117 | -2.217624987 | 0.026580413 |
| IL18 | 0.488229472 | 1.099041627 | 0.496290235 | 2.214513909 | 0.026793447 |
| TNFRSF19 | 0.966741845 | 1.038603658 | 0.471799346 | 2.20136731 | 0.027710031 |
| ZMAT4 | 0.616933858 | 1.192182969 | 0.542323201 | 2.198288707 | 0.027928539 |
| ZNF438 | 0.805944554 | 1.031224169 | 0.469616911 | 2.195883804 | 0.028100261 |
| ITGA2B | 0.926211133 | -1.198954647 | 0.546072991 | -2.195594117 | 0.028121008 |
| TRAJ13 | 0.426159354 | 1.036058141 | 0.47199475 | 2.19506285 | 0.02815909 |
| RPL31P18 | 0.473447244 | 1.286160174 | 0.586673686 | 2.192292248 | 0.028358411 |
| MSTN | 0.6518947 | 1.084468695 | 0.495022046 | 2.190748276 | 0.028470013 |
| XCR1 | 0.568488369 | 1.68516948 | 0.771924617 | 2.183075191 | 0.029030273 |
| PPIGP1 | 0.342449012 | 1.167658894 | 0.534892328 | 2.182979327 | 0.029037332 |
| LTB4R | 0.63894936 | 1.005005314 | 0.460637846 | 2.181768876 | 0.029126595 |
| HTRA1 | 0.654815253 | -1.114539184 | 0.512562154 | -2.174446894 | 0.02967159 |
| TRBV15 | 0.551450412 | 1.133731797 | 0.522079989 | 2.171567233 | 0.029888321 |
| MSRB2 | 0.578079012 | 1.053149255 | 0.488478536 | 2.15597857 | 0.031085339 |
| SLC37A3 | 2339.867017 | -1.195454289 | 0.555973302 | -2.150200889 | 0.031539328 |
| DLC1 | 0.441604345 | -1.023385898 | 0.476356565 | -2.148361065 | 0.031685083 |
| SEMA3B | 0.893926461 | 1.471480117 | 0.685044232 | 2.148007455 | 0.031713163 |
| DUSP1 | 0.303970364 | 1.102200719 | 0.51485386 | 2.14080306 | 0.032289923 |
| PAQR6 | 0.876536512 | 1.103079184 | 0.515581778 | 2.139484425 | 0.032396456 |
| ADGRG2 | 0.351120758 | 1.316468701 | 0.616295117 | 2.136101139 | 0.032671171 |
| RN7SKP16 | 0.531119796 | 1.012960777 | 0.474246952 | 2.13593524 | 0.032684693 |
| PCDH18 | 0.51869688 | 1.193811939 | 0.560465114 | 2.130037908 | 0.033168484 |
| PLP2 | 0.366980835 | 1.197368358 | 0.564737469 | 2.120221206 | 0.033987395 |
| GMFG | 0.375748934 | -1.050325589 | 0.495567592 | -2.119439622 | 0.034053331 |
| RN7SL430P | 0.684215615 | 1.187214602 | 0.563468307 | 2.106976713 | 0.035119598 |
| IFNGR2 | 0.686391422 | 1.041676553 | 0.495366073 | 2.10284194 | 0.035479588 |
| DYSF | 0.463789513 | 1.026897522 | 0.488450449 | 2.102357617 | 0.035521961 |
| CNIH4 | 0.509245577 | 1.034443521 | 0.492766588 | 2.09925662 | 0.035794285 |
| TNNI2 | 0.26087532 | -1.0439015 | 0.499131659 | -2.091435156 | 0.036489073 |
| ECHDC3 | 2.841861479 | 1.438110707 | 0.690500087 | 2.082708944 | 0.037277762 |
| ZSCAN23 | 0.570078846 | 1.080353488 | 0.519345955 | 2.080219318 | 0.037505421 |
| RPEP6 | 0.351281704 | 1.017322166 | 0.489813597 | 2.076957792 | 0.037805455 |
| DKK3 | 0.500939889 | 1.709275187 | 0.823431506 | 2.075795224 | 0.037912894 |
| METTL9 | 0.400497127 | 1.075680915 | 0.518237434 | 2.075652675 | 0.037926086 |
| C1QTNF12 | 0.420161932 | 1.096180516 | 0.531756565 | 2.061432973 | 0.039261752 |
| BEST1 | 0.464490988 | 1.241648944 | 0.606540723 | 2.04709906 | 0.040648359 |
| PXT1 | 0.449016136 | 1.008792108 | 0.49445382 | 2.040215017 | 0.041328915 |
| RN7SL271P | 0.448594612 | 1.118192577 | 0.548685714 | 2.037947312 | 0.041555202 |
| HPR | 0.380796129 | -1.011857176 | 0.497331857 | -2.034571408 | 0.041894017 |
| FCGR1CP | 1.18518675 | 1.280469273 | 0.62959159 | 2.033809367 | 0.04197082 |
| RNU6-90P | 0.357684775 | 1.031759842 | 0.508903654 | 2.027416846 | 0.042619801 |
| ADGRE1 | 0.35486217 | -1.23750477 | 0.615311374 | -2.011184616 | 0.04430596 |
| CYP19A1 | 0.375660777 | 1.163813414 | 0.57979983 | 2.0072676 | 0.044721177 |
| TRAJ12 | 0.580443536 | 1.006167641 | 0.501592105 | 2.005947921 | 0.044861805 |
| RAB24 | 0.353869274 | 1.055257232 | 0.526488667 | 2.004330383 | 0.045034681 |
| ZNF341-AS1 | 0.463099848 | 1.034672687 | 0.516557183 | 2.003016746 | 0.045175491 |
| NSG2 | 0.697047665 | 1.050453706 | 0.525715085 | 1.998142599 | 0.045701203 |
| MMP1 | 0.370417366 | 1.127933162 | 0.567729416 | 1.986744266 | 0.046950744 |
| TRAJ6 | 0.32823652 | 1.157661692 | 0.58367983 | 1.983384781 | 0.047324466 |
| PODN | 0.582634104 | 1.020049857 | 0.514659855 | 1.981988388 | 0.04748054 |
| TRAJ47 | 0.44391395 | 1.035408367 | 0.523455414 | 1.978025903 | 0.047925785 |
| MIR4802 | 0.494532842 | 1.29742479 | 0.656444678 | 1.976441936 | 0.048104746 |
| OSMR | 0.417611703 | -1.051042476 | 0.532188722 | -1.97494316 | 0.048274599 |
| CARS1-AS1 | 0.337256436 | 1.152050618 | 0.583360464 | 1.974852066 | 0.048284938 |
| GPR160 | 0.476832028 | 1.468019599 | 0.745054812 | 1.970351141 | 0.048798141 |
| HNRNPH2 | 0.331333122 | 1.025500422 | 0.521152716 | 1.967754153 | 0.049096332 |
| ASGR1 | 0.318638793 | -1.245171639 | 0.633022257 | -1.967026633 | 0.04918014 |
| ARNT2 | 0.966230779 | -1.003305628 | 0.510627199 | -1.964849562 | 0.049431652 |
| GSDMA | 0.701414901 | 1.017689738 | 0.518399673 | 1.963137306 | 0.049630222 |
| MIR4257 | 0.30284031 | 1.127341212 | 0.574707557 | 1.961591071 | 0.049810113 |
