# Supplementary material for: IL27 and IL1RN are causally associated with acute pancreatitis: a Mendelian randomization study
Source: Aging (Albany NY). 2024 May 13;16(10):8572–84. doi: 10.18632/aging.205825 (PMC11164491; doi:10.18632/aging.205825)
Supplement: Supplementary Table 2 [file aging-16-205825-s002.doc]

Supplementary Table 2. All instrumental variation.

gene SNP effect_allele.exposure other_allele.exposure effect_allele.outcome other_allele.outcome beta.exposure beta.outcome eaf.exposure eaf.outcome id.outcome chr pos se.outcome samplesize.outcome pval.outcome outcome originalname.outcome outcome.deprecated mr_keep.outcome data_source.outcome pos.exposure chr.exposure samplesize.exposure pval.exposure se.exposure id.exposure exposure mr_keep.exposure R2 F

1 IL10 rs62143196 G A G A -0.1667 -0.0094 0.22404 0.1941 finn-b-K11_ACUTPANC 19 54320636 0.0339 NA 0.781801 Acute pancreatitis || id:finn-b-K11_ACUTPANC Acute pancreatitis Acute pancreatitis || || TRUE igd 54320636 19 3301 3.63E-08 0.0303 prot-a-1464 || id:prot-a-1464 TRUE 0.009086076 30.24981576

2 IL1B rs13402561 G C G C -0.3247 -0.0349 0.81414 0.6486 finn-b-K11_ACUTPANC 2 3640142 0.0281 NA 0.2138 Acute pancreatitis || id:finn-b-K11_ACUTPANC Acute pancreatitis Acute pancreatitis || || TRUE igd 3639921 2 3301 2.19E-23 0.0326 prot-a-1495 || id:prot-a-1495 TRUE 0.029175909 99.14393874

3 IL1B rs967645 T C T C -0.1427 -0.043 0.50945 0.4444 finn-b-K11_ACUTPANC 17 26713970 0.0267 NA 0.107 Acute pancreatitis || id:finn-b-K11_ACUTPANC Acute pancreatitis Acute pancreatitis || || TRUE igd 26713970 17 3301 5.13E-09 0.0244 prot-a-1495 || id:prot-a-1495 TRUE 0.010255244 34.18259935

4 IL1RN rs115995177 T C T C 0.4206 1.00E-04 0.02975 0.02365 finn-b-K11_ACUTPANC 5 7912602 0.0885 NA 0.9992 Acute pancreatitis || id:finn-b-K11_ACUTPANC Acute pancreatitis Acute pancreatitis || || TRUE igd 7912602 5 3301 3.55E-07 0.0826 prot-a-1504 || id:prot-a-1504 TRUE 0.007793544 25.91285311

5 IL1RN rs6761276 C T C T -0.1907 -0.006 0.5777 0.5453 finn-b-K11_ACUTPANC 2 113832312 0.0266 NA 0.8207 Acute pancreatitis || id:finn-b-K11_ACUTPANC Acute pancreatitis Acute pancreatitis || || TRUE igd 113832312 2 3301 1.51E-14 0.0248 prot-a-1504 || id:prot-a-1504 TRUE 0.017597141 59.0928336

6 IL1RN rs7748962 A G A G -0.1684 -0.0091 0.77686 0.7913 finn-b-K11_ACUTPANC 6 43759927 0.0332 NA 0.7831 Acute pancreatitis || id:finn-b-K11_ACUTPANC Acute pancreatitis Acute pancreatitis || || TRUE igd 43759927 6 3301 4.57E-08 0.0308 prot-a-1504 || id:prot-a-1504 TRUE 0.008974741 29.87579925

7 IL6 rs10512159 A G A G 0.1915 -0.0588 0.18168 0.1366 finn-b-K11_ACUTPANC 9 87608999 0.0389 NA 0.1311 Acute pancreatitis || id:finn-b-K11_ACUTPANC Acute pancreatitis Acute pancreatitis || || TRUE igd 87608999 9 3301 5.89E-08 0.0353 prot-a-1539 || id:prot-a-1539 TRUE 0.008836654 29.41202572

8 IL6 rs10787907 C T C T 0.1625 0.0226 0.78304 0.7969 finn-b-K11_ACUTPANC 10 85210946 0.0331 NA 0.4951 Acute pancreatitis || id:finn-b-K11_ACUTPANC Acute pancreatitis Acute pancreatitis || || TRUE igd 85210946 10 3301 5.13E-08 0.0298 prot-a-1539 || id:prot-a-1539 TRUE 0.008927586 29.71741257

9 IL6 rs11872808 T C T C 0.2297 0.0277 0.11345 0.08996 finn-b-K11_ACUTPANC 18 4331629 0.0463 NA 0.5507 Acute pancreatitis || id:finn-b-K11_ACUTPANC Acute pancreatitis Acute pancreatitis || || TRUE igd 4331629 18 3301 2.19E-08 0.0411 prot-a-1539 || id:prot-a-1539 TRUE 0.009373521 31.21584804

10 IL6 rs528547975 C T C T 0.301 -0.0099 0.07072 0.04938 finn-b-K11_ACUTPANC 12 34837744 0.0619 NA 0.8725 Acute pancreatitis || id:finn-b-K11_ACUTPANC Acute pancreatitis Acute pancreatitis || || TRUE igd 34837744 12 3301 3.39E-09 0.0509 prot-a-1539 || id:prot-a-1539 TRUE 0.010482751 34.94895687

11 IL18 rs693918 G A G A 0.1911 -0.0371 0.5501 0.5705 finn-b-K11_ACUTPANC 2 31868877 0.0267 NA 0.164 Acute pancreatitis || id:finn-b-K11_ACUTPANC Acute pancreatitis Acute pancreatitis || || TRUE igd 31868877 2 3394 2.38E-11 0.0285 prot-b-21 || id:prot-b-21 TRUE 0.013073879 44.93405988

12 IL18 rs75649625 A G A G -0.2894 0.0172 0.2427 0.2923 finn-b-K11_ACUTPANC 11 112052194 0.0292 NA 0.556 Acute pancreatitis || id:finn-b-K11_ACUTPANC Acute pancreatitis Acute pancreatitis || || TRUE igd 112052194 11 3394 1.46E-21 0.0301 prot-b-21 || id:prot-b-21 TRUE 0.026514397 92.38640502

13 IL27 rs11599750 T C T C -0.1596 0.028 0.38 0.2872 finn-b-K11_ACUTPANC 10 101805442 0.0293 NA 0.34 Acute pancreatitis || id:finn-b-K11_ACUTPANC Acute pancreatitis Acute pancreatitis || || TRUE igd 101805442 10 3394 1.40E-10 0.0248 prot-b-14 || id:prot-b-14 TRUE 0.012055442 41.39104755

14 IL27 rs4905 G A G A -0.4872 0.0343 0.2937 0.3068 finn-b-K11_ACUTPANC 19 4237067 0.0288 NA 0.234 Acute pancreatitis || id:finn-b-K11_ACUTPANC Acute pancreatitis Acute pancreatitis || || TRUE igd 4237067 19 3394 1.64E-80 0.025 prot-b-14 || id:prot-b-14 TRUE 0.100637008 379.5583478

15 IL27 rs72816426 T C T C 1.0967 -0.0366 0.009 0.003858 finn-b-K11_ACUTPANC 16 56586657 0.2195 NA 0.8677 Acute pancreatitis || id:finn-b-K11_ACUTPANC Acute pancreatitis Acute pancreatitis || || TRUE igd 56586657 16 3394 6.58E-08 0.2026 prot-b-14 || id:prot-b-14 TRUE 0.008559563 29.2847021
