# Supplementary material for: IL27 and IL1RN are causally associated with acute pancreatitis: a Mendelian randomization study
Source: Aging (Albany NY). 2024 May 13;16(10):8572–84. doi: 10.18632/aging.205825 (PMC11164491; doi:10.18632/aging.205825)
Supplement: Supplementary Table 3 [file aging-16-205825-s003.doc]

Supplementary Table 3. GeneMANIA interactions.

| Gene 1 | Gene 2 | Weight | Network group | Network |
| --- | --- | --- | --- | --- |
| KRTAP5-8 | IL27 | 0.05308598 | Co-expression | Mallon-McKay-2013 |
| IL36A | IL1RN | 0.02257787 | Co-expression | Mallon-McKay-2013 |
| PRSS36 | IL27 | 0.05698574 | Co-expression | Mallon-McKay-2013 |
| PRSS36 | KRTAP5-8 | 0.04458222 | Co-expression | Mallon-McKay-2013 |
| IL1F10 | IRAK2 | 0.01252137 | Co-expression | Mallon-McKay-2013 |
| IL1A | IL1RN | 0.01340452 | Co-expression | Roth-Zlotnik-2006 |
| KRTAP5-8 | IL27 | 0.00224408 | Co-expression | Roth-Zlotnik-2006 |
| IL36A | IL1RN | 0.01156148 | Co-expression | Roth-Zlotnik-2006 |
| PRSS36 | IL27 | 0.00231145 | Co-expression | Roth-Zlotnik-2006 |
| PRSS36 | KRTAP5-8 | 0.00189662 | Co-expression | Roth-Zlotnik-2006 |
| IL1B | IL1RN | 0.0104455 | Co-expression | Ramaswamy-Golub-2001 |
| KRTAP5-8 | IL27 | 0.04915251 | Co-expression | Innocenti-Brown-2011 |
| PRSS36 | IL27 | 0.03107853 | Co-expression | Innocenti-Brown-2011 |
| PRSS36 | KRTAP5-8 | 0.02351858 | Co-expression | Innocenti-Brown-2011 |
| IL1RAP | IL6ST | 0.02311831 | Co-expression | Innocenti-Brown-2011 |
| IL1B | IL1RN | 0.02088402 | Co-expression | Alizadeh-Staudt-2000 |
| STAT5A | IL1RN | 0.03065769 | Co-expression | Alizadeh-Staudt-2000 |
| IL1B | IL1R2 | 0.0122291 | Co-expression | Dobbin-Giordano-2005 |
| IL1A | IL1B | 0.01414018 | Co-expression | Dobbin-Giordano-2005 |
| IL36RN | IL1R2 | 0.01878297 | Co-expression | Dobbin-Giordano-2005 |
| IL36RN | IL1B | 0.01099404 | Co-expression | Dobbin-Giordano-2005 |
| IL12A | EBI3 | 0.00601607 | Co-expression | Dobbin-Giordano-2005 |
| IL36G | IL1R2 | 0.01564215 | Co-expression | Dobbin-Giordano-2005 |
| IL36G | IL1B | 0.01096688 | Co-expression | Dobbin-Giordano-2005 |
| IL36G | IL1A | 0.01865819 | Co-expression | Dobbin-Giordano-2005 |
| IL36G | IL36RN | 0.01431434 | Co-expression | Dobbin-Giordano-2005 |
| IL6ST | IL1R1 | 0.01261488 | Co-expression | Rieger-Chu-2004 |
| IL6ST | IL1R1 | 0.01489993 | Co-expression | Boldrick-Relman-2002 |
| IL1B | IL1RN | 0.00984846 | Co-expression | Boldrick-Relman-2002 |
| IL1A | IL1B | 0.01468152 | Co-expression | Boldrick-Relman-2002 |
| IL1B | IL1RN | 0.0120293 | Co-expression | Arijs-Rutgeerts-2009 |
| IL1A | IL1B | 0.01119852 | Co-expression | Arijs-Rutgeerts-2009 |
| IL36A | IL36RN | 0.01031772 | Co-expression | Arijs-Rutgeerts-2009 |
| IL1RAP | IL1B | 0.01155837 | Co-expression | Arijs-Rutgeerts-2009 |
| STAT2 | EBI3 | 0.01726758 | Co-expression | Jiang-de Kok-2017 |
| KRTAP5-8 | IL27 | 0.01429201 | Co-expression | Jiang-de Kok-2017 |
| PRSS36 | IL27 | 0.01833027 | Co-expression | Jiang-de Kok-2017 |
| PRSS36 | KRTAP5-8 | 0.0179314 | Co-expression | Jiang-de Kok-2017 |
| STAT5A | IL1RN | 0.01385003 | Co-expression | Jiang-de Kok-2017 |
| STAT5A | EBI3 | 0.024684 | Co-expression | Jiang-de Kok-2017 |
| IL1B | IL1RN | 0.00939647 | Co-expression | Perou-Botstein-2000 |
| IL1A | IL1B | 0.02569756 | Co-expression | Wang-Cheung-2015 |
| IL1A | IL1R1 | 0.01236511 | Co-expression | Rosenwald-Staudt-2001 |
| STAT5A | IL1RN | 0.01744386 | Co-expression | Rosenwald-Staudt-2001 |
| IL1RAP | IL1B | 0.010984 | Co-expression | Ross-Perou-2001 |
| IL6ST | IL1R1 | 0.01156896 | Co-localization | Johnson-Shoemaker-2003 |
| IL36A | IL1A | 0.01877089 | Co-localization | Johnson-Shoemaker-2003 |
| IL12A | IL1R1 | 0.01102509 | Co-localization | Johnson-Shoemaker-2003 |
| IL12A | IRAK2 | 0.02539462 | Co-localization | Johnson-Shoemaker-2003 |
| IL36G | IRAK2 | 0.02273355 | Co-localization | Johnson-Shoemaker-2003 |
| IL36G | IL36A | 0.01611566 | Co-localization | Johnson-Shoemaker-2003 |
| IL1A | IL1R2 | 1 | Co-localization | Chen-Huang-2014 |
| IL27RA | IL1RN | 0.0022602 | Genetic Interactions | Lin-Smith-2010 |
| IL6ST | IL1RN | 0.00125841 | Genetic Interactions | Lin-Smith-2010 |
| IL1RAP | IL1RN | 0.00088732 | Genetic Interactions | Lin-Smith-2010 |
| IL1RAP | IL27RA | 0.00115856 | Genetic Interactions | Lin-Smith-2010 |
| IL12A | IL1RN | 0.00071177 | Genetic Interactions | Lin-Smith-2010 |
| IL12A | IL1R2 | 0.00092333 | Genetic Interactions | Lin-Smith-2010 |
| IL12A | IL6ST | 0.00051743 | Genetic Interactions | Lin-Smith-2010 |
| IL1R2 | IL1RN | 0.17414123 | Pathway | Wu-Stein-2010 |
| IL1R1 | IL1RN | 0.07899424 | Pathway | Wu-Stein-2010 |
| EBI3 | IL27 | 0.1169335 | Pathway | Wu-Stein-2010 |
| IL27RA | IL27 | 0.13543765 | Pathway | Wu-Stein-2010 |
| IL27RA | EBI3 | 0.1169335 | Pathway | Wu-Stein-2010 |
| IL6ST | IL27 | 0.03416205 | Pathway | Wu-Stein-2010 |
| IL6ST | EBI3 | 0.02949466 | Pathway | Wu-Stein-2010 |
| IL6ST | IL27RA | 0.03416205 | Pathway | Wu-Stein-2010 |
| IL1B | IL1RN | 0.06434028 | Pathway | Wu-Stein-2010 |
| IL1B | IL1R2 | 0.09243102 | Pathway | Wu-Stein-2010 |
| IL1B | IL1R1 | 0.04192872 | Pathway | Wu-Stein-2010 |
| STAT2 | IL27 | 0.06953289 | Pathway | Wu-Stein-2010 |
| STAT2 | EBI3 | 0.06003298 | Pathway | Wu-Stein-2010 |
| STAT2 | IL27RA | 0.06953289 | Pathway | Wu-Stein-2010 |
| STAT2 | IL6ST | 0.01753859 | Pathway | Wu-Stein-2010 |
| IL1A | IL1RN | 0.05521926 | Pathway | Wu-Stein-2010 |
| IL1A | IL1R2 | 0.07932781 | Pathway | Wu-Stein-2010 |
| IL1A | IL1R1 | 0.03598482 | Pathway | Wu-Stein-2010 |
| IL1A | IL6ST | 0.01472249 | Pathway | Wu-Stein-2010 |
| IRAK2 | IL1RN | 0.1191898 | Pathway | Wu-Stein-2010 |
| IRAK2 | IL1R1 | 0.07767259 | Pathway | Wu-Stein-2010 |
| IRAK2 | IL1B | 0.0632638 | Pathway | Wu-Stein-2010 |
| IRAK2 | IL1A | 0.05429539 | Pathway | Wu-Stein-2010 |
| IL1RAP | IL1RN | 0.09081969 | Pathway | Wu-Stein-2010 |
| IL1RAP | IL1R2 | 0.13047126 | Pathway | Wu-Stein-2010 |
| IL1RAP | IL1R1 | 0.0591846 | Pathway | Wu-Stein-2010 |
| IL1RAP | IL1B | 0.04820545 | Pathway | Wu-Stein-2010 |
| IL1RAP | IL1A | 0.04137175 | Pathway | Wu-Stein-2010 |
| IL1RAP | IRAK2 | 0.08930018 | Pathway | Wu-Stein-2010 |
| STAT5A | IL27 | 0.02290194 | Pathway | Wu-Stein-2010 |
| STAT5A | EBI3 | 0.01977296 | Pathway | Wu-Stein-2010 |
| STAT5A | IL27RA | 0.02290194 | Pathway | Wu-Stein-2010 |
| STAT5A | IL6ST | 0.00577666 | Pathway | Wu-Stein-2010 |
| IL12A | IL27 | 0.05576396 | Pathway | Wu-Stein-2010 |
| IL12A | EBI3 | 0.04814522 | Pathway | Wu-Stein-2010 |
| IL12A | IL27RA | 0.05576396 | Pathway | Wu-Stein-2010 |
| IL12A | IL6ST | 0.01406559 | Pathway | Wu-Stein-2010 |
| IL12A | IL1B | 0.02800164 | Pathway | Wu-Stein-2010 |
| IL12A | STAT5A | 0.00942945 | Pathway | Wu-Stein-2010 |
| IL1R2 | IL1RN | 0.530689 | Pathway | NCI_NATURE |
| IL1R1 | IL1RN | 0.21678853 | Pathway | NCI_NATURE |
| EBI3 | IL27 | 0.0804202 | Pathway | NCI_NATURE |
| IL27RA | IL27 | 0.08393034 | Pathway | NCI_NATURE |
| IL27RA | EBI3 | 0.08393034 | Pathway | NCI_NATURE |
| IL6ST | IL27 | 0.05334433 | Pathway | NCI_NATURE |
| IL6ST | EBI3 | 0.05334433 | Pathway | NCI_NATURE |
| IL6ST | IL27RA | 0.05567268 | Pathway | NCI_NATURE |
| IL1B | IL1R2 | 0.10951739 | Pathway | NCI_NATURE |
| IL1B | IL1R1 | 0.04473828 | Pathway | NCI_NATURE |
| STAT2 | IL27 | 0.17727742 | Pathway | NCI_NATURE |
| STAT2 | EBI3 | 0.17727742 | Pathway | NCI_NATURE |
| STAT2 | IL27RA | 0.18501513 | Pathway | NCI_NATURE |
| STAT2 | IL6ST | 0.11759165 | Pathway | NCI_NATURE |
| IL1A | IL1R2 | 0.21910988 | Pathway | NCI_NATURE |
| IL1A | IL1R1 | 0.08950724 | Pathway | NCI_NATURE |
| STAT5A | IL27 | 0.05092463 | Pathway | NCI_NATURE |
| STAT5A | EBI3 | 0.05092463 | Pathway | NCI_NATURE |
| STAT5A | IL27RA | 0.05314736 | Pathway | NCI_NATURE |
| STAT5A | IL6ST | 0.03377932 | Pathway | NCI_NATURE |
| IL12A | IL27 | 0.04110356 | Pathway | NCI_NATURE |
| IL12A | IL1R1 | 0.03191378 | Pathway | NCI_NATURE |
| IL12A | EBI3 | 0.04110356 | Pathway | NCI_NATURE |
| IL12A | IL27RA | 0.04289762 | Pathway | NCI_NATURE |
| IL12A | IL6ST | 0.02726481 | Pathway | NCI_NATURE |
| IL12A | IL1B | 0.02945064 | Pathway | NCI_NATURE |
| IL12A | STAT5A | 0.02602808 | Pathway | NCI_NATURE |
| IL1R2 | IL1RN | 0.54827714 | Physical Interactions | IREF-reactome |
| IL1R1 | IL1RN | 0.25691566 | Physical Interactions | IREF-reactome |
| IL1B | IL1R2 | 0.12461885 | Physical Interactions | IREF-reactome |
| IL1B | IL1R1 | 0.05839481 | Physical Interactions | IREF-reactome |
| IL1A | IL1R2 | 0.13844402 | Physical Interactions | IREF-reactome |
| IL1A | IL1R1 | 0.0648731 | Physical Interactions | IREF-reactome |
| IL1A | IL1B | 0.05352536 | Physical Interactions | IREF-reactome |
| IRAK2 | IL1R1 | 0.03236233 | Physical Interactions | IREF-reactome |
| IRAK2 | IL1B | 0.02670144 | Physical Interactions | IREF-reactome |
| IRAK2 | IL1A | 0.02966369 | Physical Interactions | IREF-reactome |
| IL1RAP | IL1R1 | 0.0878415 | Physical Interactions | IREF-reactome |
| IL1RAP | IL1B | 0.07247607 | Physical Interactions | IREF-reactome |
| IL1RAP | IL1A | 0.08051654 | Physical Interactions | IREF-reactome |
| IL1RAP | IRAK2 | 0.04016615 | Physical Interactions | IREF-reactome |
| IL1R2 | IL1RN | 0.54827714 | Physical Interactions | Vastrik-Stein-2007 |
| IL1R1 | IL1RN | 0.25691566 | Physical Interactions | Vastrik-Stein-2007 |
| IL1B | IL1R2 | 0.12461885 | Physical Interactions | Vastrik-Stein-2007 |
| IL1B | IL1R1 | 0.05839481 | Physical Interactions | Vastrik-Stein-2007 |
| IL1A | IL1R2 | 0.13844402 | Physical Interactions | Vastrik-Stein-2007 |
| IL1A | IL1R1 | 0.0648731 | Physical Interactions | Vastrik-Stein-2007 |
| IL1A | IL1B | 0.05352536 | Physical Interactions | Vastrik-Stein-2007 |
| IRAK2 | IL1R1 | 0.03236233 | Physical Interactions | Vastrik-Stein-2007 |
| IRAK2 | IL1B | 0.02670144 | Physical Interactions | Vastrik-Stein-2007 |
| IRAK2 | IL1A | 0.02966369 | Physical Interactions | Vastrik-Stein-2007 |
| IL1RAP | IL1R1 | 0.0878415 | Physical Interactions | Vastrik-Stein-2007 |
| IL1RAP | IL1B | 0.07247607 | Physical Interactions | Vastrik-Stein-2007 |
| IL1RAP | IL1A | 0.08051654 | Physical Interactions | Vastrik-Stein-2007 |
| IL1RAP | IRAK2 | 0.04016615 | Physical Interactions | Vastrik-Stein-2007 |
| IL1R2 | IL1RN | 1 | Physical Interactions | IREF-quickgo |
| IL12A | EBI3 | 0.70710677 | Physical Interactions | IREF-quickgo |
| EBI3 | IL27 | 0.8164966 | Physical Interactions | IREF-dip |
| IL1A | IL1R2 | 0.70710677 | Physical Interactions | IREF-dip |
| IL1RAP | IL1B | 0.42260972 | Physical Interactions | IREF-dip |
| IL12A | EBI3 | 0.2521531 | Physical Interactions | IREF-dip |
| IL1R2 | IL1RN | 0.43676844 | Physical Interactions | BIOGRID-SMALL-SCALE-STUDIES |
| IL1R1 | IL1RN | 0.12645708 | Physical Interactions | BIOGRID-SMALL-SCALE-STUDIES |
| EBI3 | IL27 | 0.49943793 | Physical Interactions | BIOGRID-SMALL-SCALE-STUDIES |
| IL27RA | IL27 | 0.49671668 | Physical Interactions | BIOGRID-SMALL-SCALE-STUDIES |
| IL27RA | EBI3 | 0.3508369 | Physical Interactions | BIOGRID-SMALL-SCALE-STUDIES |
| IL1B | IL1R1 | 0.08591349 | Physical Interactions | BIOGRID-SMALL-SCALE-STUDIES |
| IRAK2 | IL1R1 | 0.06932771 | Physical Interactions | BIOGRID-SMALL-SCALE-STUDIES |
| IL1RAP | IL1R2 | 0.22099425 | Physical Interactions | BIOGRID-SMALL-SCALE-STUDIES |
| IL1RAP | IL1R1 | 0.06398422 | Physical Interactions | BIOGRID-SMALL-SCALE-STUDIES |
| IL1R2 | IL1RN | 0.5356226 | Physical Interactions | IREF-innatedb |
| IL1R1 | IL1RN | 0.27778628 | Physical Interactions | IREF-innatedb |
| IL1B | IL1R2 | 0.23471612 | Physical Interactions | IREF-innatedb |
| IL1B | IL1R1 | 0.12172922 | Physical Interactions | IREF-innatedb |
| IL1A | IL1R2 | 0.20555653 | Physical Interactions | IREF-innatedb |
| IL1A | IL1R1 | 0.10660639 | Physical Interactions | IREF-innatedb |
| IRAK2 | IL1R1 | 0.11798159 | Physical Interactions | IREF-innatedb |
| IL1RAP | IL1R1 | 0.25544307 | Physical Interactions | IREF-innatedb |
| IL1RAP | IRAK2 | 0.30325243 | Physical Interactions | IREF-innatedb |
| IL12A | EBI3 | 0.72670543 | Physical Interactions | IREF-innatedb |
| SEC22C | IL1RN | 0.30084813 | Physical Interactions | Huttlin-Harper-2017 |
| IL1R1 | IL1RN | 0.6484811 | Physical Interactions | IREF-bind-translation |
| IL1B | IL1R1 | 0.6484811 | Physical Interactions | IREF-bind-translation |
| IL1R1 | IL1RN | 0.62289274 | Physical Interactions | IREF-bind |
| IL1B | IL1R1 | 0.62289274 | Physical Interactions | IREF-bind |
| SEC22C | IL1RN | 0.4468287 | Physical Interactions | Huttlin-Gygi-2015 |
| IL1R1 | IL1RN | 0.29444277 | Physical Interactions | IREF-matrixdb |
| EBI3 | IL27 | 0.6745104 | Physical Interactions | IREF-matrixdb |
| IL1B | IL1R1 | 0.21141398 | Physical Interactions | IREF-matrixdb |
| IL1A | IL1R2 | 0.5087998 | Physical Interactions | IREF-matrixdb |
| IL12A | EBI3 | 0.3650424 | Physical Interactions | IREF-matrixdb |
| IL1A | IL1R1 | 0.3007339 | Physical Interactions | IREF-spike |
| IRAK2 | IL1R1 | 0.5019113 | Physical Interactions | IREF-spike |
| IL1R2 | IL1RN | 0.09162429 | Physical Interactions | IREF-biogrid |
| IL1R1 | IL1RN | 0.12703025 | Physical Interactions | IREF-biogrid |
| EBI3 | IL27 | 0.7220492 | Physical Interactions | IREF-biogrid |
| IL27RA | IL27 | 0.20367679 | Physical Interactions | IREF-biogrid |
| IL27RA | EBI3 | 0.16658372 | Physical Interactions | IREF-biogrid |
| IL1B | IL1R1 | 0.11625928 | Physical Interactions | IREF-biogrid |
| IL1A | IL1R2 | 0.07261951 | Physical Interactions | IREF-biogrid |
| IRAK2 | IL1R1 | 0.05661649 | Physical Interactions | IREF-biogrid |
| IL1RAP | IL1R1 | 0.07815132 | Physical Interactions | IREF-biogrid |
| SEC22C | IL1RN | 0.23993918 | Physical Interactions | IREF-biogrid |
| IL1F10 | IL1R1 | 0.7962252 | Predicted | Wu-Stein-2010 |
| IL6ST | IL27 | 0.5017067 | Predicted | I2D-IntAct-Mouse2Human |
| IL1B | IL1R1 | 0.6630571 | Predicted | I2D-IntAct-Mouse2Human |
| IL1RAP | IL1R1 | 0.27697834 | Predicted | I2D-IntAct-Mouse2Human |
| IL12A | EBI3 | 1 | Predicted | I2D-IntAct-Mouse2Human |
| IL12A | EBI3 | 0.58846956 | Predicted | I2D-INNATEDB-Mouse2Human |
| IL1A | IL1R1 | 0.62415826 | Predicted | I2D-BioGRID-Mouse2Human |
| IL1RAP | IL1R1 | 0.42854023 | Predicted | I2D-BioGRID-Mouse2Human |
| EBI3 | IL27 | 0.5 | Predicted | I2D-MGI-Mouse2Human |
| IL27RA | IL27 | 0.5 | Predicted | I2D-MGI-Mouse2Human |
| IL27RA | EBI3 | 0.5 | Predicted | I2D-MGI-Mouse2Human |
| IL1R1 | IL1R2 | 0.04063399 | Shared protein domains | INTERPRO |
| IL27RA | EBI3 | 0.01405396 | Shared protein domains | INTERPRO |
| IL1B | IL1RN | 0.07149708 | Shared protein domains | INTERPRO |
| IL1A | IL1RN | 0.07149725 | Shared protein domains | INTERPRO |
| IL1A | IL1B | 0.11480919 | Shared protein domains | INTERPRO |
| IL36RN | IL1RN | 0.09265095 | Shared protein domains | INTERPRO |
| IL36RN | IL1B | 0.06995596 | Shared protein domains | INTERPRO |
| IL36RN | IL1A | 0.06995612 | Shared protein domains | INTERPRO |
| IL36A | IL1RN | 0.04815116 | Shared protein domains | INTERPRO |
| IL36A | IL1B | 0.0513188 | Shared protein domains | INTERPRO |
| IL36A | IL1A | 0.05131882 | Shared protein domains | INTERPRO |
| IL36A | IL36RN | 0.04711326 | Shared protein domains | INTERPRO |
| IL1RAP | IL1R2 | 0.04129364 | Shared protein domains | INTERPRO |
| IL1RAP | IL1R1 | 0.06078767 | Shared protein domains | INTERPRO |
| STAT5A | STAT2 | 0.06195999 | Shared protein domains | INTERPRO |
| IL1F10 | IL1RN | 0.07364186 | Shared protein domains | INTERPRO |
| IL1F10 | IL1B | 0.04618654 | Shared protein domains | INTERPRO |
| IL1F10 | IL1A | 0.04618657 | Shared protein domains | INTERPRO |
| IL1F10 | IL36RN | 0.0720545 | Shared protein domains | INTERPRO |
| IL1F10 | IL36A | 0.05285845 | Shared protein domains | INTERPRO |
| IL36B | IL1RN | 0.07368913 | Shared protein domains | INTERPRO |
| IL36B | IL1B | 0.07853644 | Shared protein domains | INTERPRO |
| IL36B | IL1A | 0.07853663 | Shared protein domains | INTERPRO |
| IL36B | IL36RN | 0.07210075 | Shared protein domains | INTERPRO |
| IL36B | IL36A | 0.05289238 | Shared protein domains | INTERPRO |
| IL36B | IL1F10 | 0.04760275 | Shared protein domains | INTERPRO |
| IL36G | IL1RN | 0.07364186 | Shared protein domains | INTERPRO |
| IL36G | IL1B | 0.04618654 | Shared protein domains | INTERPRO |
| IL36G | IL1A | 0.04618657 | Shared protein domains | INTERPRO |
| IL36G | IL36RN | 0.0720545 | Shared protein domains | INTERPRO |
| IL36G | IL36A | 0.05285845 | Shared protein domains | INTERPRO |
| IL36G | IL1F10 | 0.08084049 | Shared protein domains | INTERPRO |
| IL36G | IL36B | 0.04760275 | Shared protein domains | INTERPRO |
| IL1R1 | IL1R2 | 0.02603433 | Shared protein domains | PFAM |
| IL27RA | EBI3 | 0.01920519 | Shared protein domains | PFAM |
| IL6ST | EBI3 | 0.01402769 | Shared protein domains | PFAM |
| IL6ST | IL27RA | 0.01416873 | Shared protein domains | PFAM |
| IL1B | IL1RN | 0.10089646 | Shared protein domains | PFAM |
| IL1A | IL1RN | 0.10089646 | Shared protein domains | PFAM |
| IL1A | IL1B | 0.22233364 | Shared protein domains | PFAM |
| IL36RN | IL1RN | 0.11293584 | Shared protein domains | PFAM |
| IL36RN | IL1B | 0.10089646 | Shared protein domains | PFAM |
| IL36RN | IL1A | 0.10089646 | Shared protein domains | PFAM |
| IL36A | IL1RN | 0.11293584 | Shared protein domains | PFAM |
| IL36A | IL1B | 0.10089646 | Shared protein domains | PFAM |
| IL36A | IL1A | 0.10089646 | Shared protein domains | PFAM |
| IL36A | IL36RN | 0.11293584 | Shared protein domains | PFAM |
| IL1RAP | IL1R2 | 0.04065823 | Shared protein domains | PFAM |
| IL1RAP | IL1R1 | 0.0586745 | Shared protein domains | PFAM |
| STAT5A | STAT2 | 0.07682413 | Shared protein domains | PFAM |
| IL1F10 | IL1RN | 0.11293584 | Shared protein domains | PFAM |
| IL1F10 | IL1B | 0.10089646 | Shared protein domains | PFAM |
| IL1F10 | IL1A | 0.10089646 | Shared protein domains | PFAM |
| IL1F10 | IL36RN | 0.11293584 | Shared protein domains | PFAM |
| IL1F10 | IL36A | 0.11293584 | Shared protein domains | PFAM |
| IL36B | IL1RN | 0.11293584 | Shared protein domains | PFAM |
| IL36B | IL1B | 0.10089646 | Shared protein domains | PFAM |
| IL36B | IL1A | 0.10089646 | Shared protein domains | PFAM |
| IL36B | IL36RN | 0.11293584 | Shared protein domains | PFAM |
| IL36B | IL36A | 0.11293584 | Shared protein domains | PFAM |
| IL36B | IL1F10 | 0.11293584 | Shared protein domains | PFAM |
| IL36G | IL1RN | 0.11293584 | Shared protein domains | PFAM |
| IL36G | IL1B | 0.10089646 | Shared protein domains | PFAM |
| IL36G | IL1A | 0.10089646 | Shared protein domains | PFAM |
| IL36G | IL36RN | 0.11293584 | Shared protein domains | PFAM |
| IL36G | IL36A | 0.11293584 | Shared protein domains | PFAM |
| IL36G | IL1F10 | 0.11293584 | Shared protein domains | PFAM |
| IL36G | IL36B | 0.11293584 | Shared protein domains | PFAM |
