# Supplementary material for: IL27 and IL1RN are causally associated with acute pancreatitis: a Mendelian randomization study
Source: Aging (Albany NY). 2024 May 13;16(10):8572–84. doi: 10.18632/aging.205825 (PMC11164491; doi:10.18632/aging.205825)
Supplement: Supplementary Table 4 [file aging-16-205825-s004.doc]

Supplementary Table 4. GeneMANIA functions.

| Function | FDR | Genes in network | Genes in genome |
| --- | --- | --- | --- |
| growth factor receptor binding | 2.47E-11 | 9 | 106 |
| cytokine receptor binding | 7.71E-09 | 9 | 214 |
| cellular response to interleukin-1 | 6.86E-08 | 7 | 99 |
| response to molecule of bacterial origin | 1.11E-07 | 8 | 195 |
| response to lipopolysaccharide | 3.74E-07 | 7 | 135 |
| cellular response to molecule of bacterial origin | 3.83E-07 | 7 | 139 |
| regulation of T cell activation | 4.94E-07 | 8 | 252 |
| regulation of interferon-gamma production | 5.11E-07 | 6 | 77 |
| response to interleukin-1 | 5.43E-07 | 7 | 159 |
| regulation of lymphocyte proliferation | 5.43E-07 | 7 | 163 |
| interferon-gamma production | 5.43E-07 | 6 | 82 |
| cellular response to biotic stimulus | 5.43E-07 | 7 | 161 |
| positive regulation of T cell activation | 5.43E-07 | 7 | 162 |
| regulation of mononuclear cell proliferation | 5.49E-07 | 7 | 165 |
| regulation of leukocyte proliferation | 8.72E-07 | 7 | 178 |
| positive regulation of leukocyte cell-cell adhesion | 1.15E-06 | 7 | 187 |
| cytokine binding | 1.19E-06 | 6 | 100 |
| positive regulation of mononuclear cell proliferation | 1.19E-06 | 6 | 101 |
| lymphocyte proliferation | 1.45E-06 | 7 | 198 |
| mononuclear cell proliferation | 1.58E-06 | 7 | 202 |
| positive regulation of leukocyte proliferation | 1.62E-06 | 6 | 109 |
| positive regulation of cell-cell adhesion | 2.29E-06 | 7 | 216 |
| positive regulation of lymphocyte activation | 2.46E-06 | 7 | 221 |
| leukocyte proliferation | 2.46E-06 | 7 | 221 |
| T cell proliferation | 3.78E-06 | 6 | 129 |
| immune receptor activity | 4.18E-06 | 6 | 132 |
| regulation of leukocyte cell-cell adhesion | 5.75E-06 | 7 | 254 |
| positive regulation of leukocyte activation | 6.01E-06 | 7 | 257 |
| positive regulation of cell activation | 6.81E-06 | 7 | 263 |
| leukocyte cell-cell adhesion | 8.73E-06 | 7 | 274 |
| cytokine receptor activity | 1.49E-05 | 5 | 80 |
| positive regulation of cell adhesion | 1.49E-05 | 7 | 299 |
| adaptive immune response | 2.56E-05 | 6 | 186 |
| positive regulation of lymphocyte proliferation | 3.81E-05 | 5 | 98 |
| positive regulation of T cell proliferation | 3.81E-05 | 4 | 36 |
| regulation of T cell proliferation | 9.8E-05 | 5 | 119 |
| regulation of adaptive immune response | 0.000108 | 5 | 122 |
| adaptive immune response based on somatic recombination of immune receptors built from immunoglobulin superfamily domains | 0.000579 | 5 | 172 |
| regulation of T-helper 1 type immune response | 0.000585 | 3 | 19 |
| T-helper 1 type immune response | 0.001039 | 3 | 23 |
| regulation of vascular endothelial growth factor production | 0.001485 | 3 | 26 |
| vascular endothelial growth factor production | 0.001629 | 3 | 27 |
| growth factor binding | 0.001658 | 4 | 96 |
| regulation of adaptive immune response based on somatic recombination of immune receptors built from immunoglobulin superfamily domains | 0.00289 | 4 | 111 |
| receptor signaling pathway via STAT | 0.006654 | 4 | 138 |
| positive regulation of T cell mediated immunity | 0.006654 | 3 | 44 |
| T cell differentiation | 0.008663 | 4 | 149 |
| regulation of T cell mediated immunity | 0.019771 | 3 | 64 |
| positive regulation of T cell cytokine production | 0.021419 | 2 | 10 |
| positive regulation of adaptive immune response | 0.023316 | 3 | 69 |
| positive regulation of adaptive immune response based on somatic recombination of immune receptors built from immunoglobulin superfamily domains | 0.023316 | 3 | 69 |
| T cell mediated immunity | 0.029344 | 3 | 75 |
| positive regulation of lymphocyte mediated immunity | 0.029951 | 3 | 76 |
| regulation of alpha-beta T cell activation | 0.030566 | 3 | 77 |
| CD4-positive, alpha-beta T cell cytokine production | 0.044375 | 2 | 15 |
| lymphocyte differentiation | 0.044568 | 4 | 238 |
| positive regulation of leukocyte mediated immunity | 0.044568 | 3 | 89 |
| alpha-beta T cell activation | 0.065602 | 3 | 102 |
| regulation of lymphocyte mediated immunity | 0.091869 | 3 | 115 |
| negative regulation of signal transduction in absence of ligand | 0.097483 | 2 | 23 |
